# Social status and parasitism in male and female vertebrates: a meta-analysis

**DOI:** 10.1038/s41598-018-21994-7

**Published:** 2018-02-26

**Authors:** Bobby Habig, Meredith M. Doellman, Kourtney Woods, Jonathan Olansen, Elizabeth A. Archie

**Affiliations:** Department of Biological Sciences, University of Notre Dame, 100 Galvin Life Sciences Center, Notre Dame, IN 46556 USA

## Abstract

Social status is an important predictor of parasite risk in vertebrates. To date, general frameworks to explain status-related variation in parasitism have remained elusive. In this meta-analysis, we evaluated five hypotheses proposed to explain status-related variation in parasitism in male and female vertebrates by leveraging variation in hierarchy type, mating system, parasite transmission mode, and allostatic load to test associated predictions. Our meta-analyses span 66 analyses (26 studies) of male vertebrates (two orders and five classes), and 62 analyses (13 studies) of female vertebrates (four vertebrate orders). Contrary to the prevailing paradigm that low status is linked to poor health, we found that dominant animals typically faced higher parasite risk than subordinates. This pattern was especially well-supported in analyses of males versus females, in linear versus egalitarian hierarchies, in mating systems where dominance rank predicts mating effort, and for contact- and environmentally-transmitted parasites rather than vector-borne parasites. These findings supported the priority-of-access and tradeoffs hypotheses suggesting that variation in parasitism is driven by rank-associated differences in exposure to parasites and mating effort. Together, these results suggest that high parasite risk might sometimes be an unappreciated cost of high rank, and conversely, reduced parasite risk might be a benefit of social subordination.

## Introduction

Social status can have profound consequences for vertebrate health^1–3^. In humans, low socioeconomic status is a leading predictor of chronic disease and reduced longevity^2,4^. In non-human animals, individuals at the bottom of a hierarchy often have to work harder for calories, receive less social support, and are subject to displacement and aggression from dominants^1^. As such, social subordination in mice, birds, and non-human primates is often linked to poor physical condition and a range of health problems, including hypertension, slow wound healing, inflammation, and poor immune responses^1,5–7^. However, whether social subordination also predicts patterns of parasitism remains an open question. This relationship is enigmatic, in part, because variation in parasite infection is driven, not only by differences in host susceptibility, but also by differences in parasite exposure. Here, we evaluate major hypotheses proposed to explain how host susceptibility and parasite exposure drive status-related differences in parasitism. To do so, we test predictions that rely on differences in host dominance structures and mating systems, as well as variation in parasite transmission mode.

To date, five hypotheses have been proposed to explain status-related variation in parasitism (Table S1). Two of these hypotheses predict high parasitism in low-status animals. First, the *condition-dependent hypothesis* posits that status-related differences in physical condition lead to differences in parasite susceptibility and resistance^8,9^. If hierarchy position is linked to strong differences in physical quality, either because hierarchy position determines access to resources, or because an animal’s strength and physical condition determines their hierarchy position, animals that are stronger and healthier may rise to the top of the hierarchy, have the best access to resources, and therefore exhibit the lowest parasite burdens. In contrast, animals in poor condition may be restricted to the bottom of the hierarchy, have less access to resources, and be more susceptible to parasite infection. Second, the *stress-response hypothesis* proposes that the experience of low social status, including high rates of received aggression and unpredictable access to resources, can lead to chronic stress, which alters immune responses in ways that make low-status animals more susceptible to infection than high-status animals^10–12^. In support, studies have found that subordinate animals exhibit higher glucocorticoid levels than dominants, which can inhibit immune defenses against parasites^7,13^.

In contrast to the stress-response and condition-dependent hypotheses, two hypotheses predict that high-ranking animals will be more parasitized than low-ranking animals. First, the *priority-of-access hypothesis* proposes that dominant individuals have greater access to food^14,15^ and mates^16–18^, which in turn leads to higher rates of parasite exposure relative to subordinates^6,19,20^. In support, several studies have found that high-ranking individuals have higher mating success and the best access to nutritional resources and subsequently harbor more parasites than low-ranking individuals (e.g.^16,21,22^). Second, the *tradeoffs hypothesis* proposes that investment in reproductive effort by dominant individuals is negatively correlated with costly immune defenses, which increases susceptibility to parasites^20,23^. In support, several studies have found that the reproductive effort exerted by high-ranking males and females is associated with higher rates of parasitism compared to lower-ranking same-sex conspecifics (e.g.^24–26^). A variant of the tradeoffs hypothesis is the immunocompetence handicap hypothesis (ICHH), which posits that high-ranking individuals, typically males, maintain higher levels of androgens and parasitism than low-ranking individuals, which reflects an honest indicator of quality^27^. To date, the ICHH has yielded limited support^28,29^.

The *allostatic load model* integrates ideas from the tradeoffs, stress-response, and condition-dependent hypotheses^30^. The *allostatic load hypothesis*, which is adapted from previous work on humans^31^, posits that differences in allostatic load, defined as the cumulative physiological burdens exerted on the body to meet life history demands, predict status-related variation in parasitism. Within a species and sex, the rank that experiences the highest allostatic load is expected to exhibit the highest parasitism, and when ranks do not differ in allostatic load, there should be no status-associated differences in parasitism. Goymann and Wingfield^32^ found that the relative allostatic load of high- and low-ranking animals predicted their relative glucocorticoid levels. However, no studies have yet linked allostatic load to parasite risk in a meta-analytic framework.

It is unlikely that any single hypothesis explains status-related differences in parasitism across vertebrates. This is because variation in animal societies and parasite life histories can have important consequences for patterns of susceptibility, exposure, and transmission^15,19^. For instance, differences in the structure and form of animal hierarchies can influence susceptibility and exposure for both dominants and subordinates^20,23^. Specifically, in linear hierarchies (defined in Table S2), where resource access is highly skewed in favor of dominant individuals, dominants are expected to be more exposed to parasites than subordinates. Alternatively, in egalitarian hierarchies (Table S2), where resources are more equally distributed, dominants and subordinates are expected to exhibit similar levels of parasitism. Moreover, the type of mating system might also affect the association between dominance rank and parasitism. In mating systems in which dominants invest more in reproductive effort than subordinates, high-ranking individuals are expected to harbor more parasites than low-ranking individuals^26^. For example, in some cooperative breeding mating systems, dominants invest more in reproductive effort than subordinates, and the energetic costs associated with such an effort results in compromised immunity and increased parasitism (e.g.^26^). Conversely, in mating systems associated with subordinate social stress, high-ranking individuals are predicted to harbor fewer parasites than low-ranking individuals^33^. For example, in some polygynous mating systems (i.e. mating systems in which a single male mates with multiple females during a mating period), submissive males who are recipients of aggression harbor more parasites than dominants (e.g.^33^). Thus, variation in both hierarchy type and mating system might influence patterns of exposure, transmission, and susceptibility among high- and low-ranking conspecifics.

Parasite transmission mode may also influence the link between host social status and parasite infection risk because differences in behavior between high- and low-status animals can create heterogeneities in parasite exposure^15,34^. For instance, if high-ranking individuals have priority of access to mates or food, they may experience greater exposure to contact- and environmentally-transmitted parasites, but not parasites transmitted by flying-vectors, as compared to subordinates (e.g.^16,21,22^). However, if status-related differences in behavior drive differences in parasite susceptibility, for instance through nutritional limitation^35^, then low-status animals should be more susceptible to parasite infection, regardless of parasite transmission mode. Thus, rank-associated variation in parasitism is likely to be driven by the interplay between parasite transmission mode and host social behavior.

Our objective was to test leading hypotheses proposed to explain status-related differences in parasitism using multi-level meta-analytic models. We began by testing broad patterns of parasitism separately for each sex: if high-ranking males and females harbor more parasites than low-ranking same-sex conspecifics, then this pattern would support the tradeoffs and priority-of-access hypotheses. Alternatively, if low-ranking animals exhibit higher parasitism than high-ranking animals, this pattern would support the stress-response and condition-dependent hypotheses. Following these broad analyses, we tested specific predictions of each hypothesis in each sex based on variation in (i) types of dominance hierarchy, (ii) mating systems, and (iii) parasite transmission modes (Tables S1–S2). Further, we tested the allostatic load model by calculating allostatic load scores for dominant and subordinate males and females^32^ and correlating these scores with parasite burdens: if relative allostatic load is correlated with relative parasitism, then this pattern would support the allostatic load hypothesis. Because general frameworks explaining the link between social status and parasitism have remained elusive, our results are an important step forward in understanding the primary drivers of status-related variation in parasitism.

## Results

### Studies of social status and parasitism

Our literature search yielded 26 studies and 66 distinct analyses for males and 13 studies and 62 distinct analyses for females. Seven taxonomic groups (two orders and five classes) were represented in analyses of males; four orders were represented in analyses of females (Table S3). 91% (n = 60) of male analyses occurred in wild settings (without provisioning) and 9% (n = 6) took place in captivity. For female analyses, 66% (n = 41) occurred in wild settings without provisioning, 32% (n = 20) occurred in wild settings with provisioning, and 2% (n = 1) took place in captivity. Overall, there were six different methods employed to measure dominance rank (Table S4).

### High-status males face higher parasite risk than low-status males

**Figure 1:**
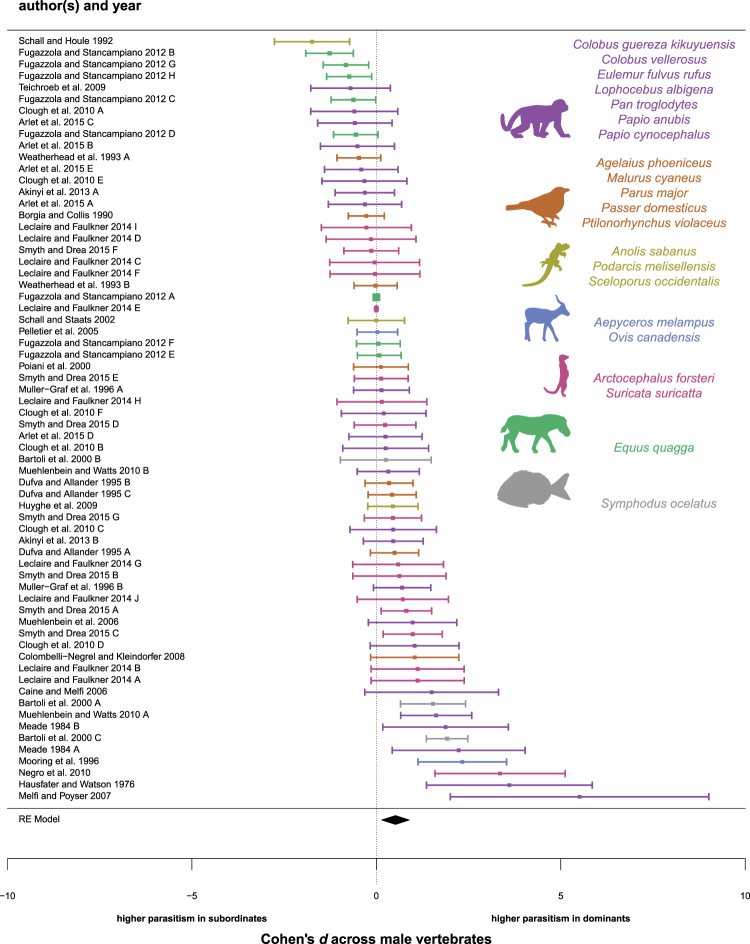
A forest plot showing the effect sizes of all male studies that tested the association between social status and parasitism. Positive values indicate higher parasitism in dominants; negative values indicate higher parasitism in subordinates. The error bars represent the 95% CI lower limit and the 95% CI upper limit; the square represents the effect size (*d*) of each study. The center of the diamond represents the effect size and its length represents the 95% CI for all male studies. Purple = Order Primates^A^; Orange = Class Aves^B^; Yellow = Order Squamata^C^; Blue = Order Perissodactyla^D^; Pink = Order Carnivora^E^; Green = Order Artiodactyla^F^; Gray = Order Actinopterygii^G^. Silhouettes are adapted from “Monkey”^A^, “Zebra”^D^, “Meerkat”^E^, and “Impala”^F^ icons by Anniken & Andreas, “Sparrow”^B^ icon by Hernan D. Schlosman, “Lizard”^C^ icon by B. Agustín Amenábar Larraín, and “Fish”^G^ icon by Aleksandr Vector from the Noun Project.

Contrary to the condition-dependent and stress-response hypotheses (Table S1), high-ranking males typically faced higher parasite risk than low-ranking males (Fig. 1; Tables 1, S5). Across all male analyses, dominant males exhibited significantly higher parasitism than subordinate males (d = 0.511; 95% CI: 0.130 to 0.974, p = 0.005). However, we found high levels of total heterogeneity in our best-supported model (I^2^ = 99.19; 95% CI: 98.46–99.77), which indicates that the association between social status and parasitism is not uniform for all species. Furthermore, an Egger’s test revealed significant publication bias: the trim and fill method estimated that there was one fewer published analysis than expected with a small sample size and where subordinates had higher parasitism than dominants (Table 1; Fig. S1).

**Table 1:** Summary of meta-analyses testing status-related differences in parasitism in male and female vertebrates. Models presented in this table represent the best-supported models based on k-fold cross validation. Variance estimates are reported as standard deviations of the random effects.

type of meta-analysis^A^	sample size (analyses)	random effects included	kfoldIC	standard difference in means	95% CI lower limit	95% CI upper limit	*p* ^B^	variance estimate (standard deviation)			total heterogeneity (I^2^) with credible intervals	higher in dominant or subordinate	Egger’s test	citations
								study	species	phylogeny			(p-value)	
Male Studies														
all male studies	66	study only	152.204	0.511	0.130	0.974	0.005	0.875	—	—	99.19 (98.46–99.77)	dominant	<0.001	^24–26,33,38,43,81–100^
males in despotic hierarchies	59	study and species	140.225	0.493	0.013	1.001	0.023	0.677	0.600	—	99.40 (98.83–99.82)	dominant	<0.001	^24–26,33,38,43,81–84,86–100^
males in egalitarian hierarchies	6	NA^C^	12.112	0.170	−0.532	0.902	0.300	NA^C^	NA^C^	NA^C^	24.06 (0.01–78.97)	neither	0.304	^85^
males in polygynandrous mating systems	29	study only	79.085	0.943	0.348	1.671	0.001	0.938	—	—	78.29 (59.92–95.22)	dominant	0.002	^24,25,38,81,82,84,85,88,90–94,96,100^
males in cooperative breeding mating systems	18	study only	29.307	0.390	−1.573	2.344	0.161	1.098	—	—	71.27 (27.28–99.97)	neither	<0.001	^26,43,89^
males in polygynous mating systems	15	study only	41.34	−0.168	−1.272	1.114	0.322	1.207	—	—	94.42 (83.25–99.93)	neither	0.242	^33,83,87,95,98–100^
males in monogamous mating systems	4	study only	2.787	0.229	−6.677	6.209	0.355	3.111	—	—	84.91 (28.92–99.99)	neither	0.496	^86,97^
males exposed to environmentally- and contact-transmitted parasites	53	study only	122.344	0.704	0.238	1.276	0.002	0.960	—	—	99.44 (98.91–99.85)	dominant	<0.001	^24–26,38,81–85,87–97,100^
males exposed to parasites transmitted by flying-vectors	6	study and species	15.507	−0.104	−4.244	3.914	0.469	2.018	2.046	—	95.29 (83.32–99.99)	neither	0.634	^33,43,86,98^
Female Studies														
all female studies	62	study only	114.020	0.322	−0.059	0.737	0.044	0.611	—	—	98.33 (96.56–99.68)	dominant	0.118	^24,26,81,87,89,90,94,101–106^
females in despotic nepotistic hierarchies	44	study, species, phylogeny	92.562	0.168	−2.127	2.377	0.365	0.616	0.355	1.070	92.16 (78.68–99.88)	neither	0.050	^24,81,87,88,90,92,94,97,101–105^
females in despotic age-based hierarchies	18	study	23.405	0.324	−2.549	3.371	0.309	—	—	—	90.16 (66.83–99.99)	neither	0.170	^26,89,106^
females in polygynandrous mating systems	29	study and species	62.579	0.457	−0.571	1.447	0.121	0.755	0.585	—	74.52 (46.91–98.92)	neither	0.199	^24,81,90,94,102–105^
females in cooperative breeding mating systems	17	study	19.646	0.547	−5.953	6.788	0.286	3.460	—	—	92.40 (67.09–99.99)	neither	0.044	^26,89^
females in polygynous mating systems	16	study	32.001	−0.266	−1.488	0.882	0.166	0.646	—	—	74.78 (34.55–99.99)	neither	0.040	^87,101,106^
females exposed to environmentally- and contact-transmitted parasites	61	study	108.234	0.371	−0.006	0.808	0.027	0.605	—	—	98.30 (96.41–99.67)	dominant	0.120	^24,26,81,87,89,90,94,101–105^

^A^In all of the above models, study setting (wild non-provisioned, wild provisioned, captive) did not significantly improve model fit and was removed from all final models. ^B^The p-values generated by the *brms* package^36^ are 1-tailed, while the credible intervals are 2-tailed ^C^NA = not applicable (All effect sizes for egalitarian hierarchies were from the same study and species).

Despite evidence of nonuniformity, and with the caveat of minor publication bias, high-status males exhibited higher parasite burdens than low-status males in the majority of studies: we found positive effect sizes (i.e. higher parasitism in high-status than low-status males) in 41 of 66 analyses (62.1%), negative effect sizes in 22 analyses (33.3%), and an effect size of zero in three analyses (4.5%). This pattern persisted when we analyzed each measure of parasitism separately (Table S5): high-ranking males exhibited higher parasite richness (d = 0.498, 95% CI: −0.103 to 1.102, p = 0.043; n = 9), intensity (d = 0.590, 95% CI: 0.021 to 1.316, p = 0.022; n = 30), and prevalence (d = 1.497, 95% CI: 0.358 to 2.586, p = 0.010; n = 26) than low-ranking males. Because the association between social status and parasitism is unlikely to be uniform across species, we separately analyzed each host clade (Fig. S2), and found that high-ranking males exhibited significantly higher parasitism than low-ranking males in the class Mammalia (d = 0.742, 95% CI: 0.042 to 1.569, p = 0.020; n = 52) and in the order Primates (d = 0.826, 95% CI: 0.043 to 1.848, p = 0.020; n = 24), but there were no rank-associated differences in parasitism for the remaining clades, some of which had small sample sizes (e.g. Squamata: n = 3; Aves: n = 6; Diapsida: n = 11; Table S6). When we modeled study setting (wild non-provisioned, captive) as a fixed effect, other than for parasite prevalence (Table S5), this variable did not significantly improve model fit for any of the other aforementioned analyses. Hence, with the exception of parasite prevalence, study setting was removed from the final model for these analyses (Table 1, Tables S5–S6).

The observation that high-status males exhibited the highest parasite risk supported the tradeoffs and priority-of-access hypotheses. If priority of access to resources increases parasite exposure, we would expect to observe status-related differences in parasitism (i) in linear but not egalitarian hierarchies, and (ii) for contact- and environmentally-transmitted parasites but not vector-borne parasites^6,15,19,20^. In support, high-ranking males in linear despotic hierarchies exhibited higher parasitism than low-ranking males (d = 0.493, 95% CI: 0.013 to 1.001, p = 0.023; n = 59), while social status did not predict male parasitism in the one study of an egalitarian species (d = 0.170, 95% CI: −0.532 to 0.902, p = 0.300; n = 6 analyses). Further, we found that dominant males had higher burdens of environmentally- and contact-transmitted parasites (d = 0.704, 95% CI: 0.238–1.276, p = 0.002; n = 53) than subordinate males, but dominance rank did not predict differences in infection risk for parasites transmitted by flying-vectors (d = −0.104, 95% CI: −4.244 to 3.914, p = 0.469; n = 6). For the above analyses, study setting did not significantly improve model fit, and was subsequently removed from our final models (Tables 1, S5).

The tradeoffs hypothesis predicts higher parasitism in high-ranking males in mating systems and dominance hierarchies in which social status predicts mating effort (despotic hierarchies; cooperative breeding, polygynandrous, and polygynous mating systems), but not in mating systems and dominance hierarchies where social status is not a strong predictor of mating effort (egalitarian hierarchies; monogamous mating systems)^20,23^. In support, high-status males in hierarchies and mating systems associated with high mating effort harbored significantly greater parasites than low-status males (polygnous + polygynandrous + cooperative breeding: d = 1.928, CI: 0.439 to 3.533, p = 0.007; n = 62; Table S5). Furthermore, social status was not linked to predictable differences in parasitism in hierarchy types and mating systems associated with low mating effort (egalitarian + monogamous: d = 0.270, 95% CI: −1.265 to 1.841, p = 0.207; n = 10; Table S5). However, while combined analyses of mating systems and hierarchy types supported the tradeoffs hypothesis, there were no significant differences in parasitism between dominants and subordinates when males from polygynous hierarchies (d = −0.168, 95% CI: −1.272 to 1.114, p = 0.322; n = 15) or cooperative breeding systems (d = 0.390, 95% CI: −1.573 to 2.344, p = 0.161, n = 18) were analyzed separately (Table 1). For the above analyses, with the exception of one analysis (polygnous + polygynandrous + cooperative breeding), study setting was removed from the final model because it did not significantly improve model fit (Tables 1, S5).

### A non-significant trend for higher parasitism in high-status than low-status females

**Figure 2:**
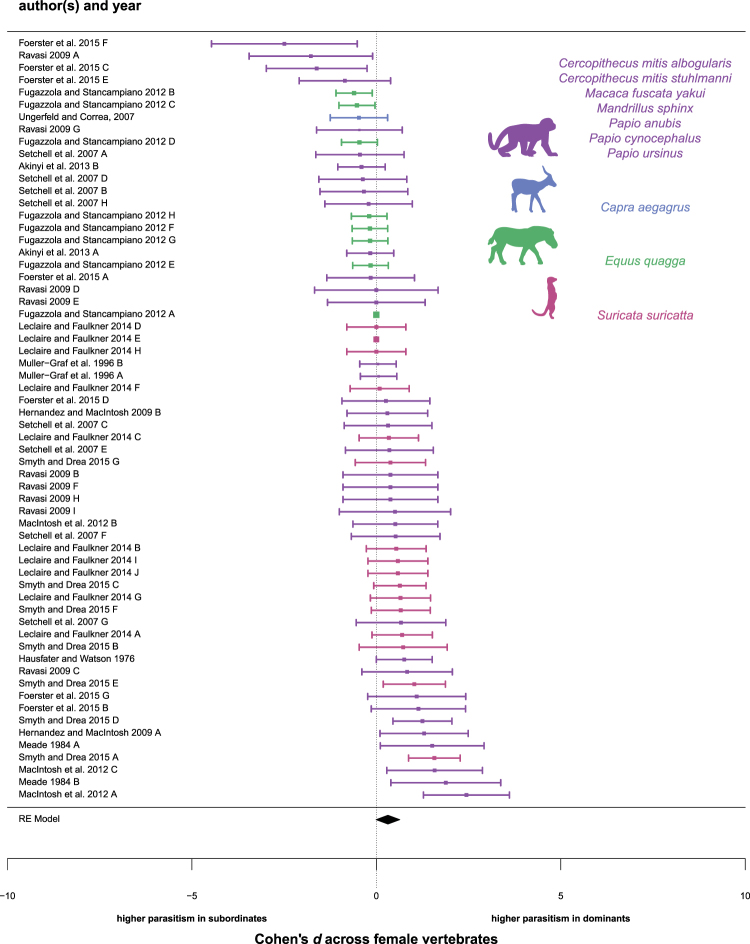
A forest plot showing the effect sizes of all female studies that tested the association between social status and parasitism. Positive values indicate higher parasitism in dominants; negative values indicate higher parasitism in subordinates. The error bars represent the 95% CI lower limit and the 95% CI upper limit; the square represents the effect size (*d*) of each study. The center of the diamond represents the effect size and its length represents the 95% CI for all female studies. Purple = Order Primates^A^; Blue = Order Perissodactyla^B^; Green = Order Artiodactyla^C^; Pink = Order Carnivora^D^. Silhouettes are adapted from “Monkey”^A^, “Impala”^B^, “Zebra”^C^, and “Meerkat”^D^ icons by Anniken & Andreas from the Noun Project.

Dominant females frequently exhibited higher levels of parasitism than subordinate females, although the relationship was not statistically significant as the credible intervals overlapped with zero (d = 0.322, 95% CI: −0.059 to 0.737, p = 0.044; n = 62; Fig. 2; Table 1; note that the credible intervals generated by the *brms* package are 2-tailed^36^ while the p-values are 1-tailed). Similar to males, we found high levels of total heterogeneity in our best supported model (I^2^ = 98.33; 95% CI: 96.56 to 99.68), again indicating that the association between social status and parasitism is not uniform for all species. Unlike the male studies, we did not observe evidence of publication bias in female studies (Table 1; Fig. S3). For all female analyses, study setting (wild non-provisioned, wild provisioned, captive) did not significantly improve model fit, and therefore was removed from our final models.

We found positive effect sizes in 36 of 62 analyses (58.1%), negative effect sizes in 20 analyses (32.3%), and an effect size of zero in six analyses (9.7%). In sub-analyses for each parasitological measure (Table S7), we found a non-significant trend indicating a positive association between dominance rank and parasite richness (d = 1.382, 95% CI: −1.507 to 4.180, p = 0.091, n = 5); there were no status-related differences based on measures of parasite intensity (d = 0.115, 95% CI: −0.221 to 0.536, p = 0.266, n = 28) or prevalence (d = 0.192, 95% CI: −0.299 to 0.707, p = 0.170, n = 29). When each host clade was separately analyzed (Fig. S4), we found a non-significant trend in which dominant females exhibited higher levels of parasitism than subordinate females in Primates (d = 0.390; 95% CI: −0.110 to 0.951, p = 0.055; n = 36), especially in Cercopithecidae (*Papio* + *Mandrillus* + *Macaca;* d = 0.491, 95% CI: −0.080 to 1.138, p = 0.040; n = 29). There were no consistent rank-associated differences in parasitism for the remaining clades (Table S8).

We found limited support for the priority-of-access hypothesis in analyses of females. While our data did not include any females living in egalitarian societies, we were able to test status-related differences in parasitism for females in linear hierarchies. Contrary to the priority-of-access hypothesis, there were no status-related differences in parasitism for either age-based or nepotistic linear hierarchies (age-based: d = 0.324, 95% CI: −2.549 to 3.371, p = 0.309, n = 18; nepotistic: d = 0.168, 95% CI: −2.127 to 2.377, p = 0.365; n = 44; Table 1). However, we identified a positive trend indicating that dominant females harbored higher environmentally- and contact-transmitted parasites than subordinate females (d = 0.371, 95% CI: −0.006 to 0.808, p = 0.027; n = 61, Table 1). No female studies measured status-related differences in parasites transmitted by flying-vectors.

While tradeoffs between reproductive effort and immune defense are typically measured in males, high-ranking females may engage in higher reproductive effort than low-ranking females, at least in some cooperative breeding and polygynandrous mating systems^37^. In support of the tradeoffs hypothesis, high-status females harbored significantly more parasites than low-status females in mating systems associated with high reproductive effort (polygynandrous + cooperative breeding: d = 0.508, 95% CI: 0.067 to 1.004, p = 0.015; n = 46; Table S7). However, this effect was not significant when we tested the relationship in cooperative breeders alone (d = 0.547, 95% CI: −5.953 to 6.788, p = 0.286; n = 17; Table 1). As expected, there was no significant difference in parasitism between dominant and subordinate females in polygynous mating systems (d = −0.266, 95% CI: −1.488 to 0.882, p = 0.166; n = 16; Table 1).

### Status-related differences in allostatic load do not predict differences in parasitism

Contrary to the allostatic load hypothesis, across 50 available male analyses, we found no significant correlation between relative allostatic load and relative parasitism (r = −0.014; p = 0.922; Fig. S5). Likewise, across 48 available female analyses, there was no significant correlation between relative allostatic load and relative parasitism (r = −0.038, p = 0.802; Fig. S6).

## Discussion

A dominant paradigm in the scientific literature has been that subordinate social status is linked to poorer health and immunity^2,11,12^. However, whether social subordination also predicts infectious disease risk, has remained an open question. Overall, our meta-analyses revealed that individuals at the top of the hierarchy, rather than subordinates, often bear the highest parasite burdens. This pattern was most evident in male mammals, in linear hierarchies, in mating systems where rank predicts mating effort, and for contact- and environmentally-transmitted parasites. Of the five models evaluated, we found general support for the priority-of-access and tradeoffs hypotheses, both of which predict higher parasitism in high-ranking individuals, but little or no support for the condition-dependent, stress-response, or allostatic load hypotheses. Together, these results suggest that social subordination is not always bad for health, and parasite risk is sometimes an underappreciated cost of high dominance rank.

Given the support we found for the priority-of-access and tradeoffs hypotheses, we propose that rank-associated differences in parasitism are likely explained by two key drivers: (i) greater exposure to parasites in high-ranking compared to low-ranking hosts due to status-associated differences in access to resources; and (ii) greater susceptibility to parasitism in high-ranking compared to low-ranking hosts due to status-associated differences in mating effort^15,20,23^. In support of the first driver, several studies have found a link between social status, access to resources, and parasitism (e.g.^16,21,22^). For example, dominant, territorial male occelated wrasses (*Symphodus ocellatus*), a common Mediterranean fish species, have priority of access to apparent parasite-rich breeding territories, and because they remain sedentary to defend their territories, exhibit higher prevalence of infection of the trematode worm, *Genitocotyle mediterranea*, than subordinate, non-territorial conspecifics^38^. Likewise, dominant reindeer (*Elaphostrongylus rangiferi*) consume more vegetation and consequently harbor more nematodes than subordinates^21^. Rank-associated differences in priority of access to mates can also lead to differences in parasite risk (e.g.^16–18^). For example, dominant male feral cats (*Felis catus*) have preferential access to females, and typically have higher incidence of feline immunodeficiency virus than subordinates^16^. The second driver, greater parasite susceptibility due to status-associated differences in mating effort, is based on the idea that, rather than investing in costly immune responses, high-ranking males, and sometimes females, allocate energy towards rank acquisition and mating effort, which in turn, increases susceptibility to parasitism^20,23^. In support, numerous studies have found tradeoffs between reproductive effort and immunity^39–41^. For instance, dominant male superb fairy wrens (*Malurus cyaneus*) exhibit iridescent blue plumage coloration and have higher testosterone levels than lower-ranking, duller males^42^. They also harbor significantly more blood-borne parasites than their subordinate counterparts^43^. Likewise, dominant, cooperatively breeding female meerkats exert greater mating effort than subordinate same-sex conspecifics, and this behavior is positively correlated with parasitism^26^. Thus, the behaviors of high-ranking individuals, namely outcompeting conspecifics for access to food, water, and social partners, and the exertion of mating effort, appear to be important divers of rank-associated differences in parasitism.

However, this overall pattern – higher parasitism in higher-ranking individuals – was more pronounced in analyses of males than females. Across male studies, high-ranking individuals harbored significantly more parasites than low-ranking individuals, while in females; this pattern was just a trend. These results suggest that key differences between the sexes in immunity, life history strategies, and behavior may interact with status-related differences in parasite exposure and susceptibility to create sex differences in status-related parasite risk. In support, the sex-related differences in status-based parasitism that we found are consistent with life history theory, which predicts that females will invest more in immune defense than males^44^. This theory is predicated on the idea that male fitness is limited by the number of receptive females and that female fitness is limited by offspring production^45^. Thus, females are expected to invest more in immune defense to promote longevity and offspring survival, whereas males should exert effort towards intrasexual competition at the expense of immune defense^44^. In agreement, males tend to have weaker immunity than females and suffer disproportionately from parasitism and disease^46,47^. These differences may be mediated by rank-driven, testosterone-induced immunosuppression^27,48^ (but see^28,29^), or alternatively, by sex-related differences in immunosuppressive stress hormones^49^. Additionally, rank-related differences in reproductive effort are probably weaker in females than males^37,44^; hence tradeoffs between reproductive effort and defense against parasites might not be as strong in females. For example, contrary to the predictions of the tradeoffs hypothesis, there were no significant rank-related differences among females in nepotistic or age-based linear hierarchies. Because rank relationships in nepotistic and age-based hierarchies tend to be stable across generations^50,51^, perhaps we should not expect to observe the same results as we found in male despotic hierarchies, where individuals need to fight to attain and maintain dominance rank (e.g.^52^). Lastly, in terms of priority of access to resources, in many mammalian societies, males outrank females^2^, thus high female rank doesn’t necessarily translate to high access to resources. For example, in chimpanzees, nearly all males outrank females, and skew in rank differences predicts whether food resources are transferred from low-ranking to high-ranking conspecifics^53^. In females, we found only partial support for the priority-of-access hypothesis, supporting the idea that differences in parasite exposure between high- and low-ranking conspecifics might be more prominent in one sex than the other.

The links between subordinate social status, allostatic load, and poor health are well-supported in the literature^1,2,12^. Classic studies of rodents^54,55^, non-human primates^56,57^, and humans^58^ have established a link between social subordination and hypercortisolism, inflammation, and chronic disease risk. Contrary to these patterns, our analyses of social status and parasitism did not support the stress-response, condition-dependent, or allostatic load hypotheses. Thus, rank-associated patterns of parasitism may differ from rank-associated patterns of many measures of health and immunity raising the question: why is parasitism different from other measures of health?

One explanation for why rank-associated patterns of parasitism might differ from rank-associated differences in health is that subordinate social stress does not alter immune responses relevant to parasite risk. A recent meta-analysis of social status and immune response found that subordinate males exhibited stronger antibody responses to antigens than dominants^3^. Antibodies are Th-2 mediated and a primary mechanism for fighting extracellular parasites^59^. Thus, the stronger response in low-ranking males might be indicative of resistance to gastrointestinal helminth infections^60^. In the same study^3^, dominant males exhibited significantly stronger delayed-type hypersensitivity reactions than subordinates, reflecting Th-1 mediated immunity and defense against intracellular parasites^61^. Because Th-1 responses can down-regulate Th-2 responses and vice versa^59^, subordinates may have stronger immune defenses against extracellular parasites than dominants. Indeed, the down-regulation of Th-2 mediated defenses in dominant individuals to increase mating effort is consistent with the tradeoffs hypothesis^20^. Alternatively, low-ranking males might be less stressed or in better condition than hypothesized^49^, which may explain why we did not find support for the stress-response or condition-dependent hypotheses. A recent review of social status and glucocorticoid levels across male and female vertebrates yielded mixed results, with the majority of studies reporting no significant differences in levels of stress hormones between high- and low-ranking conspecifics^7^. Similarly, in cooperative breeders, only one of 12 species exhibited higher glucocorticoids in subordinates than dominants^13^, indicating that chronic stress is not consistently associated with subordinate social status.

An alternate explanation for why patterns of parasitism are different than patterns of chronic disease risk is that many studies testing the relationship between social status and immunity occur in captive settings, and hierarchies in captivity may differ in important ways from hierarchies in the wild^62^ (although our findings do not support this idea; see below). Subordinate animals in captivity, unlike those in the wild, are incapable of avoiding dominant individuals, and may experience considerable chronic stress stemming from higher rates of received physical and psychological aggression than in the wild^5,13^. For example, dominant wolves (*Canis lupus*) exhibit higher levels of stress hormones in the wild^63^, whereas subordinates are more stressed in captivity^64^, suggesting that susceptibility to parasitism might differ based on study setting. While recent studies of captive animals have reported a link between subordinate social status and immunosuppression (e.g.^7,55^), whether such patterns persist in natural settings remains an open question. Indeed, a number of studies have reported differences in immune response in captive versus wild animals again suggesting that study setting matters (e.g.^65–67^). In the present study, the inclusion of study setting rarely improved model fit. This might be due to the paucity of captive studies. For example, over 90% of the male analyses occurred in wild settings with no provisioning, while the remainder occurred in captivity. Further, although female study settings included both non-provisioned (66%) and provisioned (32%) wild populations, only one analysis (2%) occurred in captivity. Thus, the lack of captive studies on social status and parasitism might explain why study setting did not significantly improve model fit for most of our analyses.

We found that relative allostatic load was not correlated with relative parasitism. This result was interesting and somewhat surprising because the allostatic load model integrates ideas from the other hypotheses proposed in this study – i.e. the costs of attaining and maintaining dominance rank (tradeoffs hypothesis), the costs of subordinate social status (stress response hypothesis), and differential access to resources (condition-dependent hypothesis). A well-cited study by Goymann and Wingfield^32^ found a significant correlation between relative allostatic load and relative cortisol, a marker of chronic stress. In our study, we replaced relative cortisol with relative parasitism, but we found no correlation between relative parasitism and relative allostatic load. Our results suggest the possibility that relative allostatic load plays a less palpable role in modulating parasitism than it does in modulating stress. If so, our results refute an established paradigm – the idea that the accumulation of physiological burdens decreases immune defense and increases susceptibility to parasitism^1,12,49^. Alternatively, there might be limitations in the metric used to quantify allostatic load^32^ as it is largely qualitative. Protocols for measuring allostatic load quantitatively have been well-established for assessing human subjects^31^. Analogous protocols for assessing allostatic load in free-ranging vertebrates that are uniform, practical, and measurable would be an important step for solving this problem.

Our findings suggest several important areas of subsequent investigation. First, we recommend that researchers work to increase the taxonomic diversity of both vertebrates and parasites in future studies. While we identified many studies that compared social status and parasitism in male and female vertebrates, like most meta-analyses, we were limited by the available data, and some taxonomic groups were better represented than others. Studies of egalitarian hierarchies, monogamous mating systems, and vector-borne parasites were relatively uncommon. Thus, diverse mating systems, hierarchy types, and parasite transmission modes are needed to better distinguish competing hypotheses. Moreover, most of the parasites included in our analyses consisted of helminths and other eukaryotic parasites. Thus, an important area for future work will be to test these hypotheses for non-eukaryote parasites (i.e. viruses and bacteria).

To better distinguish competing hypotheses, we also recommend that future studies of social status and parasitism expand the battery of tests applied to individual species. Two examples of this approach have been applied in Grant’s gazelles (*Nanger granti*)^22,68^. First, Ezenwa^22^ completed two steps to evaluate the priority-of-access hypothesis: (i) quantifying the density of parasites in the habitats of dominant, territorial Grant’s gazelle males and the density of parasites in the habitats of subordinate, non-territorial, males, and (ii) comparing rates of parasitism in dominants versus subordinates. This approach was especially useful because it not only quantified differences in parasitism between dominants and subordinates, but also established actual rank-related differences in exposure. Second, more recently, Ezenwa and colleagues^68^ used multiple measurements to assess a variant of the tradeoffs hypothesis (i.e. ICHH), which included measures of parasitism, horn size (a proxy for condition), testosterone, social status, and quantification of innate and adaptive immune responses. Thus, as illustrated by the above examples, future research needs to move beyond the simple assessment of social status and parasitism, to experimental work and study designs that incorporate comprehensive approaches for distinguishing alternative hypotheses to explain status-related variation in parasitism.

## Conclusions

Here we considered the importance of both parasite exposure and susceptibility in explaining the link between social status and parasitism in vertebrates. By pooling the estimated effect sizes from multiple studies, we increased our statistical power, which helped us to discern competing hypotheses proposed to explain status-related variation in parasitism. The application of multi-level meta-analytic models allowed us to incorporate standardized effect sizes weighted by sample size, and to control for differences in phylogeny, species, and study, thus providing an accurate estimate of how variation in social status is linked to parasitism. Based on available studies, our data supported the priority of access hypothesis and the tradeoffs hypothesis suggesting that both exposure to parasites and mating effort might be important contributors to rank-associated differences in parasitism. Strikingly, in contrast to hypotheses that predict higher parasitism in low-ranking animals, we found that high-ranking males exhibited significantly higher parasitism than low-ranking males and a non-significant trend in which high-ranking females exhibited greater parasitism than low-ranking females. Our study was an important step for synthesizing the existing literature, for deciphering the mechanisms that drive status-related variation in vertebrates, and for providing a framework for future research.

## Methods

### Identification of studies and inclusion criteria

We searched for published studies that compared social status and parasitism in male and female vertebrates using ISI Web of Science and Google Scholar. We followed the protocols for Preferred Reporting in Systematic Reviews and Meta-Analyses (PRISMA)^69^ (Fig. S7). For both search engines, in the advanced settings, we set the timespan from 1900–2016 and allowed all languages. We used the basic search function (topic in Web of Science) and all possible combinations of the following two sets of search terms: (i) social status; social domin*; social hierarchy; rank, and (ii) parasit*; disease. We also examined the reference lists in each study to identify studies not found in the databases. We included studies that compared the association between social status and parasitism in adult male and/or female vertebrates, and that reported effect sizes and sample sizes. Some authors only reported effect sizes (e.g. means and standard errors, means and standard deviations, etc.) for their significant results, but did not report effect sizes for their non-significant results. In these and other cases where sample sizes or effect sizes were not reported, we attempted to obtain these data in three ways: (i) by contacting the study authors via email, (ii) extracting effects sizes from figures using WebPlotDigitzer 2.0^70^, and (iii) calculating effect sizes using data published in online repositories (i.e. using the dataset published along with a given paper). We included studies that occurred in natural, provisioned, or captive settings. We excluded analyses that did not directly compare dominants to subordinates, studies of juveniles, and studies of castrated males. For the purposes of this study, we used the following conceptual definition of social status: any dominance relationship in which one individual is consistently outcompeted by another in a dyadic agonistic interaction^71^. For clarity, the terms (i) high-ranking and dominant and (ii) low-ranking and subordinate were used interchangeably^3^.

### Data extraction

We extracted the following information from each study: (i) study species, (ii) study setting as captive (laboratory or zoo animals) or wild (provisioned or non-provisioned), (iii) the method of measuring dominance rank (Table S4), (iv) the type of dominance hierarchy and mating system (Table S9), (v) the method of measuring parasitism (e.g. prevalence, infection intensity, or parasite species richness), (vi) parasite taxa and transmission modes (Table S10), (vii) information for calculating allostatic load scores for dominant and subordinate individuals (Table S11), and (viii) effect sizes. Whenever possible, we extracted the type of dominance hierarchy, mating system, and parasite transmission mode from the paper in question. In cases in which this information was not available, we extracted these data from alternative reference sources.

#### Measuring allostatic load

We calculated an allostatic load score for dominant and subordinate males and females using the methods outlined in Goymann and Wingfield^32^. To do so, we directly contacted the authors of each study (Table S11) and asked them to complete the selection criteria used to quantify allostatic load. If these author(s) did not respond to our request, we (BH, MD, KW, JO) searched for additional references (preferably from the same population), and each of us independently assigned an allostatic load score for dominant and subordinate males and females. In such cases, the median value was used from our analyses. A summary of how allostatic load scores were calculated is provided in Table S12.

#### Phylogenetic tree construction

To control for phylogenetic signal, we constructed two separate phylogenies based on relationships in the Tree of Life for the 21 male (Fig. S3) and ten female host species (Fig. S5) included in our meta-analyses^72^. We refined the topology and extracted divergence times from additional primary literature (Table S13). We built ultrametric trees using the R package *ape* (version 3.4^73^,) and adjusted node ages for published divergence times using BLADJ in Phylocom 4.2^74^.

#### Statistical analyses

To test the association between social status and parasitism, we applied a meta-analytic approach. Males and females were analyzed separately. For each analysis, we designated subordinates as the control, and assigned dominants as the treatment. In some studies, researchers divided animals into two distinct groups – those who were high-ranking and those who were low-ranking as defined by the study, and then reported the mean and standard error or mean and standard deviation for both groups. In other studies, the researchers assigned each individual an ordinal rank, and then correlated ordinal rank with parasitism. In these and other cases, we used an effect size calculator^75^ to convert means and standard errors, means and standard deviations, and other statistical measures into a standardized mean difference, Cohen’s *d*. We evaluated significance by determining the 95% confidence intervals surrounding *d*. Negative values indicated studies in which dominant individuals exhibited lower parasitism than subordinates. Positive values reflected studies in which subordinate individuals exhibited lower parasitism than dominants.

We performed phylogenetic meta-analyses using the *brms* package^36^ in R. Because of the multifactorial nature of drivers of parasitism, we used multi-level models, which included three random effects: (i) *study identity*, to control for non-independence among data points from the same study; (ii) *species*, to account for non-phylogenetic variation among species; and (iii) *phylogeny*, to account for phylogenetic relatedness among species. We incorporated effect size weights by linking the sampling variances with their estimated effect sizes using the se function within each model’s response term^36^. The model with the lowest k-fold information criterion (kfoldIC) was selected after performing k-fold cross validation in the *brms* package, with k equal to the maximum of ten or n (number of effect sizes)^36^. We included *study setting* as a fixed effect in all analyses where doing so improved statistical support for the model, based on k-fold cross validation. When study setting did not improve the model, we excluded this moderator from the final analysis. For males, there were no studies that occurred in wild settings with provisioning; therefore, we only included wild (without provisioning) and captive studies as fixed effects. For females, we included wild (with and without provisioning) and captive studies as fixed effects. For all models, we specified non-informative normal priors (µ = 0 and σ = 10) for the fixed effects and non-informative Student’s t priors (df = 3, µ = 0 and σ = 10) for the random-effects. For each model, four chains were run in parallel for at least 4000 iterations, with a warm-up period of at least 1000 iterations; convergence was confirmed with Gelman–Rubin diagnostics^76^, showing all potential scale reduction factors < 1.1. For each model, we report the effect size (intercept) as posterior mean, two-tailed 95% credible interval (CI), and one-tailed percent of posterior samples (p) less than zero for a positive mean effect size or greater than zero for a negative mean effect size. We quantified total heterogeneity using the I^2^ statistic^77,78^. We tested for publication bias using both visual (funnel plot) and quantitative (Egger’s test) assessments^79,80^. When an Egger’s test revealed significant publication bias, we used the trim and fill method to estimate the number of missing studies^79,80^.

To examine clade-specific variation in status-based parasitism, we performed meta-analyses on subsets of the phylogenetic tree. Specifically, we evaluated the following taxa for males: (i) Aves; (ii) Carnivora + Artiodactyla + Perissodactyla; (iii) Cercopithecidae; (iv) Diapsida; (v) Mammalia; (vi) Primates; and (vii) Squamata. We evaluated the following taxa for females: (i) Carnivora + Artiodactyla + Perissodactyla; (ii) *Papio*; (iii) *Papio* + *Mandrillus* + *Macaca*; and (iv) Primates. To examine variation based on the metric used to measure parasitism, we conducted analyses (separately for each sex) on subsets of parasitological measures. Specifically, we tested the association between social status and (i) richness; (ii) intensity; and (iii) prevalence.

We tested predictions of the tradeoffs, stress-response, priority-of-access, and condition-dependent hypotheses by comparing status-related variation based on hierarchy type, mating system, and parasite transmission mode. As before, we incorporated multi-level meta-analytic models and tests for significance in the *brms* package^36^. For these sub-analyses, we conducted meta-analyses for sample sizes of six or more, and pruned each phylogenetic tree accordingly.

#### Analyses of allostatic load

To test the allostatic load hypothesis, we applied Pearson product-moment correlations to compare relative allostatic load to relative parasitism. Relative parasitism was calculated by dividing mean parasitism in dominants by mean parasitism in subordinates for studies where these data were available. Relative allostatic load was calculated by dividing dominant allostatic load by subordinate allostatic load. As in prior analyses, males and females were assessed separately.

### Data availability

Analyses reported in this article can be reproduced using the sample code provided in the supplement (see supplementary materials). Data used for meta-analyses is available in the supplement, and has been uploaded to the Github repository: https://github.com/mdoellma/social_status_parasitism_meta_analysis.

## Electronic supplementary material

Supplementary Materials

Supplementary Code
