# Supplementary material for: Social status and parasitism in male and female vertebrates: a meta-analysis
Source: Sci Rep. 2018 Feb 26;8:3629. doi: 10.1038/s41598-018-21994-7 (PMC5827031; doi:10.1038/s41598-018-21994-7)
Supplement: Supplementary file 1 — Supplementary Materials [file 41598_2018_21994_MOESM1_ESM.docx]

**Supplementary materials for**

**Social status and parasitism in male and female vertebrates: a meta-analysis**

**Bobby Habig^*^, Meredith M. Doellman, Kourtney Woods, Jonathan Olansen, and Elizabeth A. Archie**

*Department of Biological Sciences, University of Notre Dame, 100 Galvin Life Sciences Center, Notre Dame, IN 46556, USA*

**Corresponding author; e-mail:* rhabig@nd.edu

**Table S1.** Predictions of the tradeoffs, stress-response, priority-of-access, and condition-dependent hypotheses; D = dominant individuals; S= subordinate individuals. For definitions of hierarchy types, mating systems, and parasite transmission modes, please refer to Table S2.

|  | **condition-dependent** | **stress-response** | **priority-of-access** | **tradeoffs** |
| --- | --- | --- | --- | --- |
| **hierarchy type** |  |  |  |  |
| linear (despotic, age-based and nepotistic) | **D<S** | **D<S** | **D>S** | **D>S** |
| egalitarian | **D=S** | **D=S** | **D=S** | **D=S** |
| **mating system** |  |  |  |  |
| monogamy | **D=S** | **D=S** | **D=S** | **D=S** |
| polygynandry | **D<S** | **D<S** | **D>S** | **D>S** |
| polyandry | **D<S (females only)** | **D<S (females only)** | **D>S (females only)** | **D>S (females only)** |
| polygyny | **D<S (males only)** | **D<S (males only)** | **D>S (males only)** | **D>S (males only)** |
| cooperative breeding | **D<S** | **D<S** | **D>S** | **D>S** |
| **parasite transmission mode** |  |  |  |  |
| environmentally- and contact-transmitted | **D<S** | **D<S** | **D>S** | **D>S** |
| vector-borne | **D<S** | **D<S** | **D=S** | **D>S** |

**Table S2.** Description of common definitions of dominance hierarchy structures (adapted from Archie ^1^) and mating systems (adapted from Fuentes ^2^) of vertebrates and parasite transmission modes of parasites.

| **type of hierarchy** | **description** |
| --- | --- |
| **linear despotic** | Resource access is highly skewed in favor of dominant individuals. Characterized by high levels of aggression and intimidation. |
| **egalitarian** | Resources are distributed equally and dominance is attained and maintained through social support. |
| **nepotistic** | Classification of despotic or egalitarian hierarchies. Maternal kin commonly assist each other to attain resources and maintain rank (with relatives obtaining similar rank). |
| **age-based** | Classification of despotic or egalitarian hierarchies. Rank is determined by order of birth such that the oldest individual is the most dominant. |
| **type of mating system** | **description** |
| **monogamous** | A single male mates exclusively with one female during a mating period, and that female mates exclusively with that male during the mating period |
| **polyandrous** | A single female mates with multiple males during a mating period |
| **polygynous** | A single male mates with multiple females during a mating period |
| **polygynandrous** | Males and females mate with multiple partners within a mating period |
| **cooperative breeding** | Mating is skewed, and occurs in a breeding pair (monogamy) or in multiple males and females (polygynandry); non-mating individuals provide alloparental care. |
| **mode of transmission** | **description** |
| **environmentally transmitted** | Parasites transmitted through exposure to contaminated environments (e.g. polluted water or fecal-oral transmission). |
| **contact transmitted** | Parasites transmitted by direct, physical contact between conspecifics hosts (e.g. sexually-transmitted infections). |
| **vector-borne** | Parasites transmitted via an intermediate host (vector), e.g. mosquito-borne parasites. In this study, the vector-borne parasites that we analyzed were exclusively flying insects. |

**Table S3.** Number and percentage of taxonomic groups represented across male and female studies of social status and parasitism.

| **taxonomic groups (male studies)** | |
| --- | --- |
| Order Primates | 36% (n=24) |
| Order Carnivora | 27% (n=18) |
| Class Aves | 12% (n=8) |
| Order Perissodactyla | 12% (n=8) |
| Class Actinopterygii | 5% (n=3) |
| Order Squamata | 5% (n=3) |
| Order Artiodactyla | 3% (n=2) |
| **taxonomic groups (female studies)** | |
| Order Primates | 58% (n=36) |
| Order Carnivora | 27% (n=17) |
| Order Perissodactyla | 13% (n=8) |
| Order Artiodactyla | 2% (n=1) |

**Table S4.** Methods used to measure dominance in the 26 male studies and 13 female studies included in our meta-analyses.

| **methods of measuring dominance** | | |
| --- | --- | --- |
| **male studies** | | |
| ***behavior*** | 22 | |
| outcomes of agonistic interactions | 16 | |
| territory holding ability | 3 | |
| mating behavior | 2 | |
| scent marking | 1 | |
|  |  | |
| ***morphology*** | 3 | |
| coloration | 3 | |
|  |  | |
| ***demography*** | 1 | |
| age | 1 | |
| **female studies** | | |
| ***behavior*** | | 12 |
| outcomes of agonistic interactions | | 10 |
| avoidance behavior | | 1 |
| scent marking | | 1 |
|  | |  |
| ***demography*** | | 1 |
| age | | 1 |

**Table S5.** Male meta-analyses incorporating three random-effects: (1) study identity (model 1); (2) study identity (model 2) and species; and (3) study identity, species, and phylogeny (model 3). Best supported models, based on kfoldIC are highlighted in gray. Variance estimates are reported as standard deviations of the random effects.

| **type of meta-analysis** | **sample size (analyses)** | **model ^A, B^** | **kfoldIC** | **standard difference in means** | **95% HPD lower limit** | **95% HPD upper limit** | | ***p*** | | **variance estimate** | | | **total**  **heterogeneity (I^2^)**  **with credible intervals** | **higher in dominant or subordinate** |
| --- | --- | --- | --- | --- | --- | --- | --- | --- | --- | --- | --- | --- | --- | --- |
|  |  |  |  |  |  |  |  |  |  | **study** | **species** | **phylogeny** |  |  |
| **male studies** | | | | | | | | | | | | | | |
| **all male studies** | 66 | model 1 | 152.204 | 0.511 | 0.130 | | 0.974 | | 0.005 | 0.875 | – | – | 99.19 (98.46-99.77) | dominant |
|  |  | model 2 | 157.839 | 0.466 | 0.024 | | 0.955 | | 0.020 | 0.639 | 0.563 | – | 99.27 (98.59-99.80) |  |
|  |  | model 3 | 158.699 | 0.493 | -0.492 | | 1.538 | | 0.122 | 0.640 | 0.532 | 0.599 | 99.47 (98.93-99.91) |  |
| **males in despotic hierarchies** | 59 | model 1 | 147.138 | 0.542 | 0.127 | | 1.018 | | 0.006 | 0.928 | – | – | 99.34 (98.74-99.82) | dominant |
|  |  | model 2 | 140.225 | 0.493 | 0.013 | | 1.001 | | 0.023 | 0.677 | 0.600 | – | 99.40 (98.83-99.82) |  |
|  |  | model 3 | 140.553 | 0.498 | -0.605 | | 1.663 | | 0.132 | 0.674 | 0.566 | 0.647 | 99.57 (99.10-99.92) |  |
| **males in egalitarian hierarchies** | 6 | NA ^C^ | 12.112 | 0.170 | -0.532 | | 0.902 | | 0.300 | NA ^C^ | NA ^C^ | NA ^C^ | 24.06 (0.01-78.97) | neither |
| **males in polygynandrous mating systems** | 29 | model 1 | 79.085 | 0.943 | 0.348 | | 1.671 | | 0.001 | 0.938 | – | – | 78.29 (59.92-95.22) | dominant |
|  |  | model 2 | 80.827 | 0.924 | 0.197 | | 1.795 | | 0.008 | 0.874 | 0.562 | – | 82.20 (64.09-96.48) |  |
|  |  | model 3 | 80.944 | 0.929 | -0.003 | | 2.775 | | 0.010 | 0.931 | 0.608 | 0.924 | 88.37 (73.61-99.72) |  |
| **males in cooperative breeding mating systems** | 18 | model 1 | 29.307 | 0.390 | -1.573 | | 2.344 | | 0.161 | 1.098 | – | – | 71.27 (27.28-99.97) | neither |
| **males in polygynous mating systems** | 15 | model 1 | 41.34 | -0.168 | -1.272 | | 1.114 | | 0.322 | 1.207 | – | – | 94.42 (83.25-99.93) | neither |
|  |  | model 2 | 41.34 | -0.095 | -1.688 | | 1.638 | | 0.421 | 1.133 | 1.137 | – | 96.99 (99.19-99.96) |  |
|  |  | model 3 | 41.91 | -0.106 | -3.088 | | 2.933 | | 0.448 | 1.299 | 1.262 | 1.536 | 98.50 (95.45-99.98) |  |
| **males in monogamous mating systems** | 4 | model 1 | 2.787 | 0.229 | -6.677 | | 6.209 | | 0.355 | 3.111 | – | – | 84.91 (28.92-99.99) | neither |
| **environmentally- and contact-transmitted parasites** | 53 | model 1 | 122.344 | 0.704 | 0.238 | | 1.276 | | 0.002 | 0.960 | – | – | 99.44 (98.91-99.85) | dominant |
|  |  | model 2 | 126.257 | 0.678 | 0.116 | | 1.306 | | 0.012 | 0.768 | 0.601 | – | 99.52 (99.04-99.89) |  |
|  |  | model 3 | 127.222 | 0.674 | -0.630 | | 2.033 | | 0.107 | 0.767 | 0.562 | 0.745 | 99.66 (99.27-99.96) |  |
| **males exposed to parasites transmitted by flying-vectors** | 6 | model 1 | 17.003 | -0.024 | -2.334 | | 2.382 | | 0.487 | 1.740 | – | – | 90.45 (70.47-99.96) | neither |
|  |  | model 2 | 15.507 | -0.104 | -4.244 | | 3.914 | | 0.469 | 2.018 | 2.046 | – | 95.29 (83.32-99.99) |  |
|  |  | model 3 | 15.889 | -0.064 | -7.136 | | 7.102 | | 0.481 | 2.628 | 2.626 | 3.228 | 98.09 (92.60-99.99) |  |
| **parasite richness** | 9 | model 1 | 18.992 | 0.498 | -0.103 | | 1.102 | | 0.043 | 0.387 | – | – | 58.72 (15.90-96.81) | dominant |
|  |  | model 2 | 19.928 | 0.469 | -0.418 | | 1.355 | | 0.103 | 0.421 | 0.503 | – | 71.16 (33.19-99.49) |  |
|  |  | model 3 | 21.464 | 0.379 | -2.252 | | 2.743 | | 0.267 | 0.516 | 0.650 | 1.122 | 85.13 (55.39-99.94) |  |
| **parasite intensity** | 30 | model 1 | 84.974 | 0.590 | 0.021 | | 1.316 | | 0.022 | 1.028 | – | – | 85.49 (70.84-96.78) | dominant |
|  |  | model 2 | 86.840 | 0.572 | -0.092 | | 1.378 | | 0.044 | 0.932 | 0.597 | – | 87.93 (74.86-97.59) |  |
|  |  | model 3 | 89.524 | 0.514 | -0.942 | | 1.974 | | 0.171 | 0.964 | 0.602 | 0.765 | 91.39 (80.83-99.07) |  |
| **parasite prevalence ^B^** | 26 | model 1 | 54.997 | 1.497 | 0.358 | | 2.586 | | 0.010 | 0.465 | – | – | 98.36 (95.81-99.94) | neither |
|  |  | model 2 | 58.333 | 1.507 | 0.160 | | 2.924 | | 0.017 | 0.379 | 0.510 | – | 99.24 (98.03-99.98) |  |
|  |  | model 3 | 54.977 | 1.537 | -0.468 | | 3.601 | | 0.049 | 0.371 | 0.511 | 0.805 | 99.66 (99.01-99.99) |  |
| **polygynous + polygynandrous ^B^** | 44 | model 1 | 118.983 | 2.047 | 0.508 | | 3.710 | | 0.005 | 0.997 | – | – | 91.93 (84.17-97.66) | dominant |
|  |  | model 2 | 120.803 | 2.004 | 0.339 | | 3.834 | | 0.011 | 0.822 | 0.589 | – | 92.81 (85.43-98.14) |  |
|  |  | model 3 | 116.295 | 1.950 | -0.164 | | 4.090 | | 0.033 | 0.807 | 0.539 | 0.778 | 94.86 (88.99-99.22) |  |
| **polygynandrous + cooperative breeding ^B^** | 47 | model 1 | 106.429 | 1.854 | 0.751 | | 3.102 | | 0.001 | 0.601 | – | – | 74.52 (47.65-95.11) | dominant |
|  |  | model 2 | 107.820 | 1.868 | 0.645 | | 3.304 | | 0.002 | 0.552 | 0.386 | – | 79.44 (56.70-96.32) |  |
|  |  | model 3 | 107.432 | 1.945 | 0.271 | | 3.811 | | 0.015 | 0.606 | 0.420 | 0.557 | 86.33 (67.63-99.28) |  |
| **monogamous + egalitarian** | 11 | model 1 | 12.921 | 0.270 | -1.265 | | 1.841 | | 0.207 | 0.813 | – | – | 53.07 (5.49-99.92) | neither |
| **polygynous + polygynandrous + cooperative breeding ^B^** | 62 | model 1 | 146.776 | 1.950 | 0.583 | | 3.453 | | 0.003 | 0.851 | – | – | 99.18 (98.29-99.81) | dominant |
|  |  | model 2 | 144.780 | 1.928 | 0.439 | | 3.533 | | 0.007 | 0.658 | 0.535 | – | 99.28 (98.52-99.85) |  |
|  |  | model 3 | 145.632 | 1.906 | 0.071 | | 3.798 | | 0.022 | 0.645 | 0.510 | 0.645 | 99.50 (98.89-99.95) |  |

**^A^** In cases in which models 2 and 3 and variance estimates were excluded, there were insufficient variation in species and phylogeny available to conduct these analyses; **^B^** In these models, the addition of study setting (wild non-provisioned, captive) significantly improved model fit and was kept in the model. For all other models, study setting was removed from the final model because it did not significantly improve model fit; ^C^ NA = not applicable (All effect sizes for egalitarian hierarchies were from the same study and species).

**Table S6.** Clade-specific meta-analyses of male studies, incorporating three random-effects: (1) study identity (model 1); (2) study identity (model 2) and species; and (3) study identity, species, and phylogeny (model 3). Best supported models, based on kfoldIC are highlighted in gray. Variance estimates are reported as standard deviations of the random effects.

| **type of meta-analysis** | **sample size (analyses)** | **model ^A,B^** | **kfoldIC** | **standard difference in means** | **95% HPD lower limit** | **95% HPD upper limit** | | ***p*** | | **variance estimate** | | | **total**  **heterogeneity (I^2^)**  **with credible intervals** | **higher in dominant or subordinate** |
| --- | --- | --- | --- | --- | --- | --- | --- | --- | --- | --- | --- | --- | --- | --- |
|  |  |  |  |  |  |  |  |  |  | **study** | **species** | **phylogeny** |  |  |
| **male studies** | | | | | | | | | | | | | | |
| Diapsida | 11 | model 1 | 21.709 | -0.009 | -0.646 | | 0.584 | | 0.494 | 0.589 | – | – | 74.08 (38.96-97.80) | neither |
|  |  | model 2 | 22.122 | -0.023 | -0.806 | | 0.761 | | 0.475 | 0.546 | 0.549 | – | 82.12 (56.65-99.13) |  |
|  |  | model 3 | 21.799 | -0.045 | -1.803 | | 1.655 | | 0.472 | 0.546 | 0.551 | 0.901 | 89.81 (72.11-99.90) |  |
| Aves | 8 | model 1 | 9.753 | 0.140 | -0.574 | | 0.927 | | 0.310 | 0.549 | – | – | 67.84 (24.51-99.47) | neither |
| Squamata | 3 | model 1 | 12.400 | -0.434 | -7.788 | | 6.868 | | 0.401 | 3.288 | – | – | 96.52 (85.56-99.99) | neither |
| Mammalia | 52 | model 1 | 122.162 | 0.779 | 0.200 | | 1.523 | | 0.004 | 1.077 | – | – | 99.51 (98.93-99.91) | dominant |
|  |  | model 2 | 119.050 | 0.742 | 0.042 | | 1.569 | | 0.020 | 0.862 | 0.701 | – | 99.60 (99.10-99.94) |  |
|  |  | model 3 | 119.259 | 0.752 | -0.491 | | 2.108 | | 0.085 | 0.872 | 0.772 | 0.694 | 99.72 (99.36-99.98) |  |
| Carnivora + Artiodactyla + Perissodactyla | 28 | model 1 | 54.737 | 0.817 | -0.611 | | 2.533 | | 0.106 | 1.639 | – | – | 99.83 (99.53-99.99) | neither |
|  |  | model 2 | 51.917 | 0.964 | -1.276 | | 3.436 | | 0.146 | 0.988 | 1.835 | – | 99.90 (99.71-99.99) |  |
|  |  | model 3 | 52.350 | 0.966 | -3.222 | | 5.128 | | 0.237 | 1.014 | 2.016 | 2.178 | 99.95 (99.84-99.99) |  |
| Primates | 24 | model 1 | 65.260 | 0.826 | 0.043 | | 1.848 | | 0.020 | 1.151 | – | – | 77.93 (51.40-97.80) | dominant |
|  |  | model 2 | 65.399 | 0.750 | -0.446 | | 2.066 | | 0.084 | 1.096 | 0.833 | – | 83.99 (61.54-99.12) |  |
|  |  | model 3 | 66.151 | 0.703 | -2.564 | | 3.784 | | 0.234 | 1.227 | 1.031 | 1.469 | 91.22 (75.25-99.95) |  |
| Cercopithecidae (*Papio* + *Mandrillus* + *Macaca*) | 15 | model 1 | 42.054 | 1.090 | -0.380 | | 2.765 | | 0.059 | 1.805 | – | – | 88.33 (70.84-99.37) | neither |
|  |  | model 2 | 41.486 | 0.963 | -1.447 | | 3.449 | | 0.163 | 1.825 | 1.517 | – | 92.75 (80.37-99.84) |  |
|  |  | model 3 | 41.485 | 0.981 | -5.242 | | 7.050 | | 0.297 | 2.001 | 1.857 | 3.053 | 96.85 (89.86-99.97) |  |

**^A^** In cases in which models 2 and 3 and variance estimates were excluded, there were insufficient variation in species and phylogeny available to conduct these analyses; **^B^** In all models, study setting (wild non-provisioned, wild provisioned, captive) did not significantly improve model fit and was removed from all final models; **^B^** In all models, study setting (wild non-provisioned, captive) did not significantly improve model fit and was removed from all final models

**Table S7.** Female meta-analyses incorporating three random-effects: (1) study identity (model 1); (2) study identity (model 2) and species; and (3) study identity, species, and phylogeny (model 3). Best supported models, based on kfoldIC are highlighted in gray. Variance estimates are reported as standard deviations of the random effects.

| **type of meta-analysis** | **sample size (analyses)** | **model ^A,B^** | **kfoldIC** | **standard difference in means** | **95% CI lower limit** | **95% CI upper limit** | | ***p*** | | **variance estimate** | | | **total**  **heterogeneity (I^2^)**  **with credible intervals** | **higher in dominant or subordinate** |
| --- | --- | --- | --- | --- | --- | --- | --- | --- | --- | --- | --- | --- | --- | --- |
|  |  |  |  |  |  |  |  |  |  | **study** | **species** | **phylogeny** |  |  |
| **female studies** | | | | | | | | | | | | | | |
| **all female studies** | 62 | model 1 | 114.020 | 0.322 | -0.059 | | 0.737 | | 0.044 | 0.611 | – | – | 98.33 (96.56-99.68) | dominant |
|  |  | model 2 | 117.139 | 0.279 | -0.183 | | 0.751 | | 0.104 | 0.570 | 0.296 | – | 98.58 (97.01-99.76) |  |
|  |  | model 3 | 118.962 | 0.213 | -0.856 | | 1.220 | | 0.271 | 0.568 | 0.301 | 0.573 | 99.12 (97.92-99.97) |  |
| **females in despotic nepotistic hierarchies** | 44 | model 1 | 96.607 | 0.331 | -0.142 | | 0.852 | | 0.081 | 0.659 | – | – | 81.43 (62.40-96.66) | neither |
|  |  | model 2 | 92.644 | 0.272 | -0.331 | | 0.872 | | 0.153 | 0.618 | 0.361 | – | 84.47 (66.96-98.34) |  |
|  |  | model 3 | 92.562 | 0.168 | -2.127 | | 2.377 | | 0.365 | 0.616 | 0.355 | 1.070 | 92.16 (78.68-99.88) |  |
| **females in despotic age-based hierarchies** | 18 | model 1 | 23.405 | 0.324 | -2.549 | | 3.371 | | 0.309 | – | – | – | 90.16 (66.83-99.99) | neither |
| **females in polygynandrous mating systems** | 29 | model 1 | 66.568 | 0.491 | -0.093 | | 1.156 | | 0.045 | 0.731 | – | – | 64.77 (33.91-92.49) | neither |
|  |  | model 2 | 62.579 | 0.457 | -0.571 | | 1.447 | | 0.121 | 0.755 | 0.585 | – | 74.52 (46.91-98.92) |  |
|  |  | model 3 | 63.674 | 0.528 | -2.076 | | 3.216 | | 0.239 | 0.798 | 0.752 | 1.222 | 85.23 (60.24-99.94) |  |
| **females in cooperative breeding mating systems** | 17 | model 1 | 19.646 | 0.547 | -5.953 | | 6.788 | | 0.286 | 3.460 | – | – | 92.40 (67.09-99.99) | neither |
| **polygynandrous + cooperative breeding** | 46 | model 1 | 77.946 | 0.508 | 0.067 | | 1.004 | | 0.015 | 0.615 | – | – | 76.16 (55.39-95.24) | dominant |
|  |  | model 2 | 79.325 | 0.479 | -0.189 | | 1.147 | | 0.061 | 0.638 | 0.400 | – | 82.41 (63.29-98.12) |  |
|  |  | model 3 | 80.779 | 0.517 | -2.416 | | 3.472 | | 0.226 | 0.671 | 0.488 | 1.295 | 91.64 (76.05-99.98) |  |
| **females in polygynous mating systems** | 16 | model 1 | 32.001 | -0.266 | -1.488 | | 0.882 | | 0.166 | 0.646 | – | – | 74.78 (34.55-99.99) | neither |
| **females exposed to environmentally- and contact-transmitted parasites** | 61 | model 1 | 108.234 | 0.371 | -0.006 | | 0.808 | | 0.027 | 0.605 | – | – | 98.30 (96.41-99.67) | neither |
|  |  | model 2 | 108.553 | 0.338 | -0.209 | | 0.892 | | 0.087 | 0.561 | 0.385 | – | 98.70 (97.10-99.86) |  |
|  |  | model 3 | 108.569 | 0.315 | -1.201 | | 1.762 | | 0.244 | 0.572 | 0.419 | 0.745 | 99.27 (98.08-99.99) |  |
| **parasite richness** | 5 | model 1 | 17.576 | 1.382 | -1.507 | | 4.180 | | 0.091 | 1.643 | – | – | 89.99 (69.38-99.95) | neither |
|  |  | model 2 | 17.981 | 1.343 | -3.456 | | 6.088 | | 0.174 | 2.220 | 2.186 | – | 95.11 (81.67-99.98) |  |
|  |  | model 3 | 19.111 | 1.224 | -8.076 | | 9.852 | | 0.314 | 2.856 | 2.905 | 4.306 | 98.47 (93.98-99.99) |  |
| **parasite intensity** | 28 | model 1 | 59.500 | 0.115 | -0.221 | | 0.536 | | 0.266 | 0.399 | – | – | 55.41 (20.66-86.49) | neither |
|  |  | model 2 | 59.557 | 0.103 | -0.311 | | 0.563 | | 0.310 | 0.356 | 0.299 | – | 62.01 (28.90-90.46) |  |
|  |  | model 3 | 59.523 | 0.059 | -0.970 | | 1.081 | | 0.424 | 0.363 | 0.300 | 0.563 | 75.29 (45.89-98.67) |  |
| **parasite prevalence** | 29 | model 1 | 42.055 | 0.192 | -0.299 | | 0.707 | | 0.170 | 0.467 | – | – | 98.11 (94.99-99.98) | neither |
|  |  | model 2 | 47.223 | 0.159 | -0.624 | | 0.920 | | 0.282 | 0.503 | 0.453 | – | 98.99 (97.11-99.99) |  |
|  |  | model 3 | 47.900 | 0.167 | -2.174 | | 2.494 | | 0.375 | 0.637 | 0.688 | 1.025 | 99.57 (98.45-99.99) |  |

**^A^** In cases in which models 2 and 3 and variance estimates were excluded, there were insufficient variation in species and phylogeny available to conduct these analyses; **^B^** In all models, study setting (wild non-provisioned, wild provisioned, captive) did not significantly improve model fit and was removed from all final models; **^B^** In all models, study setting (wild non-provisioned, wild provisioned, captive) did not significantly improve model fit and was removed from all final models

**Table S8.** Clade-specific meta-analyses of female studies incorporating three random-effects: (1) study identity; (2) study identity and species; and (3) study identity, species, and phylogeny. Best supported models, based on kfoldIC are highlighted in gray. Variance estimates are reported as standard deviations of the random effects.

| **type of meta-analysis** | **sample size (analyses)** | **model ^A,B^** | **kfoldIC** | **standard difference in means** | **95% CI lower limit** | **95% CI upper limit** | | ***p*** | | **variance estimate** | | | **total**  **heterogeneity (I^2^)**  **with credible intervals** | **higher in dominant or subordinate** |
| --- | --- | --- | --- | --- | --- | --- | --- | --- | --- | --- | --- | --- | --- | --- |
|  |  |  |  |  |  |  |  |  |  | **study** | **species** | **phylogeny** |  |  |
| **female studies** | | | | | | | | | | | | | | |
| **Carnivora + Artiodactyla + Perissodactyla** | 26 | model 1 | 26.445 | 0.143 | -1.256 | | 1.559 | | 0.352 | 1.103 | – | – | 99.52 (98.56-99.99) | neither |
| **Primates** | 36 | model 1 | 85.470 | 0.390 | -0.110 | | 0.951 | | 0.055 | 0.645 | – | – | 62.53 (32.49-88.73) | neither |
|  |  | model 2 | 92.616 | 0.361 | -0.323 | | 1.091 | | 0.115 | 0.639 | 0.392 | – | 68.92 (40.68-93.80) |  |
|  |  | model 3 | 92.537 | 0.341 | -1.074 | | 1.705 | | 0.233 | 0.633 | 0.406 | 0.699 | 78.01 (53.02-99.18) |  |
| ***Papio* + *Mandrillus* + *Macaca*** | 29 | model 1 | 57.984 | 0.491 | -0.080 | | 1.138 | | 0.040 | 0.718 | – | – | 64.16 (33.55-92.99) | neither |
|  |  | model 2 | 61.722 | 0.450 | -0.536 | | 1.447 | | 0.128 | 0.742 | 0.594 | – | 74.39 (45.71-98.17) |  |
|  |  | model 3 | 70.401 | 0.511 | -2.238 | | 3.286 | | 0.242 | 0.809 | 0.781 | 1.265 | 85.69 (61.07-99.94) |  |
| ***Papio*** | 16 | model 1 | 32.523 | 0.360 | -0.566 | | 1.481 | | 0.171 | 0.888 | – | – | 69.88 (27.88-99.67) | dominant |
|  |  | model 2 | 30.786 | 0.310 | -3.049 | | 3.564 | | 0.334 | 1.255 | 1.668 | – | 86.93 (56.04-99.99) |  |

**^A^** In cases in which models 2 and 3 and variance estimates were excluded, there were insufficient variation in species and phylogeny available to conduct these analyses; **^B^** In all models, study setting (wild non-provisioned, wild provisioned, captive) did not significantly improve model fit and was removed from all final models.

**Table S9.** Types of dominance hierarchies and mating systems identified in 26 male studies of social status and parasitism and in 14 species/subspecies across 13 female studies of social status and parasitism.

| **study** | | **species** | **sample size** | | **type of dominance hierarchy** | **citation used to determine type of dominance hierarchy** | | **type of mating system** | | **citation used to determine type of mating system** | |
| --- | --- | --- | --- | --- | --- | --- | --- | --- | --- | --- | --- |
| **Male studies** | | | | | | | | | | | |
| **Akinyi et al. 2013** | *Papio cynocephalus* | | 24 | linear despotic | | ^3^ | | | polygynandrous | ^3^ | |
| **Arlet et al. 2015** | *Lophocebus albigena* | | 16 | linear despotic | | ^4^ | | | polygynandrous | ^4^ | |
| **Bartoli et al. 2000** | *Symphodus ocellatus* | | 117 | linear despotic | | ^5^ | | | polygynandrous | ^5^ | |
| **Borgia and Collis 1990** | *Ptilonorhynchus violaceus* | | 85 | linear despotic | | ^6^ | | | polygynous | ^6^ | |
| **Caine and Melfi 2006** | *Colobus guereza kikiyuensis* | | 6 | linear despotic | | ^7^ | | | polygynandrous | ^7^ | |
| **Clough et al. 2010** | *Eulemur fulvus rufus* | | 10-11 | egalitarian | | ^8^ | | | polygynandrous | ^8^ | |
| **Colombelli-Négrel and Kleindorfer 2008** | *Malurus cyaneus* | | 51 | linear despotic | | ^9^ | | | cooperative breeding | ^10^ | |
| **Dufva and Allander 1995** | *Parus major* | | 37 | linear despotic | | ^11^ | | | monogamous | ^11^ | |
| **Fugazzola and Stancampiano 2012** | *Equus quagga* | | 18 | linear despotic | | ^12^ | | | polygynous | ^12^ | |
| **Hausfater and Watson 1976** | *Papio anubis* | | 8 | linear despotic | | ^3^ | | | polygynandrous | ^3^ | |
| **Huyghe et al. 2009** | *Podacris melisellensis* | | 34 | linear despotic | | ^13^ | | | polygynandrous | ^13^ | |
| **Meade 1984** | *Papio cynocephalus* | | 8 | linear despotic | | ^3^ | | | polygynandrous | ^3^ | |
| **Melfi and Poyser 2007** | *Colobus guereza kikiyuensis* | | 6 | linear despotic | | ^14^ | | | polygynandrous | ^14^ | |
| **Mooring et al. 1996** | *Aepyceros melampu* | | 18 | linear despotic | | ^15^ | | | polygynandrous | ^16^ | |
| **Muehlenbein 2006** | *Pan troglodytes* | | 12 | linear despotic | | ^17^ | | | polygynandrous | ^17^ | |
| **Muehlenbein and Watts 2010** | *Pan troglodytes* | | 22 | linear despotic | | ^17^ | | | polygynandrous | ^17^ | |
| **Muller-Graf et al. 1996** | *Papio anubis* | | 27 | linear despotic | | ^3^ | | | polygynandrous | ^3^ | |
| **Negro et al. 2010** | *Arctocephalus forsteri* | | 12 | linear despotic | | ^18^ | | | polygynous | ^18^ | |
| **Pelletier et al. 2005** | *Ovis canadensis* | | 51 | linear despotic | | ^19^ | | | polygynandrous | ^20^ | |
| **Poiani et al. 2000** | *Passer domesticus* | | 28 | linear despotic | | ^21^ | | | monogamous | ^21^ | |
| **Schall and Houle 1992** | *Sceloporus occidentalis* | | 26 | linear despotic | | ^22^ | | | polygynous | ^23^ | |
| **Schall and Staats 2002** | *Anolis sabanus* | | 32 | linear despotic | | ^24^ | | | polygynous | ^25^ | |
| **Smyth and Drea 2015** | *Suricata suricatta* | | 38 | linear despotic | | ^26^ | | | cooperative breeding | ^26^ | |
| **Teichroeb et al. 2009** | *Colobus vellerosus* | | 14 | linear despotic | | ^27^ | | | polygynous | ^28^ | |
| **Leclaire and Faulkner 2014** | *Suricata suricatta* | | 22 | linear despotic | | ^29^ | | | cooperative breeding | ^29^ | |
| **Weatherhead et al. 1993** | *Agelaius phoeniceus* | | 45 | linear despotic | | ^30^ | | | polygynous | ^30^ | |
| **Female studies** | | | | | | | | | | | |
| **Akinyi et al. 2013** | *Papio cynocephalus* | | 38 | linear nepotistic | | | ^3^ | | polygynandrous | | ^3^ |
| **Foerster et al. 2015** | *Cercopithecus mitis albogularis* | | 11 | linear nepotistic | | | ^31^ | | polygynous | | ^31^ |
| **Foerster et al. 2015** | *Cercopithecus mitis stuhlmanni* | | 7-10 | linear nepotistic | | | ^31^ | | polygynous | | ^31^ |
| **Fugazzola and Stancampiano 2012** | *Equus quagga* | | 70 | linear nepotistic | | | ^12^ | | polygynous | | ^12^ |
| **Hausfater and Watson 1976** | *Papio cynocephalus* | | 29 | linear nepotistic | | | ^3^ | | polygynandrous | | ^3^ |
| **Hernandez et al. 2009** | *Macaca fuscata yakui* | | 13 | linear nepotistic | | | ^32^ | | polygynandrous | | ^3^ |
| **Leclaire and Faulkner 2014 J** | *Suricata suricatta* | | 12 | linear nepotistic | | | ^26^ | | polygynandrous | | ^29^ |
| **MacIntosh et al. 2012** | *Macaca fuscata yakui* | | 10 | linear nepotistic | | | ^32^ | | polygynandrous | | ^3^ |
| **Meade 1984** | *Papio cynocephalus* | | 63 | linear nepotistic | | | ^3^ | | polygynandrous | | ^3^ |
| **Müller-Graf, Collins, and Woolhouse 1996** | *Papio anubis* | | 12 | linear nepotistic | | | ^3^ | | polygynandrous | | ^3^ |
| **Ravasi 2009** | *Papio ursinus* | | 11 | linear nepotistic | | | ^3^ | | polygynandrous | | ^3^ |
| **Setchell et al. 2007** | *Mandrillus Sphinx* | | 35 | linear age-based | | | ^33^ | | cooperative breeding | | ^3^ |
| **Smyth and Drea 2015** | *Suricata suricatta* | | 27 | linear age-based | | | ^26^ | | polygynous | | ^26^ |
| **Ungerfeld and Correa 2007** | *Capra aegagrus* | | 32 | linear age-based | | | ^34^ | | polygynous | | ^34^ |

**Table S10.** Parasite taxa studied and modes of transmission identified in 26 male studies and 13 female studies of social status and parasitism. Note: In studies where multiple analyses were conducted, letters following authors’ names (i.e. A, B, C, etc.) were used to indicate an individual analysis.

| study | host species | sample size | | parasite species | mode of transmission | citation used to determine mode of transmission |
| --- | --- | --- | --- | --- | --- | --- |
| **male studies** | | | | | | |
| **Akinyi et al. 2013 A, B** | *Papio cynocephalus* | 24 | | *Rhipicephalus simus* | contact and environmental | ^35^ |
| **Akinyi et al. 2013 A, B** | *Papio cynocephalus* | 24 | | *Rhipicephalus pulchellus* | contact and environmental | ^35^ |
| **Akinyi et al. 2013 A, B** | *Papio cynocephalus* | 24 | | *Hyalomma truncatum* | contact and environmental | ^35^ |
| **Arlet et al. 2015 A, B, C** | *Lophocebus albigena* | 16 | | *Physaloptera* | environmental (non-biting vector) | ^36^ |
| **Arlet et al. 2015 A, B, D** | *Lophocebus albigena* | 16 | | *Trichuris* | environmental | ^36^ |
| **Arlet et al. 2015 A, B, F** | *Lophocebus albigena* | 16 | | *Strongyloidea* | environmental | ^36^ |
| **Bartoli et al. 2000 A** | *Symphodus ocellatus* | 117 | | *Lecithester stellatus* | environmental | ^5^ |
| **Bartoli et al. 2000 B** | *Symphodus ocellatus* | 117 | | *Macvicaria alacris* | environmental | ^5^ |
| **Bartoli et al. 2000 C** | *Symphodus ocellatus* | 117 | | *Genitocotyle mediterranea* | environmental | ^5^ |
| **Borgia and Collis 1990** | *Ptilonorhynchus violaceus* | 85 | | *Myrsidea ptilonorhynchi* | contact and environmental | ^6^ |
| **Caine and Melfi 2006** | *Colobus guereza kikiyuensis* | 6 | | *Trichuris trichiura* | environmental | ^14^ |
| **Clough et al. 2010 A, D, F** | *Eulemur fulvus rufus* | 10-11 | | *Lemuricola vauceli* | unknown | ^37^ |
| **Clough et al. 2010 A, D, F** | *Eulemur fulvus rufus* | 10-11 | | *Callistoura* sp. | unknown | ^38^ |
| **Clough et al. 2010 A, D, F** | *Eulemur fulvus rufus* | 10-11 | | *Trichuris* sp. | environmental | ^39^ |
| **Clough et al. 2010 A, D, F** | *Eulemur fulvus rufus* | 10-11 | | Trichostrongylidae | environmental | ^39^ |
| **Clough et al. 2010 A, D, F** | *Eulemur fulvus rufus* | 10-11 | | *Strongyloides* | environmental | ^36^ |
| **Clough et al. 2010 B, E, F** | *Eulemur fulvus rufus* | 10-11 | | *Entamoeba coli* | contact and environmental | ^36^ |
| **Colombelli-Négrel and Kleindorfer 2008** | *Malurus cyaneus* | 51 | | *Haemoproteus spp.* | flying vector | ^40^ |
| **Dufva and Allander 1995 A** | *Parus major* | 37 | | *Haemoproteus majoris* | flying vector | ^40^ |
| **Dufva and Allander 1995 B** | *Parus major* | 37 | | *Hepatozoon parus* | flying vector | ^40^ |
| **Dufva and Allander 1995 C** | *Parus major* | 37 | | *Plasmodium vaughani* | flying vector | ^40^ |
| **Fugazzola and Stancampiano 2012 A, B** | *Equus quagga* | 18 | | Strongyles | environmental | ^39^ |
| **Fugazzola and Stancampiano 2012 C, D** | *Equus quagga* | 18 | | Cestodes | environmental | ^39^ |
| **Fugazzola and Stancampiano 2012 E, F** | *Equus quagga* | 18 | | Oxiurids | contact and environmental | ^39^ |
| **Fugazzola and Stancampiano 2012 G, H** | *Equus quagga* | 18 | | *Strongyloides* sp. | environmental | ^39^ |
| **Hausfater and Watson 1976** | *Papio anubis* | 8 | | *Trichuris sp.* | environmental | ^36^ |
| **Hausfater and Watson 1976** | *Papio anubis* | 8 | | *Trichostrongylus* | environmental | ^36^ |
| **Huyghe et al. 2009** | *Podacris melisellensis* | 34 | | Ectoparasites; not specified | contact and environmental | ^41^ |
| **Meade 1984 A** | *Papio cynocephalus* | 8 | | *Abbreviata* | environmental (non-biting vector) | ^42^ |
| **Meade 1984 A** | *Papio cynocephalus* | 8 | | *Strongyloides* | environmental | ^36^ |
| **Meade 1984 A** | *Papio cynocephalus* | 8 | | *Oesophagostomum* | environmental | ^36^ |
| **Meade 1984 A** | *Papio cynocephalus* | 8 | | *Physaloptera* | environmental (non-biting vector) | ^36^ |
| **Meade 1984 A** | *Papio cynocephalus* | 8 | | *Trichostrongylus* | environmental | ^36^ |
| **Meade 1984 A, B** | *Papio cynocephalus* | 8 | | *Trichuris trichiura* | environmental | ^36^ |
| **Melfi and Poyser 2007** | *Colobus guereza kikiyuensis* | 6 | | *Trichuris trichiura* | environmental | ^14^ |
| **Mooring et al. 1996** | *Impala (African antelope)* | 18 | | *Boophilus decoloratus* | contact and environmental | ^41^ |
| **Mooring et al. 1996** | *Impala (African antelope)* | 18 | | *Rhipicephalus appendiculatus* | contact and environmental | ^41^ |
| **Mooring et al. 1996** | *Impala (African antelope)* | 18 | | *Rhipicephalus evertsi* | contact and environmental | ^41^ |
| **Mooring et al. 1996** | *Impala (African antelope)* | 18 | | *Ixodes* sp. | contact and environmental | ^41^ |
| **Muehlenbein 2006** | *Pan troglodytes* | 12 | | *Strongyloides* sp. | environmental | ^36^ |
| **Muehlenbein 2006** | *Pan troglodytes* | 12 | | *Entamoeba chattoni* | contact and environmental | ^36^ |
| **Muehlenbein 2006** | *Pan troglodytes* | 12 | | *Troglodytella abrassarti* | environmental | ^43^ |
| **Muehlenbein 2006** | *Pan troglodytes* | 12 | | *Oesophagostomum* sp. | environmental | ^36^ |
| **Muehlenbein and Watts 2010 A** | *Pan troglodytes* | 22 | | *Probstmayria* sp. | environmental | ^44^ |
| **Muehlenbein and Watts 2010 A** | *Pan troglodytes* | 22 | | *Hymenolepis* sp. | environmental | ^44^ |
| **Muehlenbein and Watts 2010 A** | *Pan troglodytes* | 22 | | *Physaloptera* | environmental (non-biting vector) | ^36^ |
| **Muehlenbein and Watts 2010 A** | *Pan troglodytes* | 22 | | *Oesophagostomum* sp. | environmental | ^36^ |
| **Muehlenbein and Watts 2010 A** | *Pan troglodytes* | 22 | | *Strongyloides* | environmental | ^36^ |
| **Muehlenbein and Watts 2010 B** | *Pan troglodytes* | 22 | | *Endolimax* sp. | contact and environmental | ^45^ |
| **Muehlenbein and Watts 2010 B** | *Pan troglodytes* | 22 | | *Troglodytella abrassarti* | environmental | ^43^ |
| **Muehlenbein and Watts 2010 B** | *Pan troglodytes* | 22 | | *Entamoeba coli* | contact and environmental | ^36^ |
| **Muehlenbein and Watts 2010 B** | *Pan troglodytes* | 22 | | *Entamoeba hartmanni* | contact and environmental | ^36^ |
| **Muehlenbein and Watts 2010 B** | *Pan troglodytes* | 22 | | *Entamoeba chattoni* | contact and environmental | ^36^ |
| **Muehlenbein and Watts 2010 B** | *Pan troglodytes* | 22 | | *Iodamoeba* sp. | contact and environmental | ^36^ |
| **Muehlenbein and Watts 2010 B** | *Pan troglodytes* | 22 | | *Blastocystis* sp. | unknown | ^44^ |
| **Muller-Graf et al. 1996 A, B** | *Papio anubis* | 27 | | *Physaloptera* | environmental (non-biting vector) | ^36^ |
| **Muller-Graf et al. 1996 A, B** | *Papio anubis* | 27 | | *Streptopharagus* sp. | environmental | ^36^ |
| **Muller-Graf et al. 1996 A, B** | *Papio anubis* | 27 | | *Schistosoma mansoni* | environmental | ^36^ |
| **Muller-Graf et al. 1996 A, B** | *Papio anubis* | 27 | | *Paragonimus* sp. (unidentified trematode) | environmental | ^36^ |
| **Muller-Graf et al. 1996 A, B** | *Papio anubis* | 27 | | *Strongyloides* sp. | environmental | ^36^ |
| **Muller-Graf et al. 1996 A, B** | *Papio anubis* | 27 | | *Oesophagostomum* sp. | environmental | ^36^ |
| **Muller-Graf et al. 1996 A, B** | *Papio anubis* | 27 | | *Trichuris* sp. | environmental | ^36^ |
| **Muller-Graf et al. 1996 A, B** | *Papio anubis* | 27 | | *Necator americanus* | environmental | ^36^ |
| **Muller-Graf et al. 1996 A, B** | *Papio anubis* | 27 | | *Trichostrongylus* sp. | environmental | ^36^ |
| **Negro et al. 2010** | *Arctocephalus forsteri* | 12 | | *Diphylobothrium* | environmental | ^39^ |
| **Negro et al. 2010** | *Arctocephalus forsteri* | 12 | | *Anisakis* | environmental | ^46^ |
| **Negro et al. 2010** | *Arctocephalus forsteri* | 12 | | *Otostrongylus* | environmental | ^47^ |
| **Negro et al. 2010** | *Arctocephalus forsteri* | 12 | | *Contracaecum* | environmental | ^46^ |
| **Negro et al. 2010** | *Arctocephalus forsteri* | 12 | | *Synthesium* | environmental | ^48^ |
| **Negro et al. 2010** | *Arctocephalus forsteri* | 12 | | *Pricetrema* | environmental | ^48^ |
| **Negro et al. 2010** | *Arctocephalus forsteri* | 12 | | *Zalophotrema* | environmental | ^48^ |
| **Negro et al. 2010** | *Arctocephalus forsteri* | 12 | | *Acanthocephala bolbosoma* | environmental (non-biting vector) | ^48^ |
| **Negro et al. 2010** | *Arctocephalus forsteri* | 12 | | *Acanthocephala corynosoma* | environmental (non-biting vector) | ^48^ |
| **Pelletier et al. 2005** | *Ovis canadensis* | 51 | | *Protostrongylus stilesi* | environmental (non-biting vector) | ^19^ |
| **Pelletier et al. 2005** | *Ovis canadensis* | 51 | | *Protostrongylus rushi* | environmental (non-biting vector) | ^19^ |
| **Poiani et al. 2000** | *Passer domesticus* | 28 | | *Ceratophyllus gallinae* | contact and environmental | ^41^ |
| **Poiani et al. 2000** | *Passer domesticus* | 28 | | *Philopterus fringillidae* | contact and environmental | ^41^ |
| **Poiani et al. 2000** | *Passer domesticus* | 28 | | *Brucelia* sp. | contact and environmental | ^41^ |
| **Poiani et al. 2000** | *Passer domesticus* | 28 | | *Dermanyssus gallinae* | contact and environmental | ^41^ |
| **Poiani et al. 2000** | *Passer domesticus* | 28 | | Analgidae | contact and environmental | ^41^ |
| **Poiani et al. 2000** | *Passer domesticus* | 28 | | Proctophyllodidae | contact and environmental | ^41^ |
| **Schall and Houle 1992** | *Sceloporus occidentalis* | 26 | | *Plasmodium mexicanum* | flying vector | ^40^ |
| **Schall and Staats 2002** | *Anolis sabanus* | 32 | | *Plasmodium floridense* | flying vector | ^40^ |
| **Schall and Staats 2002** | *Anolis sabanus* | 32 | | *Plasmodium azurophilum* | flying vector | ^40^ |
| **Schall and Staats 2002** | *Anolis sabanus* | 32 | | *Plasmodium* sp. | flying vector | ^40^ |
| **Teichroeb et al. 2009** | *Colobus vellerosus* | 14 | | *Isospora* sp. | contact and environmental | ^27^ |
| **Teichroeb et al. 2009** | *Colobus vellerosus* | 14 | | *Giardia duodenalis* | contact and environmental | ^27^ |
| **Teichroeb et al. 2009** | *Colobus vellerosus* | 14 | | *Ascaris* sp. | contact and environmental | ^49^ |
| **Teichroeb et al. 2009** | *Colobus vellerosus* | 14 | | *Enterobius* sp. | contact and environmental | ^50^ |
| **Teichroeb et al. 2009** | *Colobus vellerosus* | 14 | | Digenean trematode | environmental | ^48^ |
| **Teichroeb et al. 2009** | *Colobus vellerosus* | 14 | | *Entamoeba histolytica/dispar* | contact and environmental | ^36^ |
| **Teichroeb et al. 2009** | *Colobus vellerosus* | 14 | | *Entamoeba coli* | contact and environmental | ^36^ |
| **Teichroeb et al. 2009** | *Colobus vellerosus* | 14 | | *Strongyles* sp. | environmental | ^36^ |
| **Teichroeb et al. 2009** | *Colobus vellerosus* | 14 | | *Trichuris* | environmental | ^14^ |
| **Teichroeb et al. 2009** | *Colobus vellerosus* | 14 | | *Blastocystis* sp. | unknown | ^44^ |
| **Weatherhead et al. 1993 A** | *Agelaius phoeniceus* | 45 | | Lice (Mallophaga) | contact and environmental | ^51^ |
| **Weatherhead et al. 1993 B** | *Agelaius phoeniceus* | 45 | | *Haemoproteus quiscalus* | flying vector | ^51^ |
| **Weatherhead et al. 1993 B** | *Agelaius phoeniceus* | 45 | | *Leuccytozoon* spp. | flying vector | ^51^ |
| **Weatherhead et al. 1993 B** | *Agelaius phoeniceus* | 45 | | *Plasmodium vaughani* | flying vector | ^51^ |
| **Weatherhead et al. 1993 B** | *Agelaius phoeniceus* | 45 | | Microfilaria (*Eufilaria hibleri*; *Splendidofilaria quiscali*) | flying vector | ^51^ |
| **Weatherhead et al. 1993 B** | *Agelaius phoeniceus* | 45 | | Trematodes (*Plagiorchis* sp.; *Conspicuum* sp.) | environmental (non-biting vector) | ^51^ |
| **Weatherhead et al. 1993 B** | *Agelaius phoeniceus* | 45 | | Cestodes | environmental (non-biting vector) | ^51^ |
| **female studies** | | | | | | |
| **Akinyi et al. 2013 A, B** | *Papio cynocephalus* | | 38 | *Rhipicephalus simus* | contact and environmental | ^35^ |
| **Akinyi et al. 2013 A, B** | *Papio cynocephalus* | | 38 | *Rhipicephalus pulchellus* | contact and environmental | ^35^ |
| **Akinyi et al. 2013 A, B** | *Papio cynocephalus* | | 38 | *Hyalomma truncatum* | contact and environmental | ^35^ |
| **Foerster et al. 2015 A** | *Cercopithecus mitis albogularis* | | 11 | *Strongyloides* | environmental | ^39^ |
| **Foerster et al. 2015 B** | *Cercopithecus mitis albogularis* | | 11 | *Trichostrongylus* | environmental | ^36^ |
| **Foerster et al. 2015 C** | *Cercopithecus mitis albogularis* | | 11 | *Trichuris* | environmental | ^36^ |
| **Foerster et al. 2015 D** | *Cercopithecus mitis albogularis* | | 11 | *Oesophagostomum* | environmental | ^36^ |
| **Foerster et al. 2015 E** | *Cercopithecus mitis albogularis* | | 11 | *Streptopharagus* | environmental | ^36^ |
| **Foerster et al. 2015 F** | *Cercopithecus mitis stuhlmanni* | | 7 | *Trichuris* | environmental | ^36^ |
| **Foerster et al. 2015 G** | *Cercopithecus mitis stuhlmanni* | | 10 | *Trichuris* | environmental | ^36^ |
| **Fugazzola and Stancampiano 2012 A, B** | *Equus quagga* | | 70 | Strongyles | environmental | ^39^ |
| **Fugazzola and Stancampiano 2012 C, D** | *Equus quagga* | | 70 | Cestodes | environmental | ^39^ |
| **Fugazzola and Stancampiano 2012 E, F** | *Equus quagga* | | 70 | Oxiurids | contact and environmental | ^39^ |
| **Fugazzola and Stancampiano 2012 G, H** | *Equus quagga* | | 70 | *Strongyloides* sp. | environmental | ^39^ |
| **Hausfater and Watson 1976** | *Papio cynocephalus* | | 29 | *Trichuris* | environmental | ^36^ |
| **Hausfater and Watson 1976** | *Papio cynocephalus* | | 29 | *Trichostrongylus*sp. | environmental | ^36^ |
| **Hernandez et al. 2009 A** | *Macaca fuscata yakui* | | 13 | *Oesophagostomum aculateum* | environmental | ^32^ |
| **Hernandez et al. 2009 B** | *Macaca fuscata yakui* | | 13 | *Streptopharagus pigmentatus* | environmental | ^32^ |
| **MacIntosh et al. 2012 A** | *Macaca fuscata yakui* | | 12 | *Strongyloides fuelleborni* | environmental | ^36^ |
| **MacIntosh et al. 2012 A** | *Macaca fuscata yakui* | | 12 | *Trichuris trichiura* | environmental | ^14^ |
| **MacIntosh et al. 2012 A, B, C** | *Macaca fuscata yakui* | | 12 | *Oesophagostomum aculateum* | environmental | ^32^ |
| **Meade 1984 A** | *Papio cynocephalus* | | 10 | *Abbreviata* sp. | environmental (non-biting vector) | ^42^ |
| **Meade 1984 A** | *Papio cynocephalus* | | 10 | *Physaloptera* sp. | environmental (non-biting vector) | ^36^ |
| **Meade 1984 A** | *Papio cynocephalus* | | 10 | *Oesophagostomum aculateum* | environmental | ^32^ |
| **Meade 1984 A** | *Papio anubis* | | 10 | *Strongyloides* sp. | environmental | ^36^ |
| **Meade 1984 A** | *Papio cynocephalus* | | 10 | *Trichostrongylus* sp. | environmental | ^36^ |
| **Meade 1984 A, B** | *Papio cynocephalus* | | 10 | *Trichuris* | environmental | ^36^ |
| **Muller-Graf et al. 1996 A, B** | *Papio anubis* | | 63 | *Paragonimus sp.* (unidentified trematode) | environmental | ^36^ |
| **Muller-Graf et al. 1996 A, B** | *Papio anubis* | | 63 | *Strongyloides sp.* | environmental | ^36^ |
| **Müller-Graf, Collins, and Woolhouse 1996 A, B** | *Papio anubis* | | 63 | *Physaloptera* sp. | environmental (non-biting vector) | ^36^ |
| **Müller-Graf, Collins, and Woolhouse 1996 A, B** | *Papio anubis* | | 63 | *Streptopharagus* sp. | environmental | ^36^ |
| **Müller-Graf, Collins, and Woolhouse 1996 A, B** | *Papio anubis* | | 63 | *Schistosoma mansoni* | environmental | ^36^ |
| **Müller-Graf, Collins, and Woolhouse 1996 A, B** | *Papio anubis* | | 63 | *Trichuris* sp. | environmental | ^36^ |
| **Müller-Graf, Collins, and Woolhouse 1996 A, B** | *Papio anubis* | | 63 | *Trichostrongylus* sp. | environmental | ^36^ |
| **Müller-Graf, Collins, and Woolhouse 1996 A, B** | *Papio anubis* | | 63 | *Oesophagostomum* sp. | environmental | ^36^ |
| **Müller-Graf, Collins, and Woolhouse 1996 A, B** | *Papio anubis* | | 63 | *Necator americanus* | environmental | ^36^ |
| **Ravasi 2009 A** | *Papio ursinus* | | 12 | *Trichostrongylus* sp. | environmental | ^36^ |
| **Ravasi 2009 B** | *Papio ursinus* | | 12 | *Physaloptera* sp. | environmental (non-biting vector) | ^36^ |
| **Ravasi 2009 C** | *Papio ursinus* | | 12 | *Ascaris* sp. | contact and environmental | ^49^ |
| **Ravasi 2009 D** | *Papio ursinus* | | 12 | *Spirurid* | environmental (non-biting vector) | ^52^ |
| **Ravasi 2009 E** | *Papio ursinus* | | 12 | *Entamoeba histolytica/dispar* | contact and environmental | ^36^ |
| **Ravasi 2009 F** | *Papio ursinus* | | 12 | *Entamoeba chattoni* | contact and environmental | ^36^ |
| **Ravasi 2009 G** | *Papio ursinus* | | 12 | *Entamoeba hartmanni* | contact and environmental | ^36^ |
| **Ravasi 2009 H** | *Papio ursinus* | | 12 | *Chilomastix mesnili* | contact and environmental | ^36^ |
| **Ravasi 2009 I** | *Papio ursinus* | | 12 | *Endolimax nana* | contact and environmental | ^45^ |
| **Setchell et al. 2007 A, D** | *Mandrillus Sphinx* | | 11 | *Mammomonogamus* | contact and environmental | ^48^ |
| **Setchell et al. 2007 A, D** | *Mandrillus Sphinx* | | 11 | *Trichuris* | environmental | ^36^ |
| **Setchell et al. 2007 B, E** | *Mandrillus Sphinx* | | 11 | *Balantidium coli* | contact and environmental | ^36^ |
| **Setchell et al. 2007 C, F** | *Mandrillus Sphinx* | | 11 | *Entamoeba histolytica/dispar* | contact and environmental | ^36^ |
| **Setchell et al. 2007 G** | *Mandrillus Sphinx* | | 11 | *Entamoeba coli* | contact and environmental | ^36^ |
| **Setchell et al. 2007 H** | *Mandrillus Sphinx* | | 11 | *Endolimax nana* | contact and environmental | ^53^ |
| **Smyth and Drea 2015 A, B** | *Suricata suricatta* | | 35 | Strongylate nematode | environmental | ^39^ |
| **Smyth and Drea 2015 A, C** | *Suricata suricatta* | | 35 | *Toxocara suricattae* | environmental | ^39^ |
| **Smyth and Drea 2015 A, D** | *Suricata suricatta* | | 35 | Spirurida nematode | environmental (non-biting vector) | ^39^ |
| **Smyth and Drea 2015 A, E** | *Suricata suricatta* | | 35 | *Oxynema suricattae* | environmental | ^29^ |
| **Smyth and Drea 2015 A, F** | *Suricata suricatta* | | 35 | *Pseudandrya suricattae* | environmental (non-biting vector) | ^29^ |
| **Smyth and Drea 2015 A, G** | *Suricata suricatta* | | 35 | Coccidia | environmental | ^39^ |
| **Ungerfeld and Correa, 2007** | *Capra aegagrus* | | 27 | Not specified - General FEC | not enough information | ^54^ |

**Table S11.** Relative allostatic load and relative parasitism in dominants and subordinates across 26 studies and 66 analyses of male vertebrates and 13 studies and 62 analyses of female vertebrates; Note: In studies where multiple analyses were conducted, letters following authors’ names (i.e. A, B, C, etc.) were used to indicate an individual analysis.

| Study | Dominant Rank Acquisition | Dominant Rank Maintenance | Subordinate Degree of Threat | Subordinate Outlets | Dominant Resource Control | Subordinate Resource Control | Dominant Allostatic Load | Subordinate Allostatic Load | Relative Allostatic Load | Citation used to determine Allostatic Load | Dominant Parasitism | Subordinate Parasitism | Relative Parasitism |
| --- | --- | --- | --- | --- | --- | --- | --- | --- | --- | --- | --- | --- | --- |
| **male studies** | | | | | | | | | | | | | |
| **Akinyi et al. 2013 A** | 2 | 2 | 3 | 2 | 0 | 0 | 4 | 5 | 0.80 | Akinyi, M; Alberts, SC; Archie, EA; Fitzpatrick, C; Tung, J (personal communication) | 19.23 | 40.91 | 0.47 |
| **Akinyi et al. 2013 B** | 2 | 2 | 3 | 2 | 0 | 0 | 4 | 5 | 0.80 | Akinyi, M; Alberts, SC; Archie, EA; Fitzpatrick, C; Tung, J (personal communication) | 21.69 | 3.09 | 7.02 |
| **Arlet et al. 2015 A** | 2 | 3 | 2 | 1 | 0 | 0 | 5 | 3 | 1.67 | Arlet. M (personal communication) | 0.19 | 0.32 | 0.59 |
| **Arlet et al. 2015 B** | 2 | 3 | 2 | 1 | 0 | 0 | 5 | 3 | 1.67 | Arlet. M (personal communication) | 0.24 | 0.87 | 0.27 |
| **Arlet et al. 2015 C** | 2 | 3 | 2 | 1 | 0 | 0 | 5 | 3 | 1.67 | Arlet. M (personal communication) | 0.60 | 0.07 | 8.40 |
| **Arlet et al. 2015 D** | 2 | 3 | 2 | 1 | 0 | 0 | 5 | 3 | 1.67 | Arlet. M (personal communication) | 0.03 | 0.09 | 0.34 |
| **Arlet et al. 2015 E** | 2 | 3 | 2 | 1 | 0 | 0 | 5 | 3 | 1.67 | Arlet. M (personal communication) | 0.26 | 0.07 | 3.61 |
| **Bartoli et al. 2000 A** | 1 | 1.25 | 1.25 | 1.5 | 0 | 0 | 2.25 | 2.75 | 0.83 | Doellman, M.; Habig, B.; Olansen, J; Woods, K (citation: ^5^) | 0.28 | 0.02 | 11.95 |
| **Bartoli et al. 2000 B** | 1 | 1.25 | 1.25 | 1.5 | 0 | 0 | 2.25 | 2.75 | 0.83 | Doellman, M.; Habig, B.; Olansen, J; Woods, K (citation: ^5^) | 0.13 | 0.08 | 1.52 |
| **Bartoli et al. 2000 C** | 1 | 1.25 | 1.25 | 1.5 | 0 | 0 | 2.25 | 2.75 | 0.83 | Doellman, M.; Habig, B.; Olansen, J; Woods, K (citation: ^5^) | NA | NA |  |
| **Borgia and Collis 1990** | 2 | 3 | 3 | 1 | 0 | 0 | 5 | 4 | 1.25 | Borgia, G. (personal communication) | 7.6 | 11.4 | 0.67 |
| **Caine and Melfi 2006** | 2 | 2 | 2 | 2 | 0 | 1 | 4 | 5 | 0.80 | Melfi, V (personal communication); Due to captivity, no outlets | NA | NA | NA |
| **Clough et al. 2010 A** | 2 | 2 | 2 | 1 | 0 | 0 | 4 | 3 | 1.33 | Clough, D. (personal communication) | 235.59 | 287.41 | 0.82 |
| **Clough et al. 2010 B** | 2 | 2 | 2 | 1 | 0 | 0 | 4 | 3 | 1.33 | Clough, D. (personal communication) | 1356.78 | 1137.05 | 1.19 |
| **Clough et al. 2010 C** | 2 | 2 | 2 | 1 | 0 | 0 | 4 | 3 | 1.33 | Clough, D. (personal communication) | 2.78 | 2.66 | 1.05 |
| **Clough et al. 2010 D** | 2 | 2 | 2 | 1 | 0 | 0 | 4 | 3 | 1.33 | Clough, D. (personal communication) | 258.65 | 160.97 | 1.61 |
| **Clough et al. 2010 E** | 2 | 2 | 2 | 1 | 0 | 0 | 4 | 3 | 1.33 | Clough, D. (personal communication) | 1652.89 | 2076.76 | 0.80 |
| **Clough et al. 2010 F** | 2 | 2 | 2 | 1 | 0 | 0 | 4 | 3 | 1.33 | Clough, D. (personal communication) | 2.37 | 2.31 | 1.03 |
| **Colombelli-Négrel and Kleindorfer 2008** | 1 | 1 | 1 | 1 | 0 | 0 | 2 | 2 | 1.00 | Colombelli-Négrel, D (personal communication) | 0.258 | 0.05 | 5.16 |
| **Dufva and Allander 1995 A** | 2 | 2 | 2 | 1 | 0 | 0 | 4 | 3 | 1.33 | Allander, K (personal communication) | NA | NA | NA |
| **Dufva and Allander 1995 B** | 2 | 2 | 2 | 1 | 0 | 0 | 4 | 3 | 1.33 | Allander, K (personal communication) | NA | NA | NA |
| **Dufva and Allander 1995 C** | 2 | 2 | 2 | 1 | 0 | 0 | 4 | 3 | 1.33 | Allander, K (personal communication) | NA | NA | NA |
| **Fugazzola and Stancampiano 2012 A** | 2 | 3 | 2 | 2 | 0 | 1 | 5 | 5 | 1.00 | Fugazzola, MC and Stancampiano, L (personal communication) | 1.00 | 1.00 | 1.00 |
| **Fugazzola and Stancampiano 2012 B** | 2 | 3 | 2 | 2 | 0 | 1 | 5 | 5 | 1.00 | Fugazzola, MC and Stancampiano, L (personal communication) | 187.14 | 383.33 | 0.49 |
| **Fugazzola and Stancampiano 2012 C** | 2 | 3 | 2 | 2 | 0 | 1 | 5 | 5 | 1.00 | Fugazzola, MC and Stancampiano, L (personal communication) | 0.07 | 0.28 | 0.26 |
| **Fugazzola and Stancampiano 2012 D** | 2 | 3 | 2 | 2 | 0 | 1 | 5 | 5 | 1.00 | Fugazzola, MC and Stancampiano, L (personal communication) | 1.43 | 8.89 | 0.16 |
| **Fugazzola and Stancampiano 2012 E** | 2 | 3 | 2 | 2 | 0 | 1 | 5 | 5 | 1.00 | Fugazzola, MC and Stancampiano, L (personal communication) | 0.43 | 0.39 | 1.10 |
| **Fugazzola and Stancampiano 2012 F** | 2 | 3 | 2 | 2 | 0 | 1 | 5 | 5 | 1.00 | Fugazzola, MC and Stancampiano, L (personal communication) | 15.71 | 14.44 | 1.09 |
| **Fugazzola and Stancampiano 2012 G** | 2 | 3 | 2 | 2 | 0 | 1 | 5 | 5 | 1.00 | Fugazzola, MC and Stancampiano, L (personal communication) | 0.00 | 0.22 | 0.00 |
| **Fugazzola and Stancampiano 2012 H** | 2 | 3 | 2 | 2 | 0 | 1 | 5 | 5 | 1.00 | Fugazzola, MC and Stancampiano, L (personal communication) | 0.00 | 7.78 | 0.00 |
| **Hausfater and Watson 1976** | 2 | 2 | 3 | 2 | 0 | 0 | 4 | 5 | 0.80 | Alberts, SC; Archie, EA; Fitzpatrick, C; Tung, J (personal communication) | 26.63 | 11.25 | 2.37 |
| **Huyghe et al. 2009** | 2 | 2 | 2 | 1 | 0 | 1 | 4 | 4 | 1.00 | Huyghe, K. (personal communication) | 1.44 | 0.69 | 2.09 |
| **Leclaire and Faulkner 2014 A** | 1.5 | 2 | 2 | 2 | 0 | 0 | 3.5 | 4 | 0.88 | Smyth, KN (personal communication) | 3.67 | 2.63 | 1.39 |
| **Leclaire and Faulkner 2014 B** | 1.5 | 2 | 2 | 2 | 0 | 0 | 3.5 | 4 | 0.88 | Smyth, KN (personal communication) | 2.67 | 0.84 | 3.17 |
| **Leclaire and Faulkner 2014 C** | 1.5 | 2 | 2 | 2 | 0 | 0 | 3.5 | 4 | 0.88 | Smyth, KN (personal communication) | 1.67 | 1.74 | 0.96 |
| **Leclaire and Faulkner 2014 D** | 1.5 | 2 | 2 | 2 | 0 | 0 | 3.5 | 4 | 0.88 | Smyth, KN (personal communication) | 0.33 | 0.47 | 0.70 |
| **Leclaire and Faulkner 2014 E** | 1.5 | 2 | 2 | 2 | 0 | 0 | 3.5 | 4 | 0.88 | Smyth, KN (personal communication) | 1 | 1 | 1.00 |
| **Leclaire and Faulkner 2014 F** | 1.5 | 2 | 2 | 2 | 0 | 0 | 3.5 | 4 | 0.88 | Smyth, KN (personal communication) | 0.67 | 0.68 | 0.97 |
| **Leclaire and Faulkner 2014 G** | 1.5 | 2 | 2 | 2 | 0 | 0 | 3.5 | 4 | 0.88 | Smyth, KN (personal communication) | 0.67 | 0.37 | 1.81 |
| **Leclaire and Faulkner 2014 H** | 1.5 | 2 | 2 | 2 | 0 | 0 | 3.5 | 4 | 0.88 | Smyth, KN (personal communication) | 0.33 | 0.26 | 1.27 |
| **Leclaire and Faulkner 2014 I** | 1.5 | 2 | 2 | 2 | 0 | 0 | 3.5 | 4 | 0.88 | Smyth, KN (personal communication) | 0.33 | 0.47 | 0.70 |
| **Leclaire and Faulkner 2014 J** | 1.5 | 2 | 2 | 2 | 0 | 0 | 3.5 | 4 | 0.88 | Smyth, KN (personal communication) | 0.67 | 0.32 | 2.11 |
| **Meade 1984 A** | 2 | 2 | 3 | 2 | 0 | 0 | 4 | 5 | 0.80 | Alberts, SC; Archie, EA; Fitzpatrick, C; Tung, J (personal communication) | 4.22 | 2.07 | 2.04 |
| **Meade 1984 B** | 2 | 2 | 3 | 2 | 0 | 0 | 4 | 5 | 0.80 | Alberts, SC; Archie, EA; Fitzpatrick, C; Tung, J (personal communication) | 3.46 | 1.75 | 1.98 |
| **Melfi and Poyser 2007** | 2 | 2 | 2 | 2 | 0 | 1 | 4 | 5 | 0.80 | Melfi, V (personal communication) | NA | NA | NA |
| **Mooring et al. 1996** | 2 | 3 | 3 | 1 | 0 | 1 | 5 | 5 | 1.00 | Mooring, M (personal communication) | 21841.28 | 9335.35 | 2.34 |
| **Muehlenbein and Watts 2010 A** | 2 | 3 | 2 | 1 | 0 | 0 | 5 | 3 | 1.67 | Author did not respond; used data from ^55^: ^56^ | NA | NA | NA |
| **Muehlenbein and Watts 2010 B** | 2 | 3 | 2 | 1 | 0 | 0 | 5 | 3 | 1.67 | Author did not respond; used data from ^55^: ^56^ | NA | NA | NA |
| **Muehlenbein et al. 2004** | 2 | 3 | 2 | 1 | 0 | 0 | 5 | 3 | 1.67 | Author did not respond; used data from ^55^: ^56^ | NA | NA | NA |
| **Muller-Graf et al. 1996** | 2 | 2 | 3 | 2 | 0 | 0 | 4 | 5 | 0.80 | Author did not respond; used data from ^55^: ^57^ | NA | NA | NA |
| **Muller-Graf et al. 1996** | 2 | 2 | 3 | 2 | 0 | 0 | 4 | 5 | 0.80 | Author did not respond; used data from ^55^: ^57^ | NA | NA | NA |
| **Negro et al. 2010** | 2 | 2 | 2.5 | 1 | 0 | 0 | 4 | 3.5 | 1.14 | Negro, S (personal communication) | 6.2 | 2.5 | 2.48 |
| **Pelletier et al. 2005** | 2 | 3 | 2 | 1 | 0 | 0 | 5 | 3 | 1.67 | Pelletier, F. (personal communication) | NA | NA | NA |
| **Poiani et al. 2000** | 1.75 | 2.5 | 2 | 1.25 | 0 | 0.5 | 4.25 | 3.75 | 1.13 | Doellman, M.; Habig, B.; Olansen, J; Woods, K (citation: ^21^) | NA | NA | NA |
| **Schall and Houle 1992** | 2 | 3 | 3 | 1 | 0 | 1 | 5 | 5 | 1.00 | Olansen, J. Woods, K (citation: ^22^) | 0.00 | 0.67 | 0.00 |
| **Schall and Staats 2002** | 2 | 3 | 3 | 1 | 0 | 1 | 5 | 5 | 1.00 | Olansen, J. Woods, K (citation: ^24^) | 0.5 | 0.5 | 1.00 |
| **Smyth and Drea 2015 A** | 1.5 | 2 | 2 | 2 | 0 | 0 | 3.5 | 4 | 0.88 | Smyth, KN (personal communication) | 3.14 | 2.38 | 1.32 |
| **Smyth and Drea 2015 B** | 1.5 | 2 | 2 | 2 | 0 | 0 | 3.5 | 4 | 0.88 | Smyth, KN (personal communication) | 0.93 | 0.81 | 1.15 |
| **Smyth and Drea 2015 C** | 1.5 | 2 | 2 | 2 | 0 | 0 | 3.5 | 4 | 0.88 | Smyth, KN (personal communication) | 0.71 | 0.29 | 2.45 |
| **Smyth and Drea 2015 D** | 1.5 | 2 | 2 | 2 | 0 | 0 | 3.5 | 4 | 0.88 | Smyth, KN (personal communication) | 0.29 | 0.21 | 1.38 |
| **Smyth and Drea 2015 E** | 1.5 | 2 | 2 | 2 | 0 | 0 | 3.5 | 4 | 0.88 | Smyth, KN (personal communication) | 0.5 | 0.44 | 1.14 |
| **Smyth and Drea 2015 F** | 1.5 | 2 | 2 | 2 | 0 | 0 | 3.5 | 4 | 0.88 | Smyth, KN (personal communication) | 0 | 0.083 | 0.00 |
| **Smyth and Drea 2015 G** | 1.5 | 2 | 2 | 2 | 0 | 0 | 3.5 | 4 | 0.88 | Smyth, KN (personal communication) | 0.71 | 0.52 | 1.37 |
| **Teichroeb et al. 2009** | 2 | 3 | 2 | 2 | 0 | 1 | 5 | 5 | 1.00 | Teichroeb, J (personal communication) | NA | NA | NA |
| **Weatherhead et al. 1993** | 2 | 2.5 | 2 | 1 | 0 | 0.5 | 4.5 | 3.5 | 1.46 | Doellman, M.; Habig, B.; Olansen, J; Woods, K (citation: ^58^) | NA | NA | NA |
| **female studies** | | | | | | | | | | | | | |
| **Akinyi et al. 2013 A** | 1 | 2 | 2 | 1 | 0 | 1 | 3 | 4 | 0.75 | Akinyi, M; Alberts, SC; Archie, EA; Fitzpatrick, C; Tung, J (personal communication) | 2.90 | 11.33 | 0.26 |
| **Akinyi et al. 2013 B** | 1 | 2 | 2 | 1 | 0 | 1 | 3 | 4 | 0.75 | Akinyi, M; Alberts, SC; Archie, EA; Fitzpatrick, C; Tung, J (personal communication) | 4.15 | 8.11 | 0.51 |
| **Foerster et al. 2015 A** | 1 | 1.5 | 2 | 1 | 0 | 0.25 | 2.5 | 3.25 | 0.77 | Foerster, S (personal communication) | 125.20 | 134.83 | 0.93 |
| **Foerster et al. 2015 B** | 1 | 1.5 | 2 | 1 | 0 | 0.25 | 2.5 | 3.25 | 0.77 | Foerster, S (personal communication) | 92.60 | 43.83 | 2.11 |
| **Foerster et al. 2015 C** | 1 | 1.5 | 2 | 1 | 0 | 0.25 | 2.5 | 3.25 | 0.77 | Foerster, S (personal communication) | 43.70 | 96.80 | 0.45 |
| **Foerster et al. 2015 D** | 1 | 1.5 | 2 | 1 | 0 | 0.25 | 2.5 | 3.25 | 0.77 | Foerster, S (personal communication) | 54.20 | 38.17 | 1.42 |
| **Foerster et al. 2015 E** | 1 | 1.5 | 2 | 1 | 0 | 0.25 | 2.5 | 3.25 | 0.77 | Foerster, S (personal communication) | 0.00 | 16.00 | 0.00 |
| **Foerster et al. 2015 F** | 1 | 1.5 | 1 | 1 | 0 | 0.25 | 2.5 | 2.25 | 1.11 | Foerster, S (personal communication) | NA | NA | NA |
| **Foerster et al. 2015 G** | 1 | 1.5 | 1 | 1 | 0 | 0.25 | 2.5 | 2.25 | 1.11 | Foerster, S (personal communication) | NA | NA | NA |
| **Fugazzola and Stancampiano 2012 A** | 2 | 1.5 | 1 | 2 | 0 | 1 | 3.5 | 4 | 0.88 | Fugazzola, MC and Stancampiano, L (personal communication) | 1.00 | 1.00 | 1.00 |
| **Fugazzola and Stancampiano 2012 B** | 2 | 1.5 | 1 | 2 | 0 | 1 | 3.5 | 4 | 0.88 | Fugazzola, MC and Stancampiano, L (personal communication) | 252.59 | 377.67 | 0.67 |
| **Fugazzola and Stancampiano 2012 C** | 2 | 1.5 | 1 | 2 | 0 | 1 | 3.5 | 4 | 0.88 | Fugazzola, MC and Stancampiano, L (personal communication) | 0.11 | 0.33 | 0.34 |
| **Fugazzola and Stancampiano 2012 D** | 2 | 1.5 | 1 | 2 | 0 | 1 | 3.5 | 4 | 0.88 | Fugazzola, MC and Stancampiano, L (personal communication) | 4.44 | 18.60 | 0.24 |
| **Fugazzola and Stancampiano 2012 E** | 2 | 1.5 | 1 | 2 | 0 | 1 | 3.5 | 4 | 0.88 | Fugazzola, MC and Stancampiano, L (personal communication) | 0.41 | 0.49 | 0.83 |
| **Fugazzola and Stancampiano 2012 F** | 2 | 1.5 | 1 | 2 | 0 | 1 | 3.5 | 4 | 0.88 | Fugazzola, MC and Stancampiano, L (personal communication) | 11.11 | 16.95 | 0.66 |
| **Fugazzola and Stancampiano 2012 G** | 2 | 1.5 | 1 | 2 | 0 | 1 | 3.5 | 4 | 0.88 | Fugazzola, MC and Stancampiano, L (personal communication) | 0.00 | 0.02 | 0.00 |
| **Fugazzola and Stancampiano 2012 H** | 2 | 1.5 | 1 | 2 | 0 | 1 | 3.5 | 4 | 0.88 | Fugazzola, MC and Stancampiano, L (personal communication) | 0.00 | 2.79 | 0.00 |
| **Hausfater and Watson 1976** | 1 | 2 | 2 | 1 | 0 | 1 | 3 | 4 | 0.75 | Alberts, SC; Archie, EA; Fitzpatrick, C; Tung, J (personal communication) | 29.96 | 22.33 | 1.34 |
| **Hernandez and MacIntosh 2009 A** | 1 | 2 | 2 | 1 | 0 | 0 | 3 | 3 | 1.00 | Hernandez, AD (personal communication) | NA | NA | NA |
| **Hernandez and MacIntosh 2009 B** | 1 | 2 | 2 | 1 | 0 | 0 | 3 | 3 | 1.00 | Hernandez, AD (personal communication) | NA | NA | NA |
| **Leclaire and Faulkner 2014 A** | 1.5 | 2.5 | 2.5 | 2 | 0 | 0 | 4 | 4.5 | 0.89 | Smyth, KN (personal communication) | 2.75 | 2.17 | 1.27 |
| **Leclaire and Faulkner 2014 B** | 1.5 | 2.5 | 2.5 | 2 | 0 | 0 | 4 | 4.5 | 0.89 | Smyth, KN (personal communication) | 2.25 | 1.33 | 1.69 |
| **Leclaire and Faulkner 2014 C** | 1.5 | 2.5 | 2.5 | 2 | 0 | 0 | 4 | 4.5 | 0.89 | Smyth, KN (personal communication) | 2.13 | 1.58 | 1.34 |
| **Leclaire and Faulkner 2014 D** | 1.5 | 2.5 | 2.5 | 2 | 0 | 0 | 4 | 4.5 | 0.89 | Smyth, KN (personal communication) | 0.38 | 0.38 | 1.00 |
| **Leclaire and Faulkner 2014 E** | 1.5 | 2.5 | 2.5 | 2 | 0 | 0 | 4 | 4.5 | 0.89 | Smyth, KN (personal communication) | 1.00 | 1.00 | 1.00 |
| **Leclaire and Faulkner 2014 F** | 1.5 | 2.5 | 2.5 | 2 | 0 | 0 | 4 | 4.5 | 0.89 | Smyth, KN (personal communication) | 0.75 | 0.71 | 1.06 |
| **Leclaire and Faulkner 2014 G** | 1.5 | 2.5 | 2.5 | 2 | 0 | 0 | 4 | 4.5 | 0.89 | Smyth, KN (personal communication) | 0.50 | 0.21 | 2.40 |
| **Leclaire and Faulkner 2014 H** | 1.5 | 2.5 | 2.5 | 2 | 0 | 0 | 4 | 4.5 | 0.89 | Smyth, KN (personal communication) | 0.13 | 0.13 | 1.00 |
| **Leclaire and Faulkner 2014 I** | 1.5 | 2.5 | 2.5 | 2 | 0 | 0 | 4 | 4.5 | 0.89 | Smyth, KN (personal communication) | 0.75 | 0.46 | 1.64 |
| **Leclaire and Faulkner 2014 J** | 1.5 | 2.5 | 2.5 | 2 | 0 | 0 | 4 | 4.5 | 0.89 | Smyth, KN (personal communication) | 0.75 | 0.46 | 1.64 |
| **MacIntosh et al. 2012 A** | 1 | 2 | 2 | 1 | 0 | 0 | 3 | 3 | 1.00 | Hernandez, AD (personal communication) | 1.50 | 1.02 | 1.47 |
| **MacIntosh et al. 2012 B** | 1 | 2 | 2 | 1 | 0 | 0 | 3 | 3 | 1.00 | Hernandez, AD (personal communication) | 1.00 | 0.75 | 1.34 |
| **MacIntosh et al. 2012 C** | 1 | 2 | 2 | 1 | 0 | 0 | 3 | 3 | 1.00 | Hernandez, AD (personal communication) | 366.00 | 235.00 | 1.56 |
| **Meade 1984 A** | 1 | 2 | 2 | 1 | 0 | 1 | 3 | 4 | 0.75 | Alberts, SC; Archie, EA; Fitzpatrick, C; Tung, J (personal communication) | 4.10 | 2.59 | 1.58 |
| **Meade 1984 B** | 1 | 2 | 2 | 1 | 0 | 1 | 3 | 4 | 0.75 | Alberts, SC; Archie, EA; Fitzpatrick, C; Tung, J (personal communication) | 3.60 | 1.96 | 1.84 |
| **Müller-Graf et al. 1996 A** | 1 | 2 | 2 | 2 | 0 | 0 | 3 | 4 | 0.75 | Doellman, M.; Habig, B.; Olansen, J; Woods, K (citation: ^36^) | NA | NA | NA |
| **Müller-Graf et al. 1996 B** | 1 | 2 | 2 | 2 | 0 | 0 | 3 | 4 | 0.75 | Doellman, M.; Habig, B.; Olansen, J; Woods, K (citation: ^36^) | NA | NA | NA |
| **Ravasi 2009 A** | 1 | 2 | 2 | 2 | 0 | 1 | 3 | 5 | 0.60 | Ravasi, D. (personal communication) | 0.83 | 0.17 | 4.97 |
| **Ravasi 2009 B** | 1 | 2 | 2 | 2 | 0 | 1 | 3 | 5 | 0.60 | Ravasi, D. (personal communication) | 0.33 | 0.50 | 0.66 |
| **Ravasi 2009 C** | 1 | 2 | 2 | 2 | 0 | 1 | 3 | 5 | 0.60 | Ravasi, D. (personal communication) | 0.00 | 0.50 | 0.00 |
| **Ravasi 2009 D** | 1 | 2 | 2 | 2 | 0 | 1 | 3 | 5 | 0.60 | Ravasi, D. (personal communication) | 0.17 | 0.17 | 1.00 |
| **Ravasi 2009 E** | 1 | 2 | 2 | 2 | 0 | 1 | 3 | 5 | 0.60 | Ravasi, D. (personal communication) | 0.97 | 0.67 | 1.45 |
| **Ravasi 2009 F** | 1 | 2 | 2 | 2 | 0 | 1 | 3 | 5 | 0.60 | Ravasi, D. (personal communication) | 0.33 | 0.5 | 0.66 |
| **Ravasi 2009 G** | 1 | 2 | 2 | 2 | 0 | 1 | 3 | 5 | 0.60 | Ravasi, D. (personal communication) | 1.00 | 0.67 | 1.49 |
| **Ravasi 2009 H** | 1 | 2 | 2 | 2 | 0 | 1 | 3 | 5 | 0.60 | Ravasi, D. (personal communication) | 0.50 | 0.67 | 0.75 |
| **Ravasi 2009 I** | 1 | 2 | 2 | 2 | 0 | 1 | 3 | 5 | 0.60 | Ravasi, D. (personal communication) | 0.17 | 0.33 | 0.51 |
| **Setchell et al. 2007 A** | 1 | 1.5 | 2 | 1 | 0 | 0.5 | 2.5 | 3.5 | 0.71 | Setchell, J. (personal communication) | NA | NA | NA |
| **Setchell et al. 2007 B** | 1 | 1.5 | 2 | 1 | 0 | 0.5 | 2.5 | 3.5 | 0.71 | Setchell, J. (personal communication) | NA | NA | NA |
| **Setchell et al. 2007 C** | 1 | 1.5 | 2 | 1 | 0 | 0.5 | 2.5 | 3.5 | 0.71 | Setchell, J. (personal communication) | NA | NA | NA |
| **Setchell et al. 2007 D** | 1 | 1.5 | 2 | 1 | 0 | 0.5 | 2.5 | 3.5 | 0.71 | Setchell, J. (personal communication) | NA | NA | NA |
| **Setchell et al. 2007 E** | 1 | 1.5 | 2 | 1 | 0 | 0.5 | 2.5 | 3.5 | 0.71 | Setchell, J. (personal communication) | NA | NA | NA |
| **Setchell et al. 2007 F** | 1 | 1.5 | 2 | 1 | 0 | 0.5 | 2.5 | 3.5 | 0.71 | Setchell, J. (personal communication) | NA | NA | NA |
| **Setchell et al. 2007 G** |  | 1.5 | 2 | 1 | 0 | 0.5 | 2.5 | 3.5 | 0.71 | Setchell, J. (personal communication) | NA | NA | NA |
| **Setchell et al. 2007 H** | 1 | 1.5 | 2 | 1 | 0 | 0.5 | 2.5 | 3.5 | 0.71 | Setchell, J. (personal communication) | NA | NA | NA |
| **Smyth and Drea 2015 A** | 1.5 | 2.5 | 2.5 | 2 | 0 | 0 | 4 | 4.5 | 0.89 | Smyth, KN (personal communication) | 4.23 | 2.5 | 1.69 |
| **Smyth and Drea 2015 B** | 1.5 | 2.5 | 2.5 | 2 | 0 | 0 | 4 | 4.5 | 0.89 | Smyth, KN (personal communication) | 0.93 | 0.78 | 1.19 |
| **Smyth and Drea 2015 C** | 1.5 | 2.5 | 2.5 | 2 | 0 | 0 | 4 | 4.5 | 0.89 | Smyth, KN (personal communication) | 0.6 | 0.32 | 1.88 |
| **Smyth and Drea 2015 D** | 1.5 | 2.5 | 2.5 | 2 | 0 | 0 | 4 | 4.5 | 0.89 | Smyth, KN (personal communication) | 0.63 | 0.15 | 4.20 |
| **Smyth and Drea 2015 E** | 1.5 | 2.5 | 2.5 | 2 | 0 | 0 | 4 | 4.5 | 0.89 | Smyth, KN (personal communication) | 0.83 | 0.43 | 1.93 |
| **Smyth and Drea 2015 F** | 1.5 | 2.5 | 2.5 | 2 | 0 | 0 | 4 | 4.5 | 0.89 | Smyth, KN (personal communication) | 0.37 | 0.15 | 2.47 |
| **Smyth and Drea 2015 G** | 1.5 | 2.5 | 2.5 | 2 | 0 | 0 | 4 | 4.5 | 0.89 | Smyth, KN (personal communication) | 0.87 | 0.77 | 1.13 |
| **Ungerfeld and Correa, 2007** | 2 | 2.375 | 2.5 | 1.5 | 0 | 0.75 | 4.375 | 4.75 | 0.91 | Doellman, M.; Habig, B; Olansen, J; Woods, K (citation: ^54^) | 965.5 | 1360 | 0.71 |

**Table S12.** Quantifying allostatic load in dominant and subordinate males and females (adopted from Goymann and Wingfield, 2004 ^55^).

| **Allostatic load model:** Differences in the cumulative physiological burdens exerted on the body to meet life history demands predict status-related variation in parasitism. Specifically, within a species and sex, the rank that experiences the highest allostatic load will exhibit the highest parasitism. When ranks do not differ in allostatic load, there will be no difference in parasitism between ranks. | | |
| --- | --- | --- |
| **quantifying allostatic load in dominant males and females** | | |
|  | **allostatic load** | **criteria** |
| **rank acquisition** | 1 (low) | through inheritance  via formation of a breeding pair with helpers  via social queuing |
|  | 2 (moderate) | via low-level threat/overt aggression |
|  | **allostatic load** | **criteria** |
| **rank maintenance** | 1 (low) | without aggression or via a queuing convention in which subordinates do not typically challenge dominants |
|  | 2 (moderate) | via low-level psychological threat with dominants controlling access to resources |
|  | 3 (high) | via overt aggression in a hierarchy in which dominants are frequently challenged by subordinates; typically social units with non-linear hierarchies |
| **quantifying allostatic load in subordinate males and females** | | |
|  | **allostatic load** | **criteria** |
| **degree of threat** | 1 (low) | rarely receive threats from dominants |
|  | 2 (high) | regularly receive low-level physical or psychological aggression from dominants |
|  | 3 (high) | regularly receive high-level aggression and intimidation from dominants |
|  | **allostatic load** | **criteria** |
| **outlets for threats from dominants** | 1 (low) | outlets are available  options to avoid being threatened by dominants (e.g. fusion-fission events or units with strong social support) |
|  | 2 (moderate) | Cohesive groups |
| **quantifying allostatic load in subordinate males and females** | | |
|  | **allostatic load** | **criteria** |
| **food resource control and availability** | 0 (low to moderate) | resource control does not differ between dominants and subordinates |
|  | 1 (high) | resource control substantially greater in dominants than subordinates (add 1 to subordinate allostatic load score) |

**Table S13.** Topology and divergence times extracted from primary literature. Nodes correspond to Fig. S3 for male studies and Fig. S5 for female studies.

| **male studies** | | |
| --- | --- | --- |
| **node** | **age (My)** | **reference** |
| 1 | 432.4 | dos Reis et al. 2015 ^59^ |
| 2 | 324.75 | dos Reis et al. 2015 ^59^ |
| 3 | 280.0 | Pyron 2010, Mulcahy et al. 2012, Zheng and Wiens 2016 ^60-62^ |
| 4 | 161.55 | Mulcahy et al. 2012 ^61^ |
| 5 | 99.0 | Bininda-Emonds et al. 2007, Arnason et al. 2008 ^63,64^ |
| 6 | 74.1 | Pozzi et al. 2014 ^65^ |
| 7 | 72.7 | dos Reis et al. 2012 ^66^ |
| 8 | 69.9 | dos Reis et al. 2012 ^66^ |
| 9 | 64.9 | Nyakatura and Bininda-Emonds 2012 ^67^ |
| 10 | 57 | Ericson et al. 2014 ^68^ |
| 11 | 53 | Ericson et al. 2014 ^68^ |
| 12 | 43.6 | Ericson et al. 2014 ^68^ |
| 13 | 32.1 | Perelman et al. 2011, Pozzi et al. 2014 ^65,69^ |
| 14 | 24.6 | Ericson et al. 2014 ^68^ |
| 15 | 20.8 | Pozzi et al. 2014 ^65^ |
| 16 | 14.7 | Bibi 2013 ^70^ |
| 17 | 3.24 | Perelman et al. 2011 ^69,71^ |
| 18 | 1.6 | Ting 2008 ^71^ |
| 19 | 0.35 | Zinner et al. 2013 ^72^ |
| 20 | undated node, fixed equidistant between neighboring dated nodes and/or tips by BLADG | |
| **female studies** | | |
| **node** | **age (My)** | **reference** |
| 1 | 99.0 | Bininda-Emonds et al. 2007, Arnason et al. 2008 ^63,64^ |
| 2 | 72.7 | dos Reis et al. 2012 ^66^ |
| 3 | 69.9 | dos Reis et al. 2012 ^66^ |
| 4 | 14.09 | Pozzi et al. 2014 ^65^ |
| 5 | 12.17 | Perelman et al. 2011 ^69^ |
| 6 | 6.67 | Perelman et al. 2011 ^69^ |
| 7 | 2.085 | Zinner et al. 2013 ^72^ |
| 8 | 0.9 | Perelman et al. 2011 ^69^ |
| 9 | 0.35 | Zinner et al. 2013 ^72^ |

**Supplementary Figures**

**Figure S1.** Funnel plot comparing male social status and parasitism incorporating the trim and fill method (Egger’s test: p<0.001). The observed outcome (Cohen’s *d*) is represented on the x-axis and the standard error is represented on the y-axis. The white circle indicates the one fewer published analysis than expected with a small sample size and where subordinates had higher parasitism than dominants.

**Figure S2.** Phylogeny of male species included in the meta-analysis.

**Figure S3.** Funnel plot for tests comparing female social status and parasitism. An Egger’s test showed no significant publication bias (Egger’s test: p= 0.118). The observed outcome (Cohen’s *d*) is represented on the x-axis and the standard error is represented on the y-axis.

**Figure S4.** Phylogeny of female species included in the meta-analysis.

**Figure S5.** Pearson product-moment correlation showing no significant correlation between relative allostatic load and relative parasitism in analyses of male vertebrates (r=-0.014; p=0.922; n=50).

**Figure S6.** Pearson product-moment correlation showing no significant correlation between relative allostatic load and relative parasitism in analyses of female vertebrates (r= -0.038, p= 0.802; n=48).

**Figure S7. P**referred **R**eporting**I**tems for **S**ystematic Reviews and **M**eta-**A**nalyses (PRISMA)

**Figure S1.** Funnel plot comparing male social status and parasitism incorporating the trim and fill method (Egger’s test: p<0.001). The observed outcome (Cohen’s *d*) is represented on the x-axis and the standard error is represented on the y-axis. The white circle indicates the one fewer published analysis than expected with a small sample size and where subordinates had higher parasitism than dominants.

**
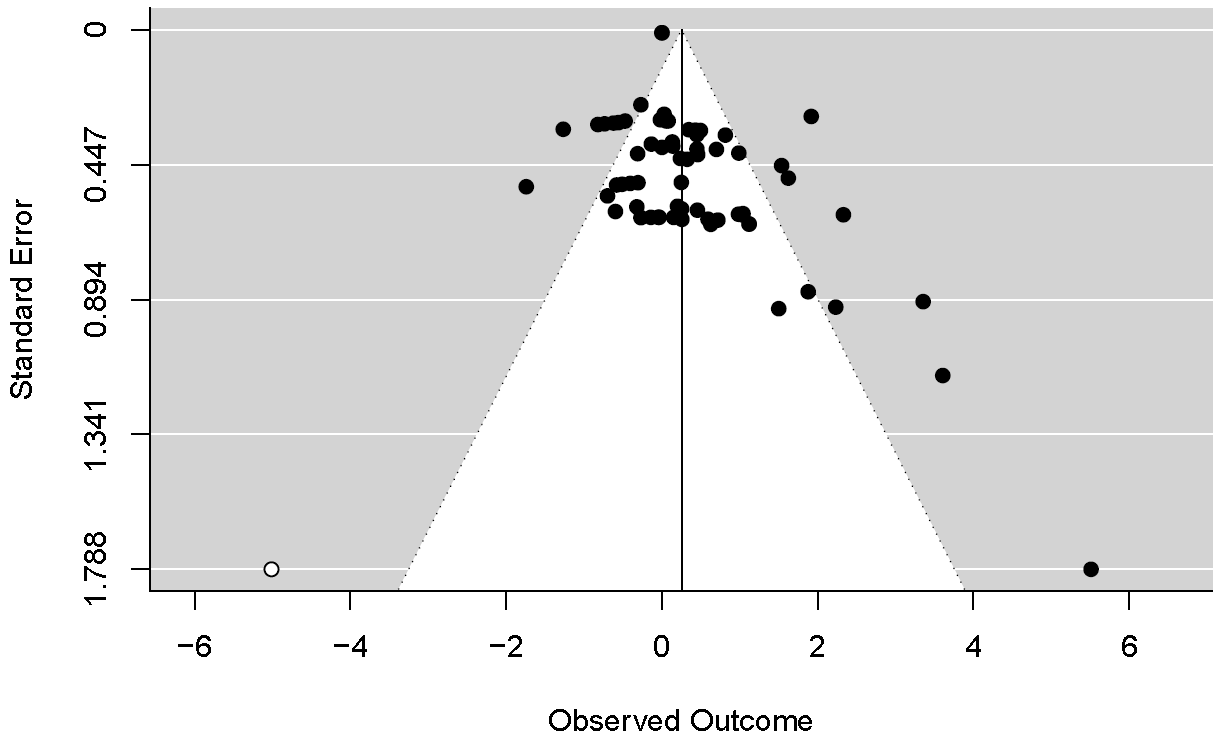
**

**Figure S2**. Phylogeny of male species included in the meta-analysis.


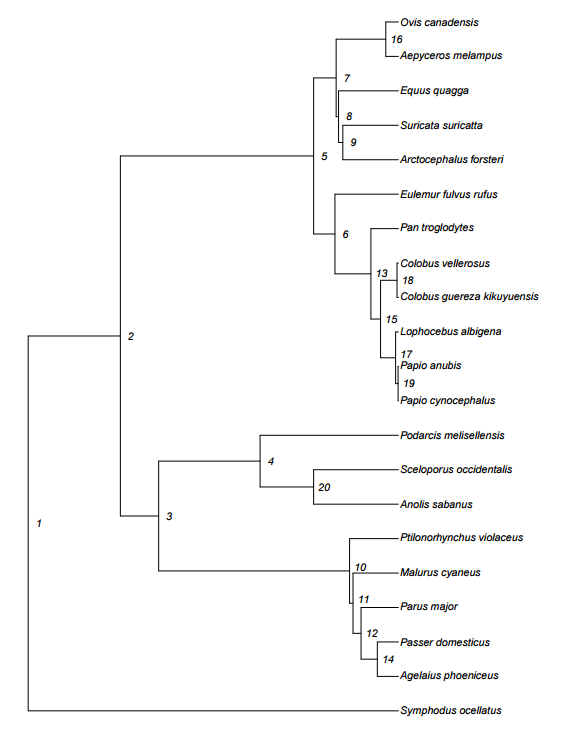


**Figure S3.** Funnel plot for tests comparing female social status and parasitism. An Egger’s test showed no significant publication bias (Egger’s test: p= 0.118). The observed outcome (Cohen’s *d*) is represented on the x-axis and the standard error is represented on the y-axis.

**
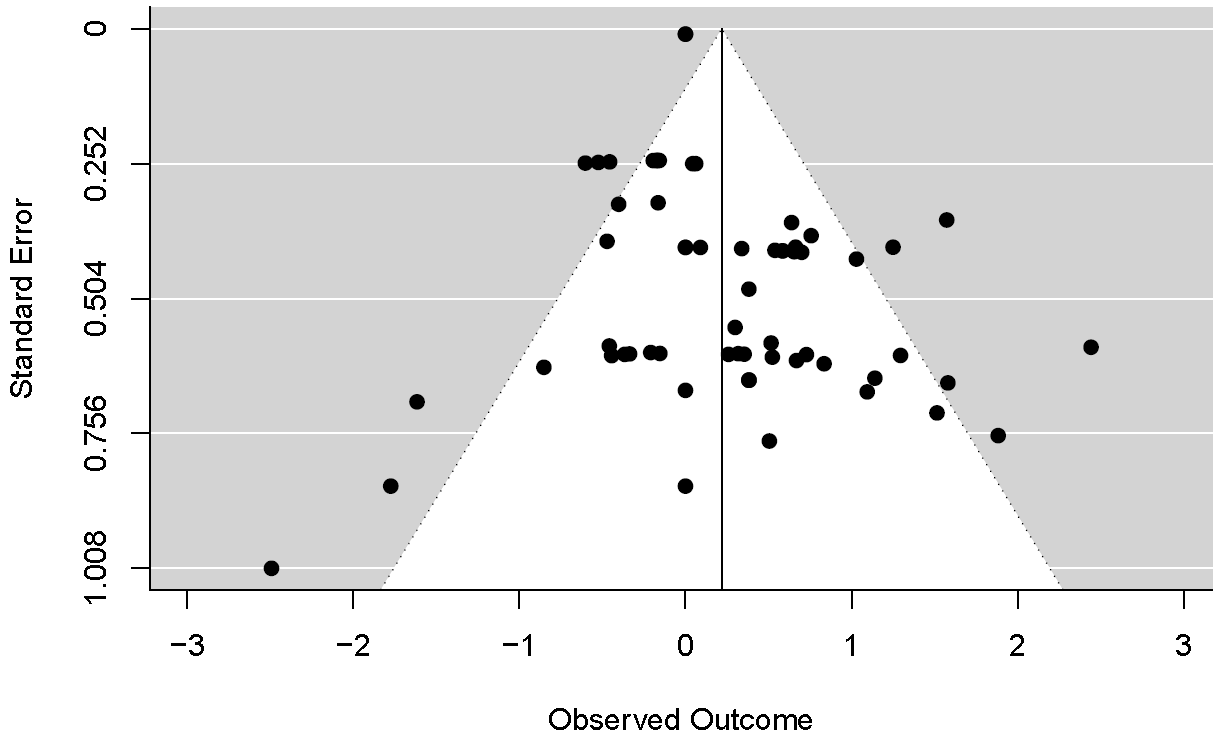
**

**Figure S4.** Phylogeny of female species included in the meta-analysis.


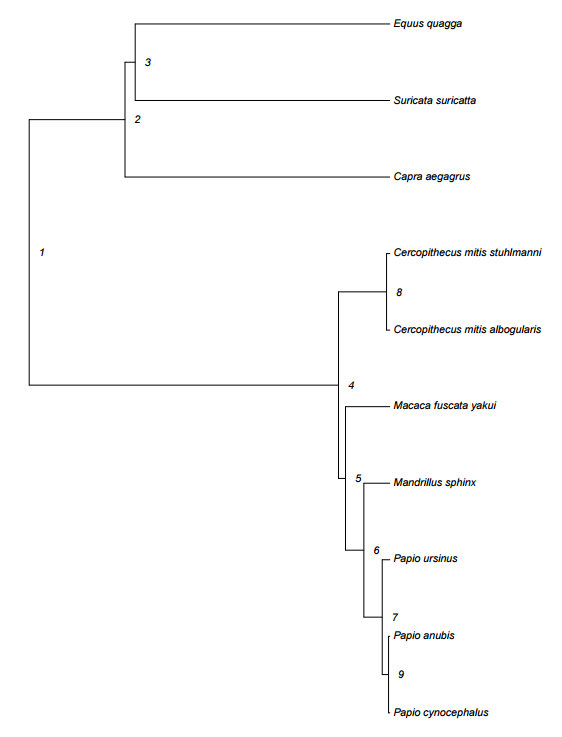


**Figure S5.** Pearson product-moment correlation showing no significant correlation between relative allostatic load and relative parasitism in analyses of male vertebrates (r=-0.014; p=0.922; n=50).

**
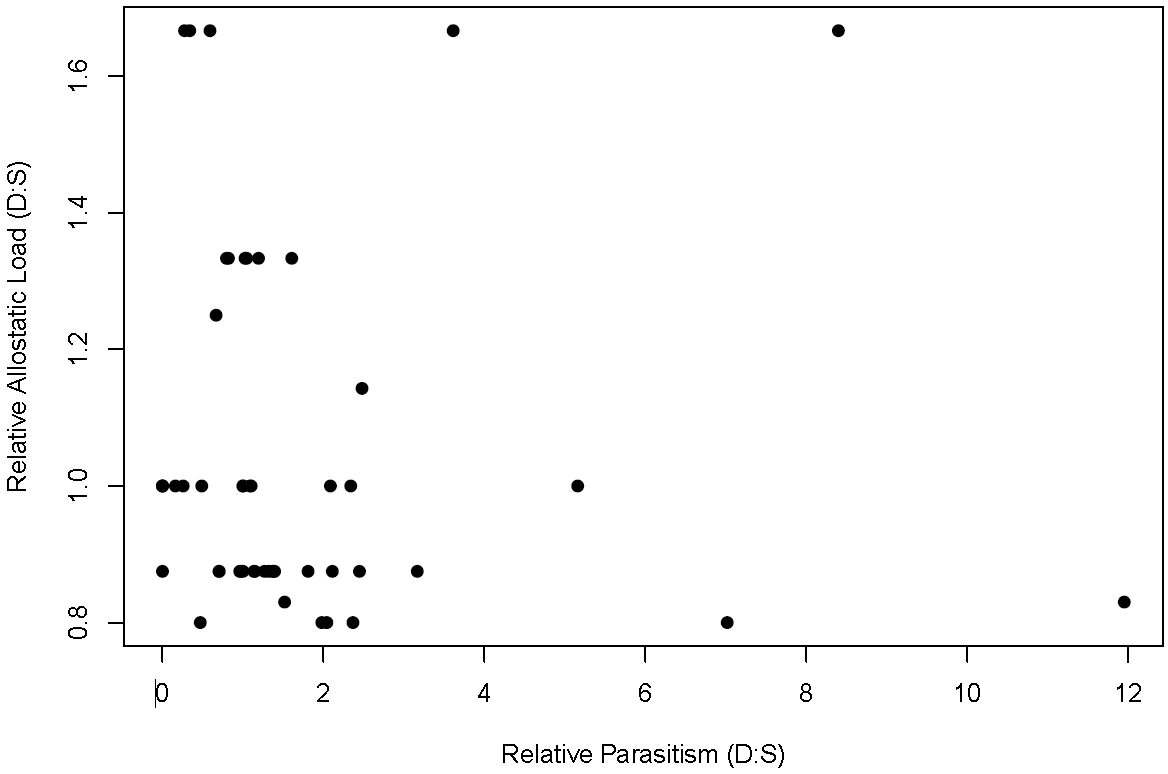
**

**Figure S6.** Pearson product-moment correlation showing no significant correlation between relative allostatic load and relative parasitism in analyses of female vertebrates (r= -0.038, p= 0.802; n=48).

**
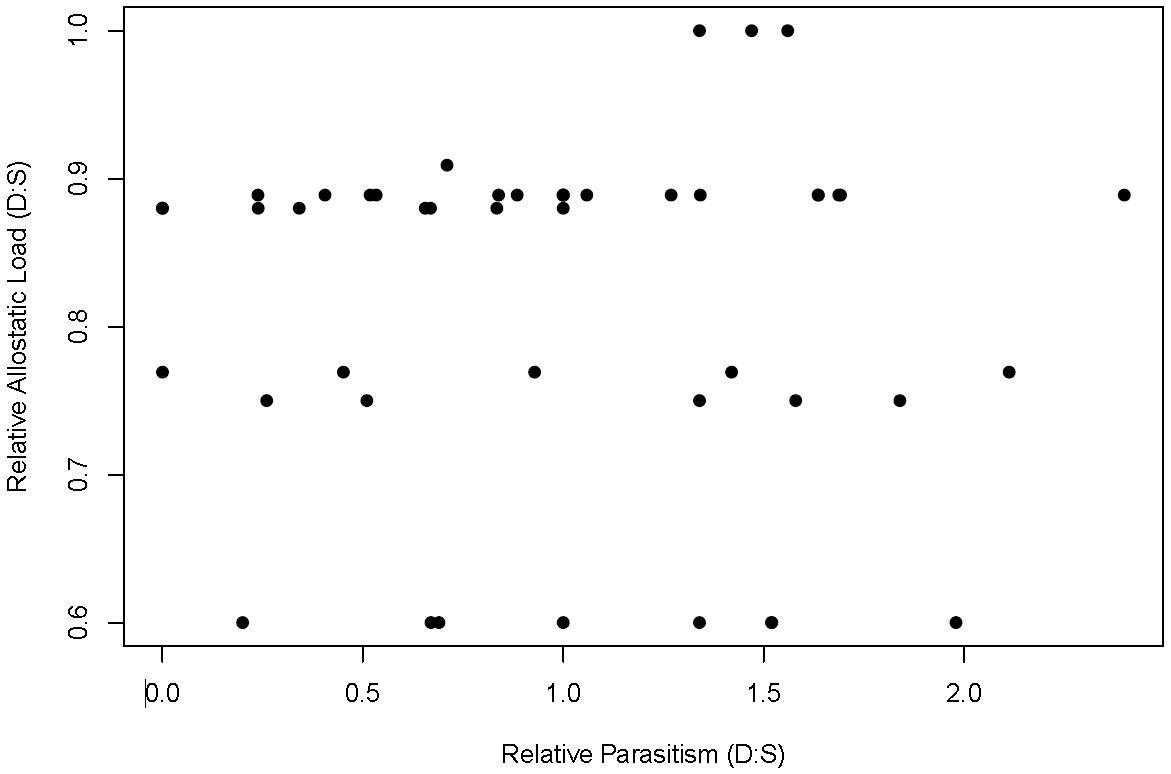
**

**Figure S7. P**referred **R**eporting**I**tems for **S**ystematic Reviews and **M**eta-**A**nalyses (PRISMA)

**
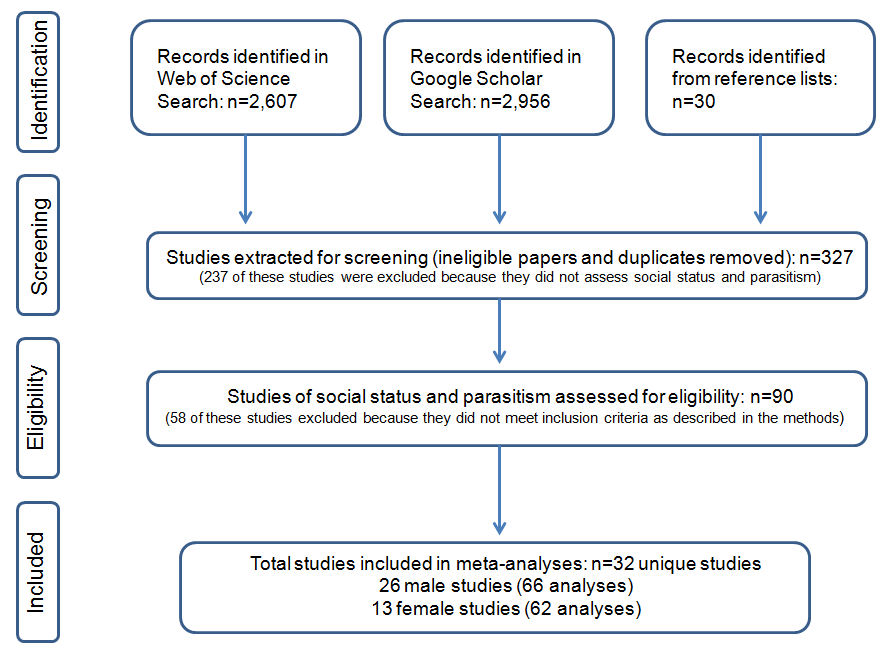
**

**References**

1 Archie, E. A., Morrison, T. A., Foley, C. A. H., Moss, C. J. & Alberts, S. C. Dominance rank relationships among wild female African elephants, *Loxodonta africana*. *Anim. Behav.* **71**, 117-127, doi:10.1016/j.anbehav.2005.03.023 (2006).

2 Fuentes, A. Social systems and socioecology: understanding the evolution of primate behavior in *Primates in perspective* Vol. 2 (eds. Campbell C. J., Fuentes, A., MacKinnon K. C., Bearder S.K., Stumpf R.M.) 500-511 (Oxford, 2011).

3 Cords, M. The behavior, ecology and social evolution of Cercopithecine monkeys in *The evolution of primate societies* (eds. Mitani J. C., Call, J., Kappeler P. M., Palombit R. A., Silk J. B.) 91-112 (University of Chicago, 2012).

4 Arlet, M. E., Molleman, F. & Chapman, C. A. Mating tactics in male grey‐cheeked mangabeys (*Lophocebus albigena*). *Ethology* **114**, 851-862, doi:10.1111/j.1439-0310.2008.01533.x (2008).

5 Bartoli, P., Morand, S., Riutort, J. J. & Combes, C. Acquisition of parasites correlated with social rank and behavioural changes in a fish species. *J. Helminthol.* **74**, 289-293 (2000).

6 Borgia, G. & Collis, K. Parasites and bright male plumage in the satin bowerbird (*Ptilonorhynchus violaceus*). *Am. Zool.* **30**, 279-285 (1990).

7 Caine, J. & Melfi, V. A longitudinal study into the factors affecting the prevalence and intensity of *Trichuris trichiura* in captive *Colobus guereza*. *Ratel* **33**, 5-10 (2006).

8 Clough, D., Heistermann, M. & Kappeler, P. M. Host intrinsic determinants and potential consequences of parasite infection in free-ranging red-fronted lemurs (*Eulemur fulvus rufus*). *Am. J. Phys. Anthropol.* **142**, 441-452, doi:10.1002/ajpa.21243 (2010).

9 Colombelli-Negrel, D. & Kleindorfer, S. In superb fairy wrens (*Malurus cyaneus*), nuptial males have more blood parasites and higher haemoglobin concentration than eclipsed males. *Aust. J. Zool.* **56**, 117-121, doi:10.1071/zo07072 (2008).

10 Cockburn, A. & Double, M. C. Cooperatively breeding superb fairy wrens show no facultative manipulation of offspring sex ratio despite plausible benefits. *Behav. Ecol. Sociobiol.* **62**, 681-688, doi:10.1007/s00265-007-0492-1 (2008).

11 Dufva, R. & Allander, K. Intraspecific variation in plumage coloration reflects immune response in great tit (*Parsus major*). *Funct. Ecol.* **9**, 785-789, doi:10.2307/2390253 (1995).

12 Fugazzola, M. C. & Stancampiano, L. Host social rank and parasites: plains zebra (*Equus quagga*) and intestinal helminths in Uganda. *Vet. Parasitol.* **188**, 115-119, doi:10.1016/j.vetpar.2012.03.019 (2012).

13 Huyghe, K. *et al.* Relationships between hormones, physiological performance and immunocompetence in a color-polymorphic lizard species, *Podarcis melisellensis*. *Horm. Behav.* **55**, 488-494, doi:10.1016/j.yhbeh.2009.02.005 (2009).

14 Melfi, V. & Poyser, F. Trichuris burdens in zoo-housed *Colobus guereza*. *Int. J. Primatol.* **28**, 1449-1456, doi:10.1007/s10764-007-9206-9 (2007).

15 Mooring, M. S., McKenzie, A. A. & Hart, B. L. Role of sex and breeding status in grooming and total tick load of impala. *Behav. Ecol. Sociobiol.* **39**, 259-266, doi:10.1007/s002650050288 (1996).

16 Jarman, P. Mating system and sexual dimorphism in large terrestrial, mammalian herbivores. *Biol. Rev.* **58**, 485-520 (Biological Reviews, 1983).

17 Muehlenbein, M. P. & Watts, D. P. The costs of dominance: testosterone, cortisol and intestinal parasites in wild male chimpanzees. *Biopsychosoc. Med.* **4**, 21-21, doi:10.1186/1751-0759-4-21 (2010).

18 Negro, S. S., Caudron, A. K., Dubois, M., Delahaut, P. & Gemmell, N. J. Correlation between male social status, testosterone levels, and parasitism in a dimorphic polygynous mammal. *PLoS One* **5**, doi:10.1371/journal.pone.0012507 (2010).

19 Pelletier, F., Page, K. A., Ostiguy, T. & Festa-Bianchet, M. Fecal counts of lungworm larvae and reproductive effort in bighorn sheep, *Ovis canadensis*. *Oikos* **110**, 473-480, doi:10.1111/j.0030-1299.2005.14120.x (2005).

20 Festa-Bianchet, M. The social system of bighorn sheep: grouping patterns, kinship and female dominance rank. *Anim. Behav.* **42,** 71-82 (1991).

21 Poiani, A., Goldsmith, A. R. & Evans, M. R. Ectoparasites of house sparrows (*Passer domesticus*): an experimental test of the immunocompetence handicap hypothesis and a new model. *Behav. Ecol. Sociobiol.* **47**, 230-242, doi:10.1007/s002650050660 (2000).

22 Schall, J. J. & Houle, P. R. Malarial parasitism and home range and social status of male western fence lizards, *Sceloporus-occidentalis*.*J. Herpetol.* **26**, 74-76, doi:10.2307/1565026 (1992).

23 Ferguson, G. W. & Brockman, T. Geographic differences of growth rate of *Sceloporus* lizards (Sauria, Iguanidae). *Copeia*, 259-264, doi:10.2307/1444002 (1980).

24 Schall, J. J. & Staats, C. M. Virulence of lizard malaria: three species of *Plasmodium* infecting *Anolis sabanus*, the endemic anole of Saba, Netherlands Antilles. *Copeia*, 39-43 (2002).

25 Thorpe, R. S., Surget-Groba, Y. & Johansson, H. The relative importance of ecology and geographic isolation for speciation in anoles. *Philos. Trans. Roy. Soc. B* **363**, 3071-3081, doi:10.1098/rstb.2008.0077 (2008).

26 Smyth, K. N. & Drea, C. M. Patterns of parasitism in the cooperatively breeding meerkat: a cost of dominance for females. *Behav. Ecol.* **27**, 148-157, doi: 10.1093/beheco/arv132 (2015).

27 Teichroeb, J. A., Kutz, S. J., Parkar, U., Thompson, R. C. A. & Sicotte, P. Ecology of the gastrointestinal parasites of *Colobus vellerosus* at Boabeng-Fiema, Ghana: possible anthropozoonotic transmission. *Am. J. Phys. Anthropol.* **140**, 498-507, doi:10.1002/ajpa.21098 (2009).

28 Walker S. "Colobus vellerosus". *Animal Diversity Web* 2009 animaldiversity.org (2017).

29 Leclaire, S. & Faulkner, C. T. Gastrointestinal parasites in relation to host traits and group factors in wild meerkats *Suricata suricatta*. *Parasitology* **141**, 925-933, doi:10.1017/s0031182013002333 (2014).

30 Weatherhead, P. J. Secondary sexual traits, parasites, and polygyny in red- winged blackbirds, *Agelaius phoeniceus*. *Behav. Ecol.* **1**, 125-130, doi:10.1093/beheco/1.2.125 (1990).

31 Jaffe, K. E. & Isbell, L. A. The guenons: polyspecific associations in socioecological perspective in *Primates in perspective* (eds. Campbell C. J., Fuentes, A., MacKinnon K. C., Bearder S.K., Stumpf R.M.) 277-300 (Oxford, 2011).

32 Hernandez, A. D., MacIntosh, A. J. & Huffman, M. A. Primate parasite ecology: patterns and predictions from an on-going study of Japanese macaques in *Primate parasite ecology: the dynamics and study of host-parasite relationships* (eds. Huffman M. A., Chapman C. A.) 387-402 (Cambridge, 2009).

33 Setchell, J. M. *et al.* Parasite prevalence, abundance, and diversity in a semi-free-ranging colony of *Mandrillus sphinx*. *Int. J. Primatol.* **28**, 1345-1362, doi:10.1007/s10764-007-9225-6 (2007).

34 Cote, S. D. & Festa-Bianchet, M. Reproductive success in female mountain goats: the influence of age and social rank. *Anim. Behav.* **62**, 173-181, doi:10.1006/anbe.2001.1719 (2001).

35 Akinyi, M. Y. *et al.* Role of grooming in reducing tick load in wild baboons (*Papio cynocephalus*). *Anim. Behav.* **85**, 559-568, doi:10.1016/j.anbehav.2012.12.012 (2013).

36 Müller Graf, C. D. M., Collins, D. A. & Woolhouse, M. E. J. Intestinal parasite burden in five troops of olive baboons (*Papio cynocephalus anubis*) in Gombe Stream National Park, Tanzania. *Parasitology* **112**, 489-497 (1996).

37 Irwin, M. T. & Raharison, J. L. A review of the endoparasites of the lemurs of Madagascar. *Malagasy Nat.* **2**, 66-93 (2009).

38 Martin, S. K. Behavioral and environmental correlates of parasite burden in *Eulemur cinereiceps* from southeastern Madagascar. (Doctoral dissertation, State University of New York at Stony Brook, 2011).

39 Roberts, L. S., Janovy, J., Schmidt, G. & Larry, S. Roberts' Foundations of Parasitology (McGraw Hill, 2009).

40 Ferraguti, M., Martinez-de la Puente, J., Ruiz, S., Soriguer, R. & Figuerola, J. On the study of the transmission networks of blood parasites from SW Spain: diversity of avian haemosporidians in the biting midge *Culicoides circumscriptus* and wild birds. *Parasit. Vectors* **6**, doi:10.1186/1756-3305-6-208 (2013).

41 Poulin, R. Group-living and infestation by ectoparasites in passerines. *Condor* **93**, 418-423, doi:10.2307/1368958 (1991).

42 Kabilov, T. K. On the life cycle of the nematode *Abbreviata kazachstanica*. *Parazitologiya* **14**, 263-270 (1980).

43 Brumpt, T. & Joyeux, C. Sur un infusoire nouveau parasite du chimpanzé, *Troglodytella abrassarti ngn* sp. Bull Soc Pathol Exot. **5**, 499-503. (1912).

44 Roberts L. S., Janovy J., Schmidt G. D. Foundations of parasitology. (McGraw Hill, 2005).

45 Aucott, J. N. & Ravdin, J. I. Amebiasis and nonpathogenic intestinal protozoa. *Infect. Dis. Clin. North Am.* **7**, 467-485 (1993).

46 Anderson, R. C. Nematode parasites of vertebrates: their development and transmission. (CABI, 2000).

47 Olsen, O. W. Animal parasites: their life cycles and ecology. (University Park, 1974).

48 Muller, R. Worms and human disease (2^nd^ edition). (CABI, 2002).

49 Crompton, D. W. T. & Savioli, L. Handbook of helminthiasis for public health. (CRC, 2006).

50 Goncalves, M. L. C., Araujo, A. & Ferreira, L. F. Human intestinal parasites in the past: new findings and a review. *Memorias do Instituto Oswaldo Cruz* **98**, 103-118 (2003).

51 Weatherhead, P. J., Metz, K. J., Bennett, G. F. & Irwin, R. E. Parasite faunas, testosterone, and secondary sexual traits in male red-winged blackbirds. *Behav. Ecol. Sociobiol.* **33**, 13-23, doi:10.1007/bf00164342 (1993).

52 Smyth, J. D. Introduction to animal parasitology. (Cambridge University, 1994).

53 Centers for Disease Control. *Parasites - nonpathogenic (harmless) intestinal protozoa*. <http://www.cdc.gov/parasites/nonpathprotozoa/biology.html2015> (2015).

54 Ungerfeld, R. & Correa, O. Social dominance of female dairy goats influences the dynamics of gastrointestinal parasite eggs. *Appl. Anim. Behav. Sci.* **105**, 249-253, doi:10.1016/j.applanim.2006.05.008 (2007).

55 Goymann, W. & Wingfield, J. C. Allostatic load, social status and stress hormones: the costs of social status matter. *Anim. Behav.* **67**, 591-602, doi:10.1016/j.anbehav.2003.08.007 (2004).

56 Mitani, J. C., Watts, D. P. & Muller, M. N. Recent developments in the study of wild chimpanzee behavior. *Evol. Anthropol.* **11**, 9-25, doi:10.1002/evan.10008 (2002).

57 Sapolsky, R. M. Endocrinology alfresco - psychoendocrine studies of wild baboons. *Recent Prog. Horm. Res.* **48**, 437-468 (1993).

58 Beletsky, L. & Orians, G. Territoriality among male red-winged blackbirds. *Behav. Ecol. Sociobiol.* **20**, 21-34, doi:10.1007/BF00292163 (1987).

59 dos Reis, M. *et al.* Uncertainty in the timing of origin of animals and the limits of precision in molecular timescales. *Curr. Biol.* **25**, 2939-2950, doi:10.1016/j.cub.2015.09.066 (2015).

60 Pyron, R. A. A likelihood method for assessing molecular divergence time estimates and the placement of fossil calibrations. *Syst. Biol.* **59**, 185-194, doi:10.1093/sysbio/syp090 (2010).

61 Mulcahy, D. G. *et al.* Estimating divergence dates and evaluating dating methods using phylogenomic and mitochondrial data in squamate reptiles. *Mol. Phylogenet. Evol.* **65**, 974-991, doi:10.1016/j.ympev.2012.08.018 (2012).

62 Zheng, Y. & Wiens, J. J. Combining phylogenomic and supermatrix approaches, and a time-calibrated phylogeny for squamate reptiles (lizards and snakes) based on 52 genes and 4162 species. *Mol. Phylogenet. Evol.* **94**, 537-547, doi:10.1016/j.ympev.2015.10.009 (2016).

63 Bininda-Emonds, O. R. P. *et al.* The delayed rise of present-day mammals. *Nature* **446**, 507-511, doi:10.1038/nature07347 (2007).

64 Arnason, U. *et al.* Mitogenomic relationships of placental mammals and molecular estimates of their divergences. *Gene* **421**, 37-51, doi:10.1016/j.gene.2008.05.024 (2008).

65 Pozzi, L. *et al.* Primate phylogenetic relationships and divergence dates inferred from complete mitochondrial genomes. *Mol. Phylogenet. Evol.* **75**, 165-183, doi:10.1016/j.ympev.2014.02.023 (2014).

66 dos Reis, M. *et al.* Phylogenomic datasets provide both precision and accuracy in estimating the timescale of placental mammal phylogeny. *Proc. Roy. Soc. B* **279**, 3491-3500, doi:10.1098/rspb.2012.0683 (2012).

67 Nyakatura, K. & Bininda-Emonds, O. R. P. Updating the evolutionary history of Carnivora (Mammalia): a new species-level supertree complete with divergence time estimates. *BMC Biol.* **10**, 12, doi:10.1186/1741-7007-10-12 (2012).

68 Ericson, P. G. P., Klopfstein, S., Irestedt, M., Nguyen, J. M. T. & Nylander, J. A. A. Dating the diversification of the major lineages of Passeriformes (Aves). *BMC Evol. Biol.* **14**, 8, doi:10.1186/1471-2148-14-8 (2014).

69 Perelman, P. *et al.* A molecular phylogeny of living primates (Primate phylogeny). *PLoS Genet.* **7**, e1001342, doi:10.1371/journal.pgen.1001342 (2011).

70 Bibi, F. A multi-calibrated mitochondrial phylogeny of extant Bovidae (Artiodactyla, Ruminantia) and the importance of the fossil record to systematics. *BMC Evol. Biol.* **13**, 166, doi:10.1186/1471-2148-13-166 (2013).

71 Ting, N. Mitochondrial relationships and divergence dates of the African colobines: evidence of Miocene origins for the living colobus monkeys. *J. Hum. Evol.* **55**, 312-325, doi:10.1016/j.jhevol.2008.02.011 (2008).

72 Zinner, D., Wertheimer, J., Liedigk, R., Groeneveld, L. F. & Roos, C. Baboon phylogeny as inferred from complete mitochondrial genomes. *Am. J. Phys. Anthropol.* **150**, 133-140, doi:10.1002/ajpa.22185 (2013).
