# Supplementary material for: Social status and parasitism in male and female vertebrates: a meta-analysis
Source: Sci Rep. 2018 Feb 26;8:3629. doi: 10.1038/s41598-018-21994-7 (PMC5827031; doi:10.1038/s41598-018-21994-7)
Supplement: Supplementary file 2 — Supplementary Code [file 41598_2018_21994_MOESM2_ESM.pdf]

Supplementary materials (sample code) for

Social status and parasitism in male and female vertebrates: a meta-analysis

Bobby Habig*, Meredith M. Doellman, Kourtney Woods, Jonathan Olansen, and Elizabeth A. Archie

Department of Biological Sciences, University of Notre Dame, 100 Galvin Life Sciences Center, Notre Dame, IN 46556, USA

**Corresponding author; e-mail:* rhabig@nd.edu

Multi-level Meta-analysis in BRMS, All Male Studies

Meredith M. Doellman

February 1, 2018

This code includes the analysis of the full male data set. All other analyses were conducted under the same framework, and can be found along with the associated phylogenies and data sets in our Github repository https://github.com/mdoellma/social_status_parasitism_meta_analysis. Analyses were performed in R v3.4.0, brms v1.10.2, MCMCglmm v2.25.

Load the required libraries.

```
library(MCMCglmm)
```

```
## Loading required package: Matrix
## Loading required package: coda
## Loading required package: ape
```

```
library(brms)
```

```
## Loading required package: Rcpp
## Loading required package: ggplot2
## Loading 'brms' package (version 2.1.0). Useful instructions
## can be found by typing help('brms'). A more detailed introduction
## to the package is available through vignette('brms_overview').
## Run theme_set(theme_default()) to use the default bayesplot theme.
```

Load the data and format factors.

```
malesData <- read.table('male.all.txt',header=T,stringsAsFactors=F)
malesData
```

yi	vi	studyid	taxa	species	setting
2.3305	0.375870	13	Artiodactyla	Aepyceros_melampus	wild
-0.4727	0.091400	26	Aves	Agelaius_phoeniceus	wild
-0.0200	0.088900	26	Aves	Agelaius_phoeniceus	wild
0.0000	0.151982	17	Squamata	Anolis_sabanus	wild
3.3542	0.811641	1	Carnivora	Arctocephalus_forsteri	wild
1.5000	0.854200	5	Primates	Colobus_guereza_kikuyuensis	captive
5.5104	3.197000	8	Primates	Colobus_guereza_kikuyuensis	captive
-0.6992	0.303200	4	Primates	Colobus_vellerosus	wild
0.0000	0.000100	21	Perissodactyla	Equus_quagga	wild
-1.2676	0.108700	21	Perissodactyla	Equus_quagga	wild
-0.6214	0.095500	21	Perissodactyla	Equus_quagga	wild
-0.5579	0.094700	21	Perissodactyla	Equus_quagga	wild
0.0800	0.091300	21	Perissodactyla	Equus_quagga	wild
0.0542	0.091300	21	Perissodactyla	Equus_quagga	wild
-0.8231	0.098600	21	Perissodactyla	Equus_quagga	wild
-0.7363	0.097200	21	Perissodactyla	Equus_quagga	wild
-0.5972	0.362736	22	Primates	Eulemur_fulvus_rufus	wild
0.2527	0.352281	22	Primates	Eulemur_fulvus_rufus	wild
0.4556	0.357413	22	Primates	Eulemur_fulvus_rufus	wild

yi	vi	studyid	taxa	species	setting
1.0371	0.376764	22	Primates	Eulemur_fulvus_rufus	wild
-0.3221	0.344368	22	Primates	Eulemur_fulvus_rufus	wild
0.1995	0.342235	22	Primates	Eulemur_fulvus_rufus	wild
-0.3075	0.256900	25	Primates	Lophocebus_albigena	wild
-0.5113	0.262100	25	Primates	Lophocebus_albigena	wild
-0.5825	0.264600	25	Primates	Lophocebus_albigena	wild
0.2484	0.255900	25	Primates	Lophocebus_albigena	wild
-0.4079	0.259200	25	Primates	Lophocebus_albigena	wild
1.0409	0.371181	20	Aves	Malurus_cyaneus	wild
0.0280	0.078400	18	Artiodactyla	Ovis_canadensis	wild
1.6225	0.241600	3	Primates	Pan_troglodytes	wild
0.3242	0.184200	3	Primates	Pan_troglodytes	wild
0.9827	0.373600	15	Primates	Pan_troglodytes	wild
0.1388	0.148500	19	Primates	Papio_anubis	wild
0.6992	0.157200	19	Primates	Papio_anubis	wild
3.6065	1.312926	6	Primates	Papio_cynocephalus	wild
2.2323	0.844777	7	Primates	Papio_cynocephalus	wild
1.8778	0.753709	7	Primates	Papio_cynocephalus	wild
-0.3123	0.168700	11	Primates	Papio_cynocephalus	wild
0.4591	0.171100	11	Primates	Papio_cynocephalus	wild
0.4945	0.111400	16	Aves	Parus_major	wild
0.3450	0.109700	16	Aves	Parus_major	wild
0.4296	0.110600	16	Aves	Parus_major	wild
0.1263	0.143100	12	Aves	Passer_domesticus	captive
0.4491	0.121022	10	Squamata	Podarcis_melisellensis	wild
-0.2702	0.061757	14	Aves	Ptilonorhynchus_violaceus	wild
-1.7426	0.270600	2	Squamata	Sceloporus_occidentalis	wild
1.1198	0.414500	24	Carnivora	Suricata_suricatta	wild
1.1188	0.414400	24	Carnivora	Suricata_suricatta	wild
-0.0461	0.386000	24	Carnivora	Suricata_suricatta	wild
-0.1425	0.386400	24	Carnivora	Suricata_suricatta	wild
0.0000	0.000100	24	Carnivora	Suricata_suricatta	wild
-0.0359	0.386000	24	Carnivora	Suricata_suricatta	wild
0.5913	0.393900	24	Carnivora	Suricata_suricatta	wild
0.1505	0.386500	24	Carnivora	Suricata_suricatta	wild
-0.2700	0.387600	24	Carnivora	Suricata_suricatta	wild
0.7183	0.397700	24	Carnivora	Suricata_suricatta	wild
0.8144	0.121822	23	Carnivora	Suricata_suricatta	wild
0.6267	0.415807	23	Carnivora	Suricata_suricatta	wild
0.9873	0.166959	23	Carnivora	Suricata_suricatta	wild
0.2368	0.181790	23	Carnivora	Suricata_suricatta	wild
0.1330	0.138247	23	Carnivora	Suricata_suricatta	wild
-0.1366	0.143500	23	Carnivora	Suricata_suricatta	wild
0.4495	0.156189	23	Carnivora	Suricata_suricatta	wild
1.5368	0.202634	9	Actinopterygii	Symphodus_ocellatus	captive
0.2563	0.394707	9	Actinopterygii	Symphodus_ocellatus	captive
1.9170	0.082500	9	Actinopterygii	Symphodus_ocellatus	captive

```
malesData$phylo <- as.factor(malesData$species)
malesData$study <- as.factor(malesData$studyid)
malesData$set <- as.factor(malesData$setting)
```

```r
malesData$obs <- 1:nrow(malesData)
```

Load the phylogenetic tree and invert for brms.

```r
malesTree <- read.tree("../male.bladj")
inv.males <- MCMCglmm::inverseA(malesTree, nodes = "TIPS", scale = TRUE)
A <- solve(inv.males$Ainv)
rownames(A) <- rownames(inv.males$Ainv)
plot(malesTree,cex=0.7,show.node.label=F,label.offset=2)
```

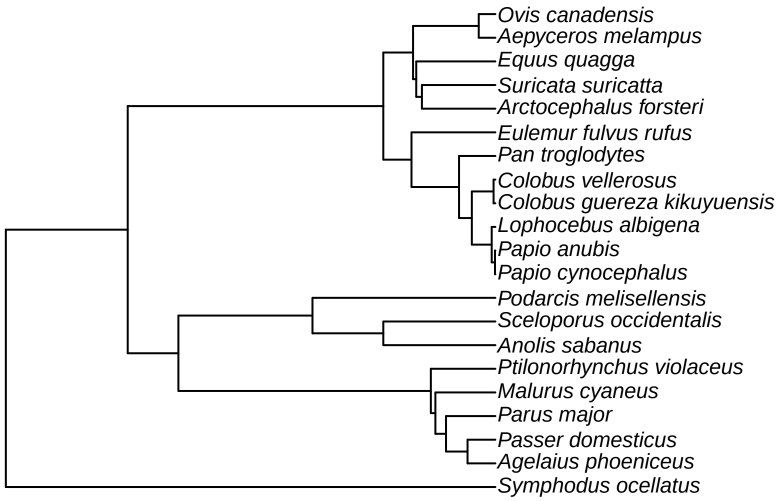

Calculate "typical" measurement error variance as Higgins & Thompson 2002, for use in heterogeneity calculations.

```r
malesData$wi <- 1/malesData$vi
s2m <- sum(malesData$wi*(length(malesData$wi)-1))/(sum(malesData$wi)^2-sum(malesData$wi^2))
```

Model 1

Multi-level meta-analytic model, including study as a random effect.

```r
model1 <- brm(yi | se(sqrt(vi)) ~ 1 + (1|study) + (1|obs),
                    data = malesData, family = gaussian(),
                    prior = c(prior(normal(0, 10), "Intercept"),
                              prior(student_t(3, 0, 10), "sd")),
                    control = list(adapt_delta = 0.998,max_treedepth = 15),
                    chains = 4, cores = 4, iter = 4000, warmup = 1000)
```

```
## Compiling the C++ model
```

```
## Start sampling
```

```r
summary(model1)
```

```
##  Family: gaussian
##   Links: mu = identity; sigma = identity
## Formula: yi | se(sqrt(vi)) ~ 1 + (1 | study) + (1 | obs)
##    Data: malesData (Number of observations: 66)
## Samples: 4 chains, each with iter = 4000; warmup = 1000; thin = 1;
##          total post-warmup samples = 12000
##     ICs: LOO = NA; WAIC = NA; R2 = NA
```

```
##
## Group-Level Effects:
## ~obs (Number of levels: 66)
##               Estimate Est.Error l-95% CI u-95% CI Eff.Sample Rhat
## sd(Intercept)     0.32      0.09     0.17     0.51       3795 1.00
##
## ~study (Number of levels: 26)
##               Estimate Est.Error l-95% CI u-95% CI Eff.Sample Rhat
## sd(Intercept)     0.88      0.23     0.49     1.40       2885 1.00
##
## Population-Level Effects:
##           Estimate Est.Error l-95% CI u-95% CI Eff.Sample Rhat
## Intercept     0.50      0.22     0.11     0.96       2588 1.00
##
## Samples were drawn using sampling(NUTS). For each parameter, Eff.Sample
## is a crude measure of effective sample size, and Rhat is the potential
## scale reduction factor on split chains (at convergence, Rhat = 1).
```

Calculate heterogeneity values, according to Nakagawa & Santos (2012).

```
temp <- as.mcmc(as.data.frame(model1)[,2:3])
total.var <- temp[,2]^2 + temp[,1]^2 + s2m
I2study <- 100*temp[,2]^2/(total.var)
I2units <- 100*temp[,1]^2/(total.var)
I2Tot <- 100*(temp[,2]^2 + temp[,1]^2)/(total.var)
```

Mean and HPD interval for study heterogeneity.

```
c(mean(I2study),HPDinterval(I2study))
```

```
## [1] 85.23101 64.92962 98.74384
```

Mean and HPD interval for residual heterogeneity.

```
c(mean(I2units),HPDinterval(I2units))
```

```
## [1] 13.9747945  0.8787796 33.7987371
```

Mean and HPD interval for total heterogeneity.

```
c(mean(I2Tot),HPDinterval(I2Tot))
```

```
## [1] 99.20581 98.49723 99.76730
```

Model 2

Multi-level meta-regression model, including study as a random effect and study setting as a moderator.

```
model2 <- brm(yi | se(sqrt(vi)) ~ set + (1|study) + (1|obs),
                    data = malesData, family = gaussian(),
                    prior = c(prior(normal(0, 10), "Intercept"),
                              prior(student_t(3, 0, 10), "sd")),
                    control = list(adapt_delta = 0.998,max_treedepth = 15),
                    chains = 4, cores = 4, iter = 4000, warmup = 1000)
```

```
## Compiling the C++ model
```

```
## Start sampling
```

```r
summary(model2)
```

```
##  Family: gaussian
##   Links: mu = identity; sigma = identity
## Formula: yi | se(sqrt(vi)) ~ set + (1 | study) + (1 | obs)
##    Data: malesData (Number of observations: 66)
## Samples: 4 chains, each with iter = 4000; warmup = 1000; thin = 1;
##          total post-warmup samples = 12000
##     ICs: LOO = NA; WAIC = NA; R2 = NA
##
## Group-Level Effects:
## ~obs (Number of levels: 66)
##               Estimate Est.Error l-95% CI u-95% CI Eff.Sample Rhat
## sd(Intercept)     0.32      0.09     0.16     0.52       2870 1.00
##
## ~study (Number of levels: 26)
##               Estimate Est.Error l-95% CI u-95% CI Eff.Sample Rhat
## sd(Intercept)     0.82      0.25     0.39     1.36       2427 1.00
##
## Population-Level Effects:
##           Estimate Est.Error l-95% CI u-95% CI Eff.Sample Rhat
## Intercept     1.36      0.58     0.25     2.54       6653 1.00
## setwild      -0.97      0.60    -2.20     0.22       6107 1.00
##
## Samples were drawn using sampling(NUTS). For each parameter, Eff.Sample
## is a crude measure of effective sample size, and Rhat is the potential
## scale reduction factor on split chains (at convergence, Rhat = 1).
```

Calculate heterogeneity values, according to Nakagawa & Santos (2012).

```r
temp <- as.mcmc(as.data.frame(model2)[,3:4])
total.var <- temp[,2]^2 + temp[,1]^2 + s2m
I2study <- 100*temp[,2]^2/(total.var)
I2units <- 100*temp[,1]^2/(total.var)
I2Tot <- 100*(temp[,2]^2 + temp[,1]^2)/(total.var)
```

Mean and HPD interval for study heterogeneity.

```r
c(mean(I2study),HPDinterval(I2study))
```

```
## [1] 82.66465 54.86297 98.56791
```

Mean and HPD interval for residual heterogeneity.

```r
c(mean(I2units),HPDinterval(I2units))
```

```
## [1] 16.4221535  0.4870039 43.1457976
```

Mean and HPD interval for total heterogeneity.

```r
c(mean(I2Tot),HPDinterval(I2Tot))
```

```
## [1] 99.08680 98.14848 99.75683
```

Model 3

Multi-level meta-analytic model, including study and species as random effects.

```r
model3 <- brm(yi | se(sqrt(vi)) ~ 1 + (1|study) + (1|species) + (1|obs),
                    data = malesData, family = gaussian(),
                    prior = c(prior(normal(0, 10), "Intercept"),
                              prior(student_t(3, 0, 10), "sd")),
                    control = list(adapt_delta = 0.998,max_treedepth = 15),
                    chains = 4, cores = 4, iter = 4000, warmup = 1000)
```

```
## Compiling the C++ model
```

```
## Start sampling
```

```r
summary(model3)
```

```
##  Family: gaussian
##   Links: mu = identity; sigma = identity
## Formula: yi | se(sqrt(vi)) ~ 1 + (1 | study) + (1 | species) + (1 | obs)
##    Data: malesData (Number of observations: 66)
## Samples: 4 chains, each with iter = 4000; warmup = 1000; thin = 1;
##          total post-warmup samples = 12000
##     ICs: LOO = NA; WAIC = NA; R2 = NA
##
## Group-Level Effects:
## ~obs (Number of levels: 66)
##               Estimate Est.Error l-95% CI u-95% CI Eff.Sample Rhat
## sd(Intercept)     0.32      0.09     0.17     0.51       4977 1.00
##
## ~species (Number of levels: 21)
##               Estimate Est.Error l-95% CI u-95% CI Eff.Sample Rhat
## sd(Intercept)     0.57      0.32     0.04     1.22       1759 1.00
##
## ~study (Number of levels: 26)
##               Estimate Est.Error l-95% CI u-95% CI Eff.Sample Rhat
## sd(Intercept)     0.65      0.31     0.06     1.27       1701 1.00
##
## Population-Level Effects:
##           Estimate Est.Error l-95% CI u-95% CI Eff.Sample Rhat
## Intercept     0.46      0.23     0.02     0.94       6046 1.00
##
## Samples were drawn using sampling(NUTS). For each parameter, Eff.Sample
## is a crude measure of effective sample size, and Rhat is the potential
## scale reduction factor on split chains (at convergence, Rhat = 1).
```

Calculate heterogeneity values, according to Nakagawa & Santos (2012).

```r
temp <- as.mcmc(as.data.frame(model3)[,2:4])
total.var <- temp[,3]^2 + temp[,2]^2 + temp[,1]^2 + s2m
I2study <- 100*temp[,3]^2/(total.var)
I2species <- 100*temp[,2]^2/(total.var)
I2units <- 100*temp[,1]^2/(total.var)
I2Tot <- 100*(temp[,3]^2 + temp[,2]^2 + temp[,1]^2)/(total.var)
```

Mean and HPD interval for study heterogeneity.

```r
c(mean(I2study),HPDinterval(I2study))
```

```
## [1] 4.739831e+01 7.732558e-06 9.029739e+01
```

Mean and HPD interval for species heterogeneity.

```r
c(mean(I2species),HPDinterval(I2species))
```

```
## [1] 3.938687e+01 1.827629e-08 8.563477e+01
```

Mean and HPD interval for residual heterogeneity.

```r
c(mean(I2units),HPDinterval(I2units))
```

```
## [1] 12.4979225  0.8785268 30.5248067
```

Mean and HPD interval for total heterogeneity.

```r
c(mean(I2Tot),HPDinterval(I2Tot))
```

```
## [1] 99.28310 98.61237 99.81866
```

Model 4

Multi-level meta-regression model, including study and species as random effects and study setting as a moderator.

```r
model4 <- brm(yi | se(sqrt(vi)) ~ set + (1|study) + (1|species) + (1|obs),
                    data = malesData, family = gaussian(),
                    prior = c(prior(normal(0, 10), "Intercept"),
                              prior(student_t(3, 0, 10), "sd")),
                    control = list(adapt_delta = 0.998,max_treedepth = 15),
                    chains = 4, cores = 4, iter = 4000, warmup = 1000)
```

```
## Compiling the C++ model
```

```
## Start sampling
```

```r
summary(model4)
```

```
##  Family: gaussian
##   Links: mu = identity; sigma = identity
## Formula: yi | se(sqrt(vi)) ~ set + (1 | study) + (1 | species) + (1 | obs)
##    Data: malesData (Number of observations: 66)
## Samples: 4 chains, each with iter = 4000; warmup = 1000; thin = 1;
##          total post-warmup samples = 12000
##     ICs: LOO = NA; WAIC = NA; R2 = NA
##
## Group-Level Effects:
## ~obs (Number of levels: 66)
##               Estimate Est.Error l-95% CI u-95% CI Eff.Sample Rhat
## sd(Intercept)     0.32      0.09     0.17     0.51       4871 1.00
##
## ~species (Number of levels: 21)
##               Estimate Est.Error l-95% CI u-95% CI Eff.Sample Rhat
## sd(Intercept)     0.53      0.30     0.03     1.16       2660 1.00
##
## ~study (Number of levels: 26)
##               Estimate Est.Error l-95% CI u-95% CI Eff.Sample Rhat
## sd(Intercept)     0.61      0.31     0.06     1.24       2013 1.00
##
## Population-Level Effects:
```

```
##            Estimate Est.Error l-95% CI u-95% CI Eff.Sample Rhat
## Intercept     1.30      0.61     0.14     2.53      12000 1.00
## setwild      -0.97      0.64    -2.26     0.31      11311 1.00
##
## Samples were drawn using sampling(NUTS). For each parameter, Eff.Sample
## is a crude measure of effective sample size, and Rhat is the potential
## scale reduction factor on split chains (at convergence, Rhat = 1).
```

Calculate heterogeneity values, according to Nakagawa & Santos (2012).

```
temp <- as.mcmc(as.data.frame(model4)[,3:5])
total.var <- temp[,3]^2 + temp[,2]^2 + temp[,1]^2 + s2m
I2study <- 100*temp[,3]^2/(total.var)
I2species <- 100*temp[,2]^2/(total.var)
I2units <- 100*temp[,1]^2/(total.var)
I2Tot <- 100*(temp[,3]^2 + temp[,2]^2 + temp[,1]^2)/(total.var)
```

Mean and HPD interval for study heterogeneity.

```
c(mean(I2study),HPDinterval(I2study))
```

```
## [1] 4.631071e+01 1.089749e-04 9.009240e+01
```

Mean and HPD interval for species heterogeneity.

```
c(mean(I2species),HPDinterval(I2species))
```

```
## [1] 3.823645e+01 1.085320e-05 8.396771e+01
```

Mean and HPD interval for residual heterogeneity.

```
c(mean(I2units),HPDinterval(I2units))
```

```
## [1] 14.6315412  0.6364677 37.4402561
```

Mean and HPD interval for total heterogeneity.

```
c(mean(I2Tot),HPDinterval(I2Tot))
```

```
## [1] 99.1787 98.3795 99.8093
```

Model 5

Multi-level meta-analytic model, including study, species and phylogeny as random effects.

```
model5 <- brm(yi | se(sqrt(vi)) ~ 1 + (1|study) + (1|species) + (1|phylo) + (1|obs),
                    data = malesData, family = gaussian(),
                    cov_ranef = list(phylo = A),
                    prior = c(prior(normal(0, 10), "Intercept"),
                              prior(student_t(3, 0, 10), "sd")),
                    control = list(adapt_delta = 0.998,max_treedepth = 15),
                    chains = 4, cores = 4, iter = 4000, warmup = 1000)
```

```
## Compiling the C++ model
```

```
## Start sampling
```

```
summary(model5)
```

```
##  Family: gaussian
##   Links: mu = identity; sigma = identity
```

```
## Formula: yi | se(sqrt(vi)) ~ 1 + (1 | study) + (1 | species) + (1 | phylo) + (1 | obs)
##    Data: malesData (Number of observations: 66)
## Samples: 4 chains, each with iter = 4000; warmup = 1000; thin = 1;
##          total post-warmup samples = 12000
##     ICs: LOO = NA; WAIC = NA; R2 = NA
##
## Group-Level Effects:
## ~obs (Number of levels: 66)
##               Estimate Est.Error l-95% CI u-95% CI Eff.Sample Rhat
## sd(Intercept)     0.32      0.09     0.17     0.51       3794 1.00
##
## ~phylo (Number of levels: 21)
##               Estimate Est.Error l-95% CI u-95% CI Eff.Sample Rhat
## sd(Intercept)     0.60      0.47     0.02     1.77       3249 1.00
##
## ~species (Number of levels: 21)
##               Estimate Est.Error l-95% CI u-95% CI Eff.Sample Rhat
## sd(Intercept)     0.53      0.31     0.03     1.18       1860 1.00
##
## ~study (Number of levels: 26)
##               Estimate Est.Error l-95% CI u-95% CI Eff.Sample Rhat
## sd(Intercept)     0.64      0.31     0.06     1.28       1703 1.00
##
## Population-Level Effects:
##           Estimate Est.Error l-95% CI u-95% CI Eff.Sample Rhat
## Intercept     0.48      0.52    -0.56     1.60       5302 1.00
##
## Samples were drawn using sampling(NUTS). For each parameter, Eff.Sample
## is a crude measure of effective sample size, and Rhat is the potential
## scale reduction factor on split chains (at convergence, Rhat = 1).
```

Calculate heterogeneity values, according to Nakagawa & Santos (2012).

```
temp <- as.mcmc(as.data.frame(model5)[,2:5])
total.var <- temp[,4]^2 + temp[,3]^2 + temp[,2]^2 + temp[,1]^2 + s2m
I2study <- 100*temp[,4]^2/(total.var)
I2species <- 100*temp[,3]^2/(total.var)
I2phylo <- 100*temp[,2]^2/(total.var)
I2units <- 100*temp[,1]^2/(total.var)
I2Tot <- 100*(temp[,4]^2 + temp[,3]^2 + temp[,2]^2 + temp[,1]^2)/(total.var)
```

Mean and HPD interval for study heterogeneity.

```
c(mean(I2study),HPDinterval(I2study))
```

```
## [1] 3.571587e+01 5.669157e-06 8.179713e+01
```

Mean and HPD interval for species heterogeneity.

```
c(mean(I2species),HPDinterval(I2species))
```

```
## [1] 2.662872e+01 1.572625e-05 7.335635e+01
```

Mean and HPD interval for species heterogeneity.

```
c(mean(I2phylo),HPDinterval(I2phylo))
```

```
## [1] 2.785925e+01 3.393946e-07 7.527218e+01
```

Mean and HPD interval for residual heterogeneity.

```
c(mean(I2units),HPDinterval(I2units))
```

```
## [1]  9.265998  0.562410 24.489617
```

Mean and HPD interval for total heterogeneity.

```
c(mean(I2Tot),HPDinterval(I2Tot))
```

```
## [1] 99.46984 98.91546 99.91163
```

Model 6

Multi-level meta-regression model, including study, species and phylogeny as random effects and study setting as a moderator.

```
model6 <- brm(yi | se(sqrt(vi)) ~ set + (1|study) + (1|species) + (1|phylo) + (1|obs),
                    data = malesData, family = gaussian(),
                    cov_ranef = list(phylo = A),
                    prior = c(prior(normal(0, 10), "Intercept"),
                              prior(student_t(3, 0, 10), "sd")),
                    control = list(adapt_delta = 0.998,max_treedepth = 15),
                    chains = 4, cores = 4, iter = 4000, warmup = 1000)
```

```
## Compiling the C++ model
```

```
## Start sampling
```

```
summary(model6)
```

```
##  Family: gaussian
##   Links: mu = identity; sigma = identity
## Formula: yi | se(sqrt(vi)) ~ set + (1 | study) + (1 | species) + (1 | phylo) + (1 | obs)
##    Data: malesData (Number of observations: 66)
## Samples: 4 chains, each with iter = 4000; warmup = 1000; thin = 1;
##          total post-warmup samples = 12000
##     ICs: LOO = NA; WAIC = NA; R2 = NA
##
## Group-Level Effects:
## ~obs (Number of levels: 66)
##               Estimate Est.Error l-95% CI u-95% CI Eff.Sample Rhat
## sd(Intercept)     0.32      0.09     0.17     0.50       4838 1.00
##
## ~phylo (Number of levels: 21)
##               Estimate Est.Error l-95% CI u-95% CI Eff.Sample Rhat
## sd(Intercept)     0.58      0.46     0.02     1.74       3610 1.00
##
## ~species (Number of levels: 21)
##               Estimate Est.Error l-95% CI u-95% CI Eff.Sample Rhat
## sd(Intercept)     0.51      0.31     0.03     1.16       2251 1.00
##
## ~study (Number of levels: 26)
##               Estimate Est.Error l-95% CI u-95% CI Eff.Sample Rhat
## sd(Intercept)     0.62      0.31     0.05     1.25       1488 1.00
##
## Population-Level Effects:
```

```
##           Estimate Est.Error l-95% CI u-95% CI Eff.Sample Rhat
## Intercept     1.30      0.75    -0.20     2.85       7442 1.00
## setwild      -1.04      0.71    -2.50     0.33       7520 1.00
##
## Samples were drawn using sampling(NUTS). For each parameter, Eff.Sample
## is a crude measure of effective sample size, and Rhat is the potential
## scale reduction factor on split chains (at convergence, Rhat = 1).
```

Calculate heterogeneity values, according to Nakagawa & Santos (2012).

```
temp <- as.mcmc(as.data.frame(model6)[,3:6])
total.var <- temp[,4]^2 + temp[,3]^2 + temp[,2]^2 + temp[,1]^2 + s2m
I2study <- 100*temp[,4]^2/(total.var)
I2species <- 100*temp[,3]^2/(total.var)
I2phylo <- 100*temp[,2]^2/(total.var)
I2units <- 100*temp[,1]^2/(total.var)
I2Tot <- 100*(temp[,4]^2 + temp[,3]^2 + temp[,2]^2 + temp[,1]^2)/(total.var)
```

Mean and HPD interval for study heterogeneity.

```
c(mean(I2study),HPDinterval(I2study))
```

```
## [1] 3.514929e+01 8.294635e-07 8.003261e+01
```

Mean and HPD interval for species heterogeneity.

```
c(mean(I2species),HPDinterval(I2species))
```

```
## [1] 2.626756e+01 2.800581e-06 7.294572e+01
```

Mean and HPD interval for species heterogeneity.

```
c(mean(I2phylo),HPDinterval(I2phylo))
```

```
## [1] 2.790903e+01 1.801154e-07 7.623098e+01
```

Mean and HPD interval for residual heterogeneity.

```
c(mean(I2units),HPDinterval(I2units))
```

```
## [1] 10.0958340  0.3187851 26.9829771
```

Mean and HPD interval for total heterogeneity.

```
c(mean(I2Tot),HPDinterval(I2Tot))
```

```
## [1] 99.42172 98.75954 99.90567
```

Conduct K-fold cross-validation for each model, with K=10.

```
k1 <- kfold(model1,K=10)
```

```
## Fitting model 1 out of 10

## The desired updates require recompling the model

## Start sampling

## Fitting model 2 out of 10

## The desired updates require recompling the model

## Start sampling

## Fitting model 3 out of 10
```

```
## The desired updates require recompling the model
## Start sampling
## Fitting model 4 out of 10
## The desired updates require recompling the model
## Start sampling
## Fitting model 5 out of 10
## The desired updates require recompling the model
## Start sampling
## Fitting model 6 out of 10
## The desired updates require recompling the model
## Start sampling
## Fitting model 7 out of 10
## The desired updates require recompling the model
## Start sampling
## Fitting model 8 out of 10
## The desired updates require recompling the model
## Start sampling
## Fitting model 9 out of 10
## The desired updates require recompling the model
## Start sampling
## Fitting model 10 out of 10
## The desired updates require recompling the model
## Start sampling
```

```
k2 <- kfold(model2,K=10)
```

```
## Fitting model 1 out of 10
## The desired updates require recompling the model
## Start sampling
## Fitting model 2 out of 10
## The desired updates require recompling the model
## Start sampling
## Fitting model 3 out of 10
## The desired updates require recompling the model
## Start sampling
## Fitting model 4 out of 10
## The desired updates require recompling the model
## Start sampling
```

```
## Fitting model 5 out of 10
## The desired updates require recompling the model
## Start sampling
## Fitting model 6 out of 10
## The desired updates require recompling the model
## Start sampling
## Fitting model 7 out of 10
## The desired updates require recompling the model
## Start sampling
## Fitting model 8 out of 10
## The desired updates require recompling the model
## Start sampling
## Fitting model 9 out of 10
## The desired updates require recompling the model
## Start sampling
## Fitting model 10 out of 10
## The desired updates require recompling the model
## Start sampling
```

```r
k3 <- kfold(model3,K=10)
```

```
## Fitting model 1 out of 10
## The desired updates require recompling the model
## Start sampling
## Fitting model 2 out of 10
## The desired updates require recompling the model
## Start sampling
## Fitting model 3 out of 10
## The desired updates require recompling the model
## Start sampling
## Fitting model 4 out of 10
## The desired updates require recompling the model
## Start sampling
## Fitting model 5 out of 10
## The desired updates require recompling the model
## Start sampling
## Fitting model 6 out of 10
## The desired updates require recompling the model
```

```
## Start sampling
## Fitting model 7 out of 10
## The desired updates require recompling the model
## Start sampling
## Fitting model 8 out of 10
## The desired updates require recompling the model
## Start sampling
## Fitting model 9 out of 10
## The desired updates require recompling the model
## Start sampling
## Fitting model 10 out of 10
## The desired updates require recompling the model
## Start sampling
```

```r
k4 <- kfold(model4,K=10)
```

```
## Fitting model 1 out of 10
## The desired updates require recompling the model
## Start sampling
## Fitting model 2 out of 10
## The desired updates require recompling the model
## Start sampling
## Fitting model 3 out of 10
## The desired updates require recompling the model
## Start sampling
## Fitting model 4 out of 10
## The desired updates require recompling the model
## Start sampling
## Fitting model 5 out of 10
## The desired updates require recompling the model
## Start sampling
## Fitting model 6 out of 10
## The desired updates require recompling the model
## Start sampling
## Fitting model 7 out of 10
## The desired updates require recompling the model
## Start sampling
## Fitting model 8 out of 10
```

```
## The desired updates require recompling the model
## Start sampling
## Fitting model 9 out of 10
## The desired updates require recompling the model
## Start sampling
## Fitting model 10 out of 10
## The desired updates require recompling the model
## Start sampling
```

```r
k5 <- kfold(model5,K=10)
```

```
## Fitting model 1 out of 10
## The desired updates require recompling the model
## Start sampling
## Fitting model 2 out of 10
## The desired updates require recompling the model
## Start sampling
## Fitting model 3 out of 10
## The desired updates require recompling the model
## Start sampling
## Fitting model 4 out of 10
## The desired updates require recompling the model
## Start sampling
## Fitting model 5 out of 10
## The desired updates require recompling the model
## Start sampling
## Fitting model 6 out of 10
## The desired updates require recompling the model
## Start sampling
## Fitting model 7 out of 10
## The desired updates require recompling the model
## Start sampling
## Fitting model 8 out of 10
## The desired updates require recompling the model
## Start sampling
## Fitting model 9 out of 10
## The desired updates require recompling the model
## Start sampling
```

```
## Fitting model 10 out of 10
## The desired updates require recompling the model
## Start sampling
```

```r
k6 <- kfold(model6,K=10)
```

```
## Fitting model 1 out of 10
## The desired updates require recompling the model
## Start sampling
## Fitting model 2 out of 10
## The desired updates require recompling the model
## Start sampling
## Fitting model 3 out of 10
## The desired updates require recompling the model
## Start sampling
## Fitting model 4 out of 10
## The desired updates require recompling the model
## Start sampling
## Fitting model 5 out of 10
## The desired updates require recompling the model
## Start sampling
## Fitting model 6 out of 10
## The desired updates require recompling the model
## Start sampling
## Fitting model 7 out of 10
## The desired updates require recompling the model
## Start sampling
## Fitting model 8 out of 10
## The desired updates require recompling the model
## Start sampling
## Fitting model 9 out of 10
## The desired updates require recompling the model
## Start sampling
## Fitting model 10 out of 10
## The desired updates require recompling the model
## Start sampling
```

K-fold information criterion for each model. The lowest value indicates the best model fit.

```r
k1$kfoldic
```

```
## [1] 150.1088
```

```r
k2$kfoldic
```

```
## [1] 150.9112
```

```r
k3$kfoldic
```

```
## [1] 156.6812
```

```r
k4$kfoldic
```

```
## [1] 154.0561
```

```r
k5$kfoldic
```

```
## [1] 156.6449
```

```r
k6$kfoldic
```

```
## [1] 154.7448
```
